# Azirinium ylides from α-diazoketones and 2*H*-azirines on the route to 2*H*-1,4-oxazines: three-membered ring opening vs 1,5-cyclization

**DOI:** 10.3762/bjoc.11.35

**Published:** 2015-03-02

**Authors:** Nikolai V Rostovskii, Mikhail S Novikov, Alexander F Khlebnikov, Galina L Starova, Margarita S Avdontseva

**Affiliations:** 1 Institute of Chemistry, Saint-Petersburg State University, Universitetskii pr. 26, 198504 St. Petersburg, Russia; 2 Institute of Earth Science, Saint-Petersburg State University, Universitetskaya nab. 7–9, 199034 St. Petersburg, Russia

**Keywords:** 2-azabuta-1,3-dienes, azirines, diazo compounds, nitrogen ylides, 2*H*-1,4-oxazines

## Abstract

Strained azirinium ylides derived from 2*H*-azirines and α-diazoketones under Rh(II)-catalysis can undergo either irreversible ring opening across the N–C2 bond to 2-azabuta-1,3-dienes that further cyclize to 2*H*-1,4-oxazines or reversibly undergo a 1,5-cyclization to dihydroazireno[2,1-*b*]oxazoles. Dihydroazireno[2,1-*b*]oxazoles derived from 3-aryl-2*H*-azirines and 3-diazoacetylacetone or ethyl diazoacetoacetate are able to cycloadd to acetyl(methyl)ketene generated from 3-diazoacetylacetone under Rh(II) catalysis to give 4,6-dioxa-1-azabicyclo[3.2.1]oct-2-ene and/or 5,7-dioxa-1-azabicyclo[4.3.1]deca-3,8-diene-2-one derivatives. According to DFT calculations (B3LYP/6-31+G(d,p)), the cycloaddition can involve two modes of nucleophilic attack of the dihydroazireno[2,1-*b*]oxazole intermediate on acetyl(methyl)ketene followed by aziridine ring opening into atropoisomeric oxazolium betaines and cyclization. Azirinium ylides generated from 2,3-di- and 2,2,3-triaryl-substituted azirines give rise to only 2-azabuta-1,3-dienes and/or 2*H*-1,4-oxazines.

## Introduction

**Scheme 1:**
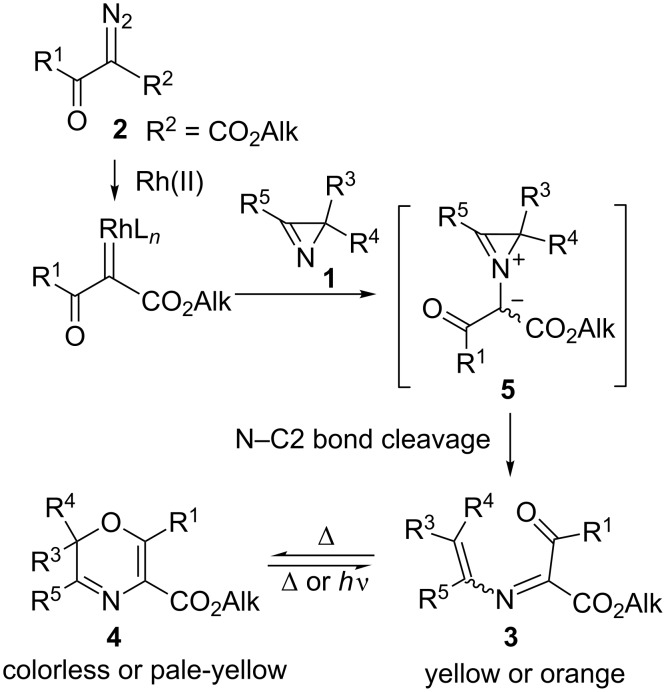
Rh(II)-catalyzed synthesis of photochromic 2*H*-1,4-oxazines from 2*H*-azirines and α-diazo-β-ketoesters.

2*H*-Azirines are unique strained compounds which have found various applications in organic synthesis due to their ability to react both with retention and opening of the three-membered ring. Even though each of the three bonds in the azirine ring can be cleaved under certain conditions, the majority of synthetic applications of these compounds imply cleavage of either the N–C2 or N–C3 bond. In these syntheses 2*H*-azirines serve as C–C–N three-atomic building blocks for the construction of both acyclic compounds, such as nonproteinogenic amino acids and peptides [1–3], and various 4–7-membered heterocycles [4–11]. The reactions of azirines with acylketenes [12–13], carboxylic acids [14] and amino acids [1] proceed via N–C3 bond cleavage to afford 5,7-dioxa-1-azabicyclo[4.4.1]undeca-3,8-diene derivatives, ketamides, and aminoamides, respectively. On the other hand, rhodium carbenoids derived from α-diazocarbonyl compounds transform 2*H*-azirines **1** to azirinium ylides **5** (Scheme 1) which undergo facile N–C2 bond cleavage to give 2-azabuta-1,3-dienes **3**. Recently we showed that the use of α-diazo-β-ketoesters **2** in these reactions, which are finished by 1,6-cyclization of azadienes onto the keto group, provides a rapid access to non-fused 2*H*-1,4-oxazine-5-carboxylates **4**, a new type of non-spirocyclic 1,4-oxazine photochromes [15].

In the search for new compounds with useful photochromic characteristics and a high fatigue resistance we focused on 5-unsubstituted, 5-aryl-, and 5-acyl-substituted 2*H*-1,4-oxazines. They could, in principle, be prepared from azirines and α-diazo ketones or 2-diazo-1,3-diketones via ring opening across the N–C2 bond in the intermediate azirinium ylides to form 2-azabuta-1,3-dienes and subsequent cyclization of the latter into 2*H*-1,4-oxazines. In the work reported herein we studied Rh(II)-catalyzed reactions of alkyl-, aryl-, and hetaryl-substituted 2*Н*-azirines with α-diazo-α-phenylacetone (**2a**), α-diazo-4-chloroacetophenone (**2b**), and 3-diazoacetylacetone (**2c**). A new type of transformation of azirinium ylides, specifically 1,5-cyclization into dihydroazireno[2,1-*b*]oxazoles capable of cycloadding to acyl ketenes, was discovered in the study on the reactivity of 3-monosubstituted azirines toward 3-diazoacetylacetone (**2c**) under catalytic conditions. The competition between ring opening and 1,5-cyclization in azirinium ylides, as well as the mechanism of trapping of dihydroazireno[2,1-*b*]oxazole intermediates by acetyl(methyl)ketene were investigated by the DFT method.

## Results and Discussion

**Scheme 2:**
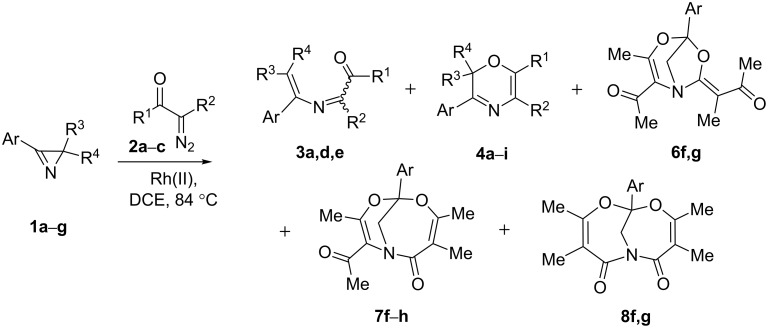
Rh(II)-catalyzed reaction of azirines **1a**−**g** with diazo compounds **2a**–**c**.

Rhodium(II) carbenoids generated from α-diazoketones or 2-diazo-1,3-diketones react with various nitrogen-containing compounds, such as amines [16], amides [17–19], and nitriles [20–21], to give N–H insertion products or N- or N,O-heterocyclic systems. The reactivity of acyl- or diacyl-substituted Rh(II) carbenoids toward an sp^2^-hybridized nitrogen [22–24] is much less studied, while examples of their reactions with 2*H*-azirines are unknown at all. Our study of the chemical behavior of 2*H*-azirines under Rh(II)-catalyzed decomposition of diazoketones was started with searching for optimal conditions for the catalytic reaction of 2,3-diphenyl-2*H*-azirine (**1a**) with 1-diazo**-**1**-**phenylpropan-2-one (**2a**), leading to oxazine **4a**. The most successful procedure involving addition of Rh_2_(OAc)_4_ to a boiling 1:1.25 mixture of azirine **1a** and diazo compound **2a** in 1,2-dichloroethane (DCE) (procedure A) gave rise to oxazine **4a** and azadiene *Z*-**3a** in 60 and 7% yields, respectively (Scheme 2, Table 1, entry 1). It was also shown that azadiene *Z*-**3a** did not cyclize into oxazine **4a** under the reaction conditions. According to our previous computational and experimental results for transformations of 3,4-diphenyl-substituted 2-azabuta-1,3-dienes derived from α-diazo-β-ketoesters, oxazine **4a** formed via rapid cyclization of azadiene *E*-**3a** [15], whereas 2-azabuta-1,3-dienes with the C=C bond in *Z*-configuration have a sufficiently high activation barrier for cyclization and are usually stable up to 90–100 °C.

**Table 1:** Rh(II)-catalyzed reaction of azirines **1a**–**g** with diazo compounds **2a**–**c** (method A, DCE, 84 °C).

Entry	**1**	Ar	R^1^	R^2^	R^3^	R^4^	**2**	Catalyst	Isolated yields, %				

									3	4	6	7	8

1	**1a**	Ph	Me	Ph	H	Ph	**2a**	Rh_2_(OAc)_4_	7 (*Z*-**a**)^a^	60 (**a**)			
2	**1b**	Ph	Me	Ph	H	1-Bt^b^	**2a**	Rh_2_(OAc)_4_		55 (**b**)			
3	**1c**	Ph	Me	Ph	Me	Me	**2a**	Rh_2_(OAc)_4_		58 (**c**)			
4	**1d**	Ph	Me	Ph	Ph	Ph	**2a**	Rh_2_(OAc)_4_	75 (**d**)^c^	7 (**d**)			
5	**1c**	Ph	4-ClC_6_H_4_	H	Me	Me	**2b**	Rh_2_(OAc)_4_	43 (**e**)^d^	67 (**e**)^e^			
6	**1e**	4-MeC_6_H_4_	Me	MeCO	H	H	**2c**	Rh_2_(OAc)_4_		17 (**f**)	–^f^	–^f^	–^f^
7	**1e**	4-MeC_6_H_4_	Me	MeCO	H	H	**2c**	Rh_2_(Oct)_4_		22 (**f**)^g^	23 (**f**)	7 (**f**)	4 (**f**)
8	**1f**	4-MeOC_6_H_4_	Me	MeCO	H	H	**2c**	Rh_2_(Oct)_4_		18 (**g**)	26 (**g**)^h^	6 (**g**)	4 (**g**)^h^
9	**1g**	4-NO_2_C_6_H_4_	Me	MeCO	H	H	**2c**	Rh_2_(Oct)_4_		58 (**h**)		13 (**h**)	
10	**1a**	Ph	Me	MeCO	H	Ph	**2c**	Rh_2_(OAc)_4_		47 (**i**)*^i^*			

^a^Compound *Z***-3a** is a 1.1:1 mixture of isomers across C=N bond. ^b^1*H-*Benzotriazol-1-yl. ^c^Compound **3d** is a 1.2:1 mixture of isomers across C=N bond. ^d^Reaction temperature 60 °C. ^e^Obtained from azadiene **3e** at 84 °C. ^f^Not isolated. ^g^Et_3_N-doped eluent for chromatography was used. ^h^The yield was determined by ^1^H NMR spectroscopy with acenaphtene as internal standard. The compound decomposes on silica gel. ^i^The reaction was carried out according to method B.

Oxazine **4b** was synthesized in a similar way from azirine **1b** and diazo compound **2a** (Table 1, entry 2), but in this case no traces of azadiene *Z*-**3b** were detected. 4,4-Dimethyl- and 4,4-diphenyl-2-azabuta-1,3-dienes **3c**,**d** derived from azirines **1c**,**d** (Table 1, entries 3 and 4) showed a different behavior. The former rapidly cyclized into oxazine **4c**, isolated in 58% yield. On the contrary, oxazine **4d** was obtained in as low as 7% yield, while the main reaction product was azadiene **3d**. Moreover, when dissolved separately in CDCl_3_, compounds **3d** and **4d** both converted into 4.5:1 **3d** + **4d** equilibrium mixtures after a week at room temperature.

**Figure 1:**
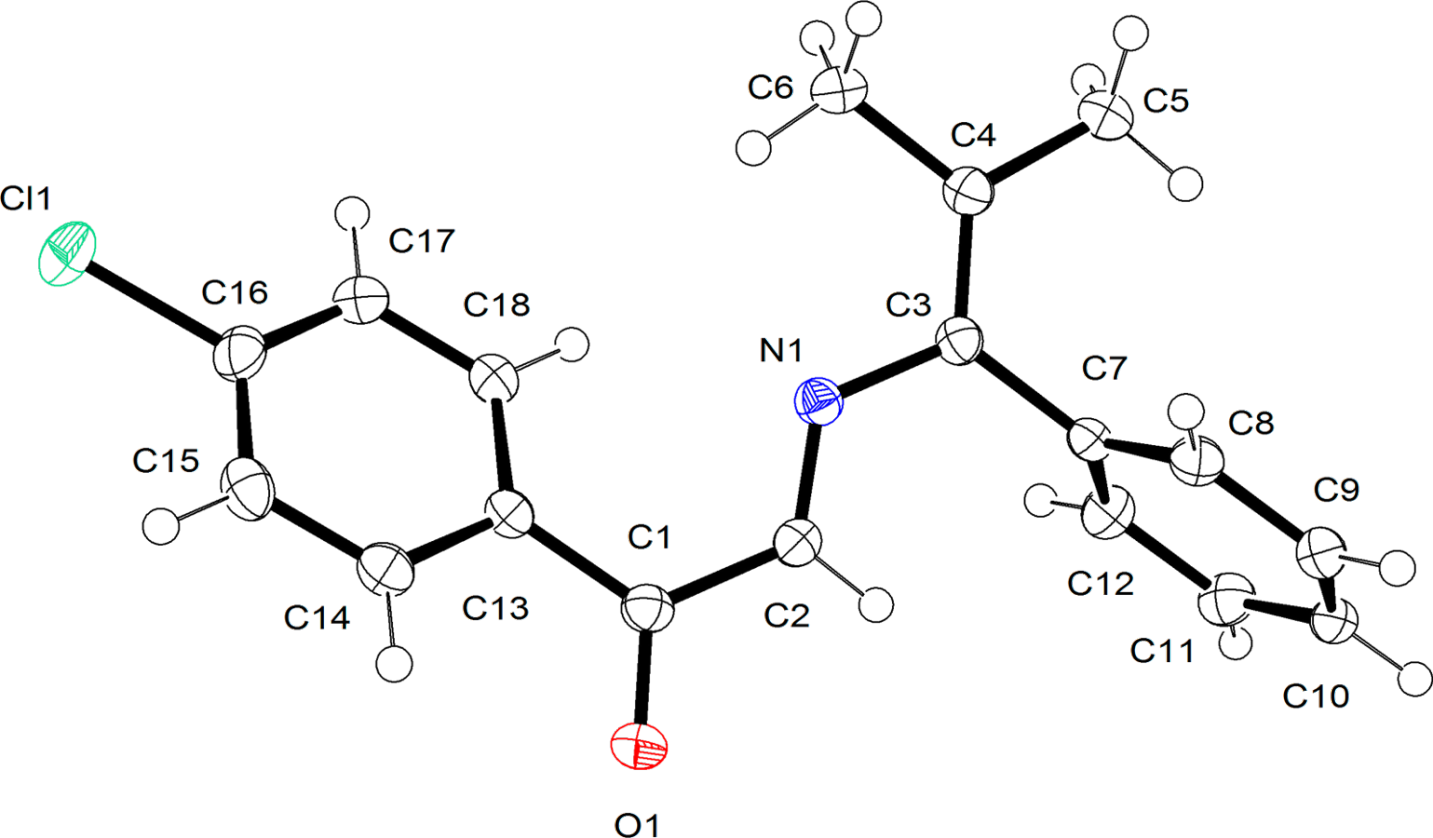
X-ray crystal structure of azadiene **3e**.

To prepare a C5-unsubstituted 1,4-oxazine derivative, we synthesized α-diazo-4-chloroacetophenone (**2b**) according to the published procedure [25]. Diazo compound **2b** slowly decomposes in the presence of Rh_2_(OAc)_4_ and 2,2-dimethyl-3-phenylazirine (**1c**) even at room temperature, but no conversion of the latter was observed according to ^1^H NMR spectroscopy. A satisfactory result was obtained, when the reaction was carried out at 60 °С: azirine **1c** was completely consumed to give azadiene **3e** isolated by column chromatography in 43% yield (Table 1, entry 5). The structure of azadiene **3e** was confirmed by X-ray diffraction analysis (Figure 1). It should be noted that azadiene **3e** in crystal exists in the s-*trans-*conformation across the single С–N bond (the С2–N1–С3–C4 dihedral angle is 4.3°, Figure 1), unlike its C4-aryl-substituted analogs with the angle of 73–75° [26]. The *Е*-configuration of the С=N bond, unfavorable for cyclization into 1,4-oxazine, explains the enhanced thermal stability of compound **3e**.

Heating azadiene **3e** in DCE under reflux (84 °C) for 3.5 h gave a 6:1 equilibrium mixture of 1,4-oxazine **4e** and azadiene **3e** (according to ^1^Н NMR spectroscopy). Oxazine **4e** is stable enough at room temperature to be isolated by column chromatography (Table 1, entry 5).

**Figure 2:**
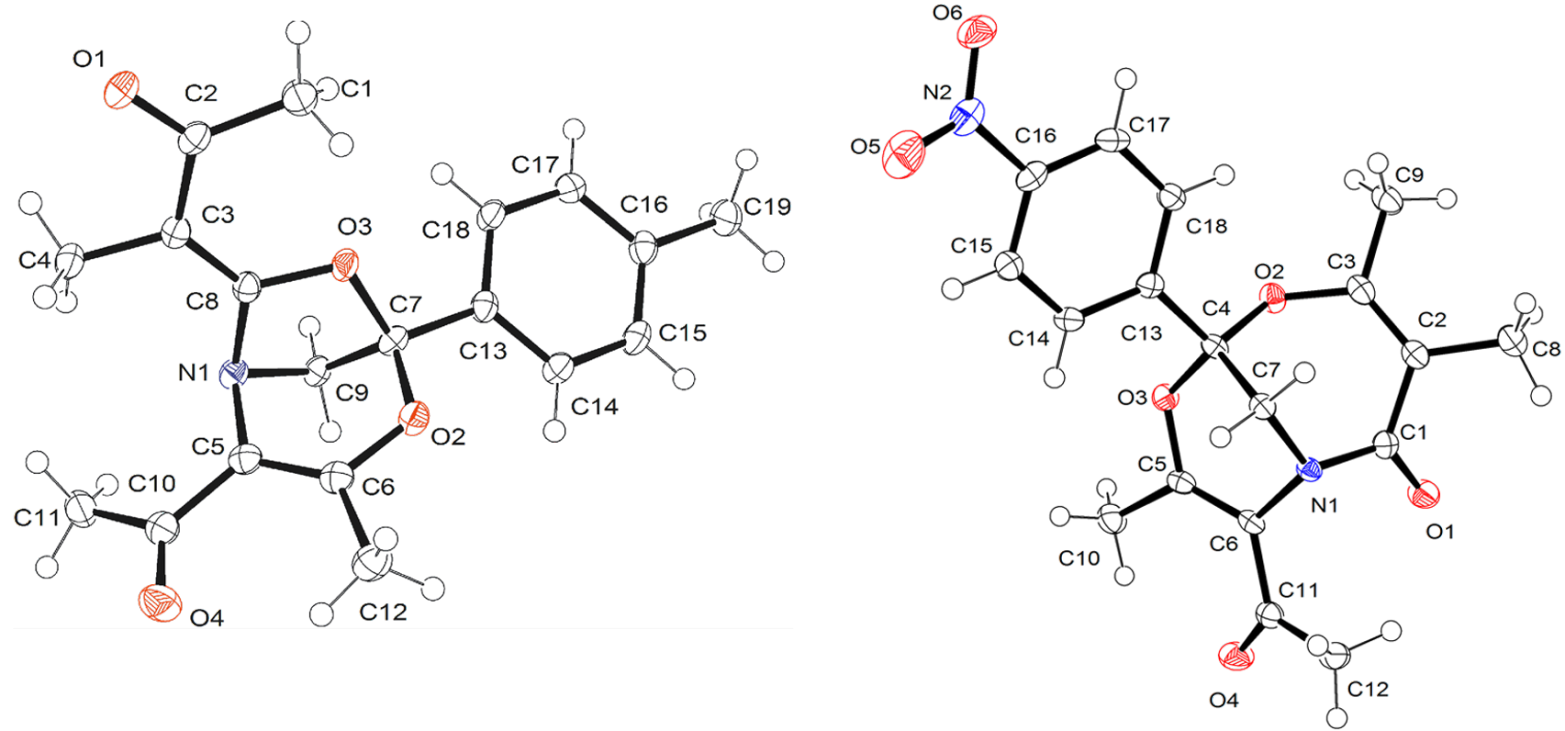
X-ray crystal structures of compounds **6f** and **7h**.

When 3-(*p*-tolyl)azirine **1e** was reacted with diazoacetylacetone **2c** under similar conditions (Rh_2_(OAc)_4_, 60 °C, DCE), four products were detected by ^1^H NMR spectroscopy (Scheme 2, Table 1, entry 6). However, chromatography of the reaction mixture on silica gel gave only oxazine **4f** in 17% yield, whereas other compounds completely decomposed. The use of dirhodium tetraoctanoate Rh_2_(Oct)_4_ as a catalyst in refluxing DCE, as well as an Et_3_N-doped eluent gave a slightly higher yield of **4f** (Table 1, entry 7) and allowed isolation of three byproducts formed via cleavage of the azirine N–C3 bond: bicycle **6f** (hereinafter referred to as the [3.2.1] adduct), bicycle **7f** (hereinafter referred to as the [4.3.1] adduct), and 5,7-dioxa-1-azabicyclo[4.4.1]undeca-3,8-diene derivative **8f**. The same catalyst and the same reaction and purification conditions were used in further experiments. Analogous reaction of 4-methoxyphenyl-substituted azirine **1f** yielded the same set of products (Scheme 2, Table 1, entry 8). Unfortunately, [3.2.1] adduct **6g** and imide **8g** were too unstable to be isolated by chromatography on silica gel, even using Et_3_N-doped eluents. Their presence in the reaction mixture was unambiguously confirmed by ^1^H NMR spectroscopy (Table 1, entry 8). At the same time, oxazine **4g** and adduct **7g** are stable on silica and were isolated in a pure form. The reaction of azirine **1g** containing a 4-nitrophenyl substituent on C3 produces only oxazine **4h** and [4.3.1] adduct **7h**, which were isolated in 58 and 13% yields, respectively (Table 1, entry 9). Compounds **6**–**8** were characterized by standard spectral methods and the structures of adducts **6f** and **7h** were additionally confirmed by X-ray diffraction analysis (Figure 2). The reaction of diazoacetylacetone **2c** with 2,3-diphenyl-2*H*-azirine (**1a**) provides oxazine **4i** as a sole product (Table 1, entry 10). It was isolated with the highest yield of 47% by slow addition of a solution of the diazo compound to a solution of the azirine and Rh_2_(OAc)_4_ in DCE at 60 °С (procedure B).

**Scheme 3:**
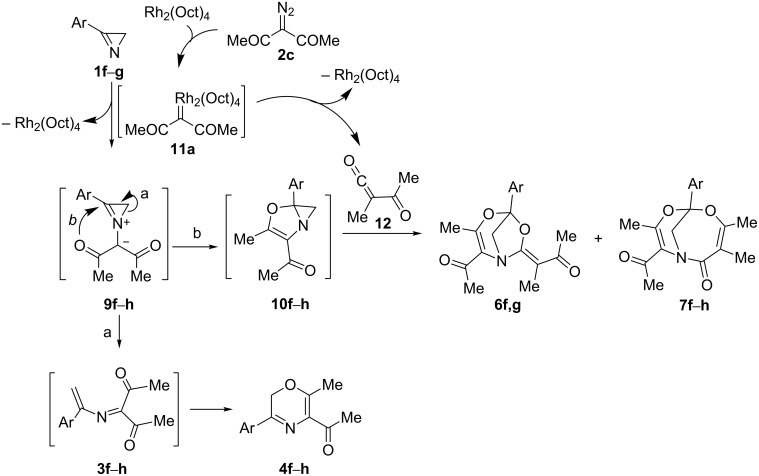
General scheme for the formation of compounds **4**,**6** and **7**.

To the best of our knowledge, neither heterocyclic systems with a 4,6-dioxa-1-azabicyclo[3.2.1]oct-2-ene backbone (compounds **6**) nor systems with a 5,7-dioxa-1-azabicylo[4.4.1]undec-8-ene backbone (compounds **7**) have ever been reported. On the contrary, bicyclic compounds **8f**,**g** are representatives of a known class of 5,7-dioxa-1-azabicylo[4.4.1]undeca-3,8-diene-2,10-dione derivatives formed by the recently reported reaction of 3-arylazirines with acyl ketenes generated by thermolysis of 2-diazo-1,3-diketones [12] or 5-arylfuran-2,3-diones [13]. Therefore, the presence of compounds **8f**,**g** among the reaction products provides evidence for the formation of some amounts of acetyl(methyl)ketene (**12**) under the reaction conditions, which, in turn, gives us insight to the mechanism of formation of adducts **6** and **7** (Scheme 3). A separate experiment was performed to show that these compounds are formed via independent pathways, as they do not interconvert under the reaction conditions (Rh_2_(Oct)_4_, 84 °C, DCE).

We assumed that the reaction sequence leading to bicycles **6** and **7** involves the 1,5-cyclization of azirinium ylide **9f**–**h** to dihydroazireno[2,1-*b*]oxazole **10f**–**h** followed by cycloaddition of the latter to ketene **12** to give two regioisomeric adducts **6f**,**g** and **7f–h** (Scheme 3).

**Scheme 4:**
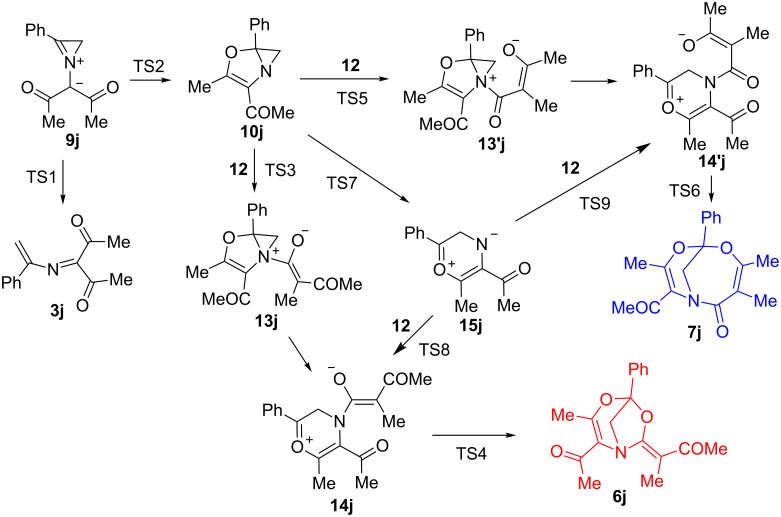
Possible pathways for the formation of **6j** and **7j** from azirenooxazole **10j** and ketene **12**.

Several examples of the 1,5-cyclization of azomethine ylides bearing an α-keto group into oxazole derivatives were reported [27–29]. As also known, the azomethine ylide derived from *N*-benzylideneanisidine and diazoacetylacetone under Rh_2_(OAc)_4_-catalysis undergoes 1,3-cyclization to an aziridine derivative in high yield, rather than 1,5-cyclization [22]. However, no cyclizations of azirinium ylides, cyclic analogs of azomethine ylides, are known. This is not surprising in view of the high strain of the azirinium system, and until now ring opening in these systems seemed much more preferable than annelation of a new cycle. Nevertheless, we decided to study two competing pathways for isomerization of the model azirinium ylide **9j**: ring opening into azadiene **3j** and 1,5-cyclization into azirenooxazole **10j** (Scheme 4), by means of DFT calculations (B3LYP/6-31+G(d,p)). In addition, two reasonable pathways for the formation of adducts **6j** and **7j** formed from azirenooxazole **10j** and ketene **12** were studied at the same level of theory (Scheme 4).

**Figure 3:**
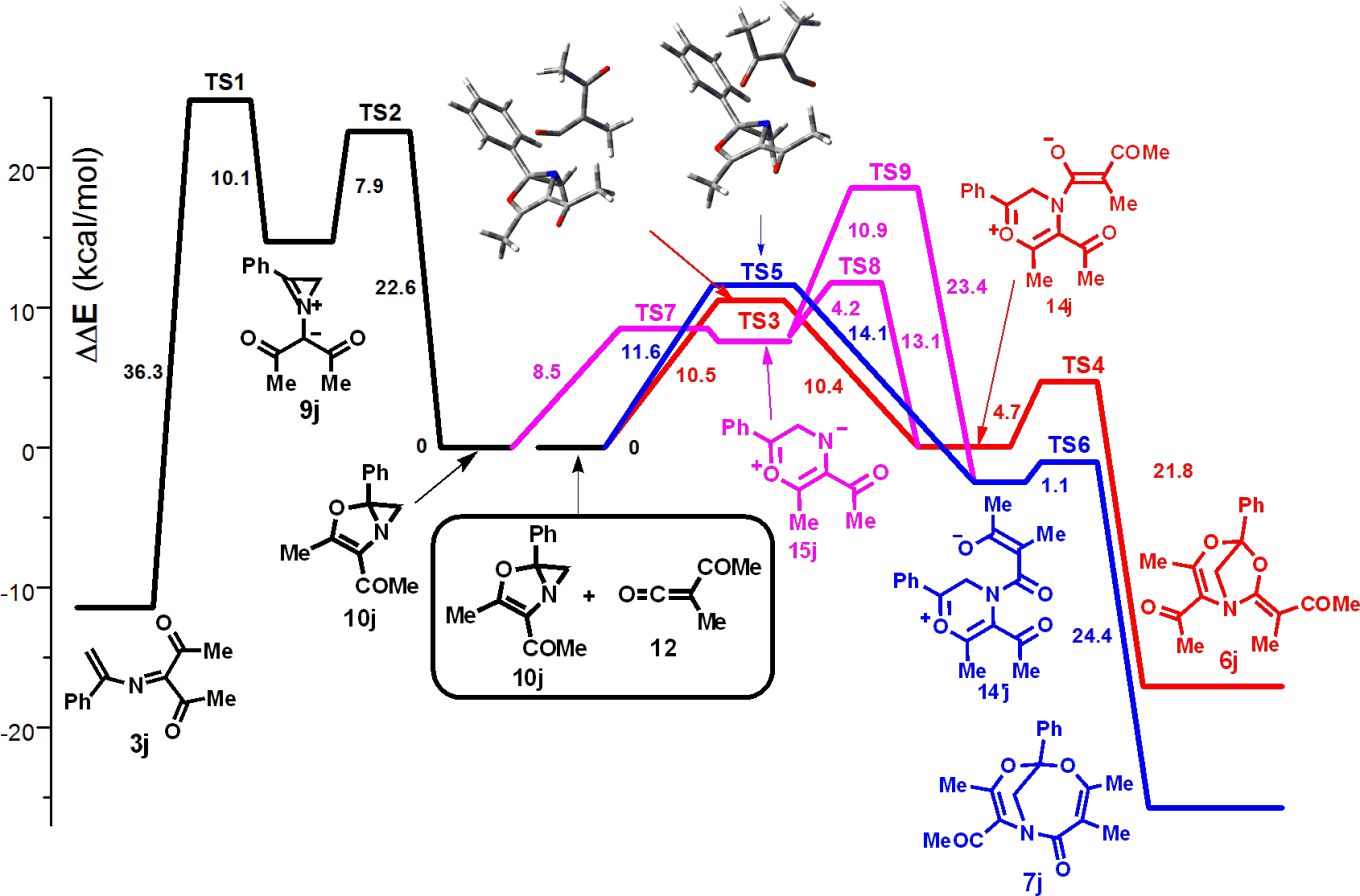
Energy profiles [DFT B3LYP/6-31+G(d,p), 357 K, 1,2-dichloroethane (PCM)] for the transformation of ylide **9j** into azadiene **3j** and dihydroazireno[2,1-*b*]oxazole **10j** and for the transformation of **10j** to adducts **6j**,**7j**.

According to the calculations, the barrier to the 1,5-cyclization of ylide **9j** to compound **10j** was found to be even slightly lower (7.9 kcal/mol) than the barrier to the ring opening across the N–С2 bond to azadiene **3j** (10.1 kcal/mol) (Figure 3). Azirenooxazole **10j** is thermodynamically more stable than ylide **9j**, by ca. 15 kcal/mol, but the barrier to the reverse reaction **10j→9j** is not too high (22.6 kcal/mol). The ring opening in azirinium ylide **9j** into azadiene **3j**, too, has a low activation barrier but occurs irreversibly. Therefore, azirenooxazole **10j** might form in this reaction, and, moreover, its formation from ylide **9j** is kinetically preferred over the formation of azadiene **3j**. However, in view of the reversibility of the 1,5-cyclization **9j** →**10j** and in the absence of an active trap for azirenooxazole **10j** in the reaction mixture, it isomerizes via ylide **9j** to a much more thermodynamically stable open-chain form **3j**.

Curiously, the same energy profile (see Supporting Information File 1, Scheme S1) was obtained for isomerization of azirinium ylide **5** (Scheme 1, R^1^ = Me, R^3^ = R^4^ = H, R^5^ = Ph, Alk = Me) which contains one acyl group and one ester group at the carbanion center. From these results it follows that 1,5-cyclization of azirinium ylides is a general stabilization route for ylides derived from diazo compounds containing an α-keto group. However, azirenooxazoles **10** reversibly formed in these reactions could not be detected in the absence of an active electrophilic trap in the reaction mixture. 3-Diazoacetylacetone under Rh(II)-catalysis gives, along with a Rh(II) carbenoid, a highly electrophilic acetyl(methyl)ketene (**12**) via Wolff rearrangement (Scheme 3).

The nucleophilic attack of the azirenooxazole **10j** nitrogen on the ketene sp-carbon followed by cyclization provides two isomeric adducts **6j** and **7j**. There are two reasonable pathways to these unexpected products. The first pathway involves the addition of bicycle **10j** to ketene **12** to form atropoisomeric azirenooxazolium betains **13j**,**13′j** (Scheme 4) which can further undergo aziridine ring opening to give atropoisomeric oxazinium betaines **14j**,**14′j** and their cyclization into adducts **6j** and **7j**, respectively. Besides, betaines **14j**,**14′j** can result from aziridine ring opening in azirenooxazole **10j** into carbonyl ylide **15j** and its addition to ketene **12** (Scheme 4).

The energy profiles for both the cycloaddition pathways of **10j** to **12** are represented in Figure 3 with the use of relative scale for total energy ΔΔ*E*. According to the calculations, the two attack modes of azirenooxazole **10j** to ketene **12** (red and blue lines on the plot) give rise to atropoisomeric azirenooxazolium betains **13j**,**13′j** (not shown in the plot) which undergo a virtually barrierless aziridine ring opening to give oxazinium betaines **14j**,**14′j**. The activation barriers to both the transformation pathways of **10j** to **13** are close to each other (10.5 and 11.6 kcal/mol, respectively) but much lower than that to the ring opening of azirenooxazole **10j** to azirinium ylide **9j**. The cyclizations of betaines **14j**,**14′j** into bicycles **6j**,**7j** proceed with extremely low activation barriers (1.1 and 4.7 kcal/mol). The alternative pathway to compounds **6j**,**7j,** involving reversible formation of carbonyl ylide **15j,** can result in exclusive formation of [3.2.1] adduct **6j**, because the transition state TS9 leading to betaine **14′j**, a precursor of [4.3.1] adduct **7j**, has a higher energy than the transition state TS5 for the competitive pathway. In spite of the fact that the transition states TS3 and TS8 for alternative betaine **14j** formation pathways are very close in energy, the reaction sequence **10j**→**13j**→**14j** leading to [3.2.1] adduct **6j** seems to be more reasonable than **10j**→**15j**→**14j** due to the much higher concentrations of the reacting species. Actually, the activation barrier to the bicyclic C–N bond cleavage in **10j** is very low, but the equilibrium between **10j** and **15j** is strongly shifted toward bicyclic isomer **10j**, and, therefore, carbonyl ylide **15j** should be formed in an extremely low concentration.

Thus, the computations predict: a) two competing pathways for transformation of azirinium ylides derived both from 2-diazo-1,3-diketons and α-diazo-β-ketoesters; b) a high reactivity of azirenooxazole **10j** toward acetyl(methyl)ketene (**12**); c) two competing modes of the attack of **10j** on ketene **12**, leading to adducts **6j**,**7j** via two short-lived betaine intermediates **13j**,**13′j** and **14j**,**14′j**.

It is worthy to notice that the distribution of products **6**,**7** strongly depends on the electronic effects of the *para* substituents in the aryl group of 3-aryl-2*H*-azirine **1**. The [3.2.1] adduct is preferably formed from azirines **1e,f** with electron-donating substituents (Table 1, entries 7 and 8), while 3-(4-nitrophenyl)-2*H*-azirine (**1g**) provides the [4.3.1] adduct only (Table 1, entry 9).

**Scheme 5:**
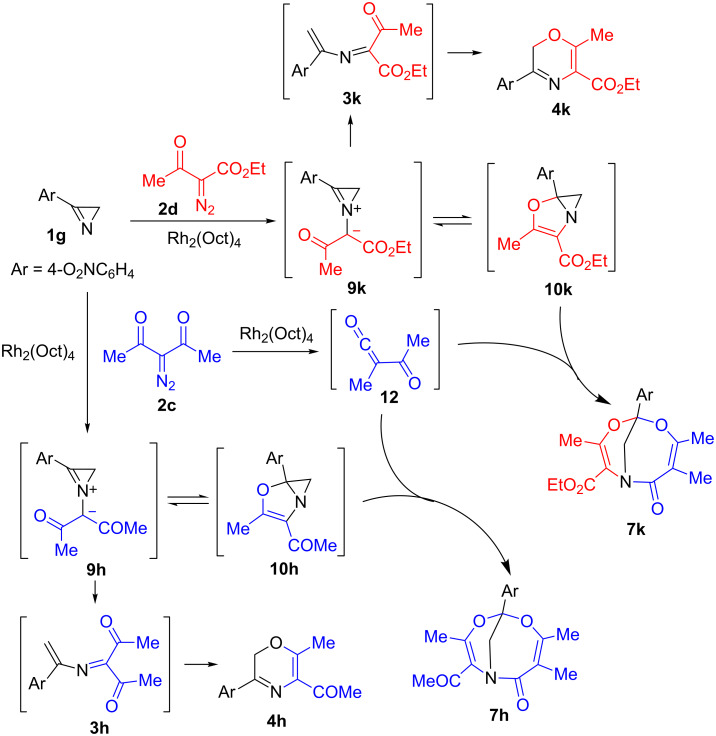
Rh_2_(Oct)_4_-catalyzed reaction of azirine **1g** with diazo compound **2d** in the presence of diazo compound **2c**.

It is known that the Rh(II)-catalyzed reaction of 3-aryl-2*H*-azirines with ethyl diazoacetoacetate (**2d**) gives rise to 2*H*-1,4-oxazines as a single product [15]. The absence of products like **6** or **7** may be caused by a decreased propensity of the carbenoid derived from **2d** to undergo a Wolff rearrangement into a ketene derivative. To obtain experimental evidence for the formation of azirenooxazole intermediates **10** in the reactions of azirines **1** with α-diazo-β-ketoesters, we reacted azirine **1g** with a mixture of two diazo compounds, ethyl diazoacetoacetate (**2d**) and diazoacetylacetone (**2c**), in the presence of Rh_2_(Oct)_4_ (Scheme 5). We suggested that the azirenooxazole formed from the azirine and diazo compound **2d** will be trapped by ketene **12** generated from diazoacetylacetone **2c** via Wolff rearrangement. The ^1^H NMR and TLC analysis of the reaction mixture revealed, along with oxazines **4k**, **4h** and adduct **7h**, one compound. The latter was not detected among the products of the reactions of azirine **1g** separately with each of the diazo compounds. This product was isolated by column chromatography on silica gel, and its structure corresponding to the [4.3.1] adduct **7k** formed by cycloaddition of ketene **12** to azirenoxazole **10k** was assigned on the basis of the ^1^H and ^13^C NMR and mass spectra. According to the ^1^H NMR spectrum of the reaction mixture, the **4k**:**4h**:**7k**:**7h** ratio was 16:7:1:2. In the analogous reaction of 3-(*p*-tolyl)azirine (**1e**) with a mixture of diazo compounds **2c**,**d**, no other products than [3.2.1] adduct **6l** (see Supporting Information File 1, Scheme S1) formed from the ethoxycarbonyl-substituted azirenooxazole derivative and ketene **12** were detected.

**Scheme 6:**
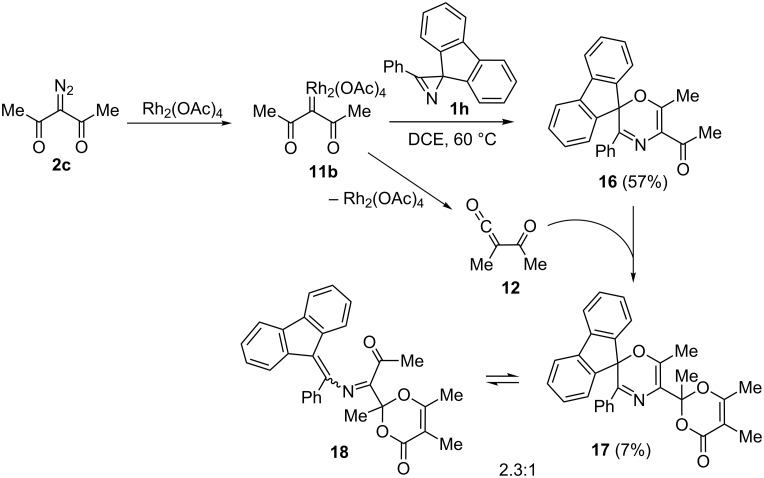
Rh_2_(OAc)_4_-catalyzed reaction of azirine **1h** with diazo compound **2c**.

**Figure 4:**
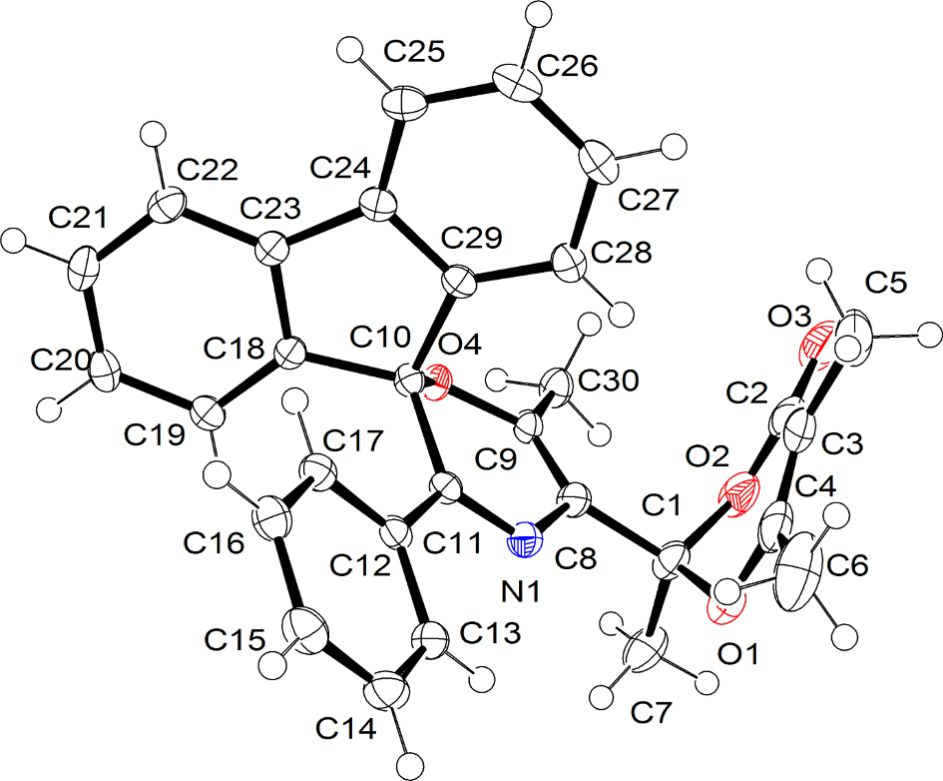
X-ray crystal structure of compound **17**.

Obviously, the bridgehead nitrogen in azirenooxazoles **10** must be sterically accessible for smooth addition of **10** to ketene **12**. For example, methyl or phenyl substitution in the one-atom bridge in the related 5-oxa-1-azabicyclo[4.1.0]hept-3-ene system completely suppresses the reactivity of the latter toward acylketenes [12]. This is a possible reason for the formation of neither **6** nor **7** adduct in the reaction of diazoacetylacetone (**2c**) with 2,3-diphenyl-2*H*-azirine (**1a**, Table 1, entry 10). The moderate yield of compound **4i** is explained by the formation of an unstable by-product of the transformation of the acetyl group in target oxazine **4i** under the reaction conditions. We succeeded in isolating a by-product of this type in the analogous reaction of spiroazirine **1h** with diazo compound **2c** (Scheme 6). Along with oxazine **16** (57%), small amount of 1,4-oxazine **17** with a modified acetyl group at С5 was isolated by column chromatography. Compound **17** obviously resulted from cycloaddition of acyl ketene **12** derived from **2c** under the reaction conditions to the carbonyl group of oxazine **16**. Actually, compound **17** formed in the reaction of a pure oxazine **16** with diazo compound **2c** in the presence of Rh_2_(OAc)_4_. Its structure was assigned by standard spectral methods and confirmed by X-ray diffraction analysis (Figure 4). It was found that, when kept for a week in a CDCl_3_ solution in the dark at room temperature, oxazine **17** undergoes reversible ring opening to form a 1:2.3 equilibrium mixture of **17** and **18**. Azadiene **18** was isolated by chromatography and characterized by standard spectral methods.

**Scheme 7:**
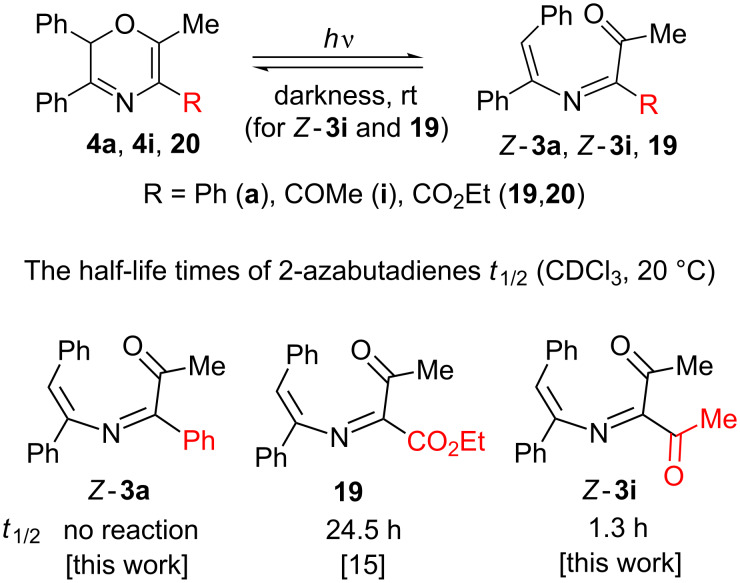
Effect of the C5-substituent in the 2*H*-1,4-oxazine system on its photochromic activity.

The synthesized 5-phenyl- (**4a–d)** and 5-unsubstituted (**4e**) 2*H*-1,4-oxazines proved to be stable in the dark but undergo an irreversible ring opening to azadienes **3** under UV irradiation. Thus, irradiation of oxazine **4a** for 1.5 h gives quantitatively azadiene *Z*-**3a** which was also obtained by the reaction of azirine **1a** and α-diazo-α-phenylacetone (**2a**, Table 1, entry 1). By contrast, 5-acetyl-substituted oxazines **4f–i** are photochromic compounds. Being pale yellow colored, under UV irradiation in a CDCl_3_ solution at room temperature they undergo a ring opening to yellow–orange azadienes **3f–i** which cyclize back to oxazines **4f–i** in the dark. The effect of the C5-substituent in 2*H*-1,4-oxazine on its photochromic activity can be tracked by changes in the half-life times of open-chain isomers *Z*-**3a**,*Z*-**3i** and **19** (Scheme 7). It was found that the azadiene cyclization rate strongly depends on the electron-withdrawing ability of substituent R and increases as it changes from Ph or H to CO_2_Et or COMe.

## Conclusion

5-Unsubstituted and 5-aryl-substituted 2*H*-1,4-oxazines can be readily prepared by the Rh(II)-catalyzed reaction of 2*H*-azirines with α-diazo ketones. The reaction involves intermediate formation of azirinium ylides and their irreversible ring opening across the N–C2 bond to 2-azabuta-1,3-diene followed by 1,6-cyclization. Along with ring opening, transient azirinium ylides more readily undergo reversible 1,5-cyclization to dihydroazireno[2,1-*b*]oxazoles which can be trapped with such active electrophiles as acylketenes to give 4,6-dioxa-1-azabicyclo[3.2.1]oct-2-ene and/or 5,7-dioxa-1-azabicyclo[4.3.1]deca-3,8-diene-2-one derivatives. This reaction sequence partly occurs, when 3-aryl-2*H*-azirines and 3-diazoacetylacetone, which is able to produce acetyl(methyl)ketenes via Rh(II)-catalyzed Wolff rearrangement, are used as starting materials. According to DFT B3LYP/6-31+G(d,p) calculations, the cycloaddition of dihydroazireno[2,1-*b*]oxazoles to acetyl(methyl)ketene, leading to the final bicyclic products, proceeds via consecutive low barrier formation of two pairs of atropoisomeric betaine intermediates followed by their cyclization.

The reaction of 2,3-di- and 2,2,3-triaryl-2*H*-azirines with both α-diazo-α-phenylacetone and 3-diazoacetylacetone gives rise to 2-azabuta-1,3-dienes and/or 2*H*-1,4-oxazines. The alternative reaction pathway via N–C3 azirine bond cleavage does not take place in this case because of the steric shielding of the nitrogen atom in the dihydroazireno[2,1-*b*]oxazole intermediate. Under UV irradiation 5-unsubstituted and 5-aryl-substituted 2*H*-1,4-oxazines produce stable 2-azabuta-1,3-dienes. In contrast, 5-acyl-substituted 2*H*-1,4-oxazines are photochromic compounds, i.e., under UV irradiation they undergo ring opening to 2-azabuta-1,3-dienes which transform back to the oxazines in the dark at room temperature. The rate of the reverse cyclization reaction increases with increasing electron-withdrawing ability of C1-substituent in 2-azabuta-1,3-diene.

## Supporting Information

File 1 Experimental part, computational details and copies of ^1^H and ^13^C NMR spectra.
