# Supplementary material for: Azirinium ylides from α-diazoketones and 2H-azirines on the route to 2H-1,4-oxazines: three-membered ring opening vs 1,5-cyclization
Source: Beilstein J Org Chem. 2015 Mar 2;11:302–12. doi: 10.3762/bjoc.11.35 (PMC4362021; doi:10.3762/bjoc.11.35)
Supplement: File 1 — Experimental part, computational details and copies of 1H and 13C NMR spectra. [file Beilstein_J_Org_Chem-11-302-s001.pdf]

Supporting information

for

Azirinium ylides from α-diazoketones and 2*H*-azirines on the route to 2*H*-1,4-oxazines: three-membered ring opening vs 1,5-cyclization

Nikolai V. Rostovskii[1], Mikhail S. Novikov*[1], Alexander F. Khlebnikov[1], Galina L. Starova[1] and Margarita S. Avdontseva[2]

Address: [1]Institute of Chemistry, Saint-Petersburg State University, Universitetskii pr. 26, 198504 St. Petersburg, Russia and [2]Institute of Earth Science, Saint-Petersburg State University, Universitetskaya nab. 7-9, 199034 St. Petersburg, Russia

Email: Mikhail S. Novikov - m.s.novikov@chem.spbu.ru

*Corresponding author

Experimental part, computational details and copies of ^{1}H and ^{13}C NMR spectra

Table of contents

Experimental part ………………………………………….………………….…..……S2

Scheme S1 ………………………………………..……………………………..…....S14

^{1}H, ^{13}C NMR spectra and thermal ellipsoid plots for the crystal structures …..…..S15

Computational details ……………………………………………………….…….…...S42

Table S1 ……………………………………….…………………………..…....S44

Figure S1 ……………………………………………………………………….....S53

Table S2 ……………………………………………………………….…….…S54

References ………………………….……………………….…….………….S57

Experimental part

General experimental details. Melting points were determined on a hot stage microscope and are uncorrected. ^{1}H (300 MHz) and ^{13}C (75 MHz) NMR spectra were recorded on a Bruker DPX 300 and ^{1}H (400 MHz) and ^{13}C (100 MHz) NMR spectra were recorded on a Bruker AVANCE 400 spectrometer in $CDCl_3$. Chemical shifts (δ) are reported in ppm downfield from tetramethylsilane. Electrospray ionization (ESI) mass spectra were measured on a Bruker MaXis mass spectrometer. Elemental analyses were performed on a Hewlett-Packard 185B CHN-analyser. IR spectra were recorded on a Bruker TENSOR 27 spectrometer for tablets in KBr or solutions in CCl_4. Single crystal X-ray data were collected by means of an Agilent Technologies Supernova Atlas and an Agilent Technologies Excalibur Eos diffractometers. The crystals were kept at 100 K during data collection. The structures have been solved by the direct methods and refined by means of the SHELXL-97 program [1] incorporated in the OLEX2 program package Olex2 [2]. Crystallographic data for the structures **3e** (CCDC 1006655), **6f** (CCDC 1006654), **7h** (CCDC 1007812), **18** (CCDC 1006653) have been deposited with the Cambridge Crystallographic Data Centre. Thin-layer chromatography (TLC) was conducted on aluminum sheets precoated with SiO_2 ALUGRAM SIL G/UV_{254}. Azirines **1a** [3], **1b, 1d** [4], **1c** [5],

1e, **1f** [6], **1g** [7], **1h** [8] and diazo compounds **2a** [9], **2b** [10], **2c** [11], **2d** [12] were prepared by the reported procedures.

General procedures for reactions of azirines with diazo compounds under Rh(II)-catalysis

Method A. A solution of azirine **1** (1 equiv) and diazo compound **2** (1.1–3.5 equiv) in anhydrous DCE (0.33 M solution of azirine) was heated to reflux under argon and then catalyst [$Rh_2(Oct)_4$ (2 mol % on azirine) or $Rh_2(OAc)_4$ (5 mol % on azirine)] was added in one portion. The mixture was stirred under reflux until nitrogen evolution stopped (1–2 min for diazo compound **2a,** 2–5 min for diazo compound **2b,c**). The solvent was removed in vacuo and the products were isolated by column chromatography on silica (eluent petroleum ether/EtOAc or benzene/EtOAc).

Method B. A 2.5 M solution of diazoacetylacetone (**2c**) in anhydrous DCE was added dropwise with a syringe at a rate of 0.8 mL h^{-1} to a stirred 0.7 M solution of azirine **1** and $Rh_2(OAc)_4$ (5 mol % on azirine) in anhydrous DCE at 60 °C under argon. The solvent was removed in vacuo and the products were isolated by column chromatography on silica (eluent petroleum ether/EtOAc or benzene/EtOAc).

1-{(*Z*)-[1,2-Diphenylvinyl]imino}-1-phenylpropane-2-one (*Z*-3a) and **6-methyl-2,3,5-triphenyl-2*H*-1,4-oxazine (4a)**. Oxazine **4a** (196 mg, 60%) and azadiene *Z*-**3a** (23 mg, 7%) were obtained from azirine **1a** (193 mg, 1 mmol) and 1-diazo-1-phenylacetone (**2a**) (200 mg, 1.25 mmol) according to the method **A** ($Rh_2(OAc)_4$ as a catalyst, eluent for chromatography benzene–EtOAc, 400 : 1).

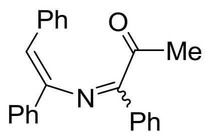

Compound *Z*-**3a** (1.1 : 1 mixture of stereoisomers across C=N bond). Orange oil; ^{1}H NMR (300 MHz, $CDCl_3$) δ 1.92 (s, 3H) *Z*-, 2.84 (s, 3H)

E-, 6.21 (s, 1H) *E*-, 6.40 (s, 1H) *Z*-, 6.75–6.90 (m, 2H) *E*-, 7.03–7.70 (m, 26H), 7.86–8.03 (m, 2H).

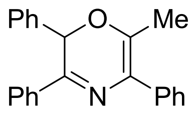

Compound **4a**. Pale-yellow oil; ^{1}H NMR (300 MHz, $CDCl_3$) δ 2.06 (s, 3H), 6.31 (s, 1H), 7.25–7.47 (m, 11H), 7.56 (d, *J* = 7.4 Hz, 2H), 7.95–7.98 (m, 2H); ^{13}C NMR (75 MHz, $CDCl_3$) δ 17.1, 71.2, 126.5, 126.6, 127.7, 127.91, 127.93, 127.96, 128.5, 128.6, 128.8, 130.0, 136.0, 136.3, 137.5, 139.8, 148.9. IR (CCl_4, cm^{-1}) *ν*: 1623 (C=N); HRMS–ESI: $[M + H]^+$ calcd for $C_{23}H_{20}NO^+$, 326.1539; found 326.1541.

1-(6-Methyl-3,5-diphenyl-2*H*-1,4-oxazin-2-yl)-1*H*-benzotriazole (4b).

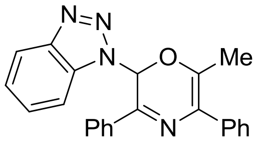

Oxazine **4b** (51 mg, 55%) was obtained from azirine **1b** (59 mg, 0.25 mmol) and 1-diazo-1-phenylacetone **2a** (83 mg, 0.52 mmol) according to the method ***A*** ($Rh_2(OAc)_4$ as a catalyst, eluent for chromatography benzene–EtOAc, 200 : 1). Pale-yellow solid; mp 146–147 °C (Et_2O); ^{1}H NMR (300 MHz, $CDCl_3$) δ 2.07 (s, 3H), 7.34–7.51 (m, 9H), 7.62–7.67 (m, 2H), 7.90–7.95 (m, 3H), 8.10 (d, *J* = 7.1 Hz, 1H). ^{13}C NMR (75 MHz, $CDCl_3$) δ 16.8, 75.7, 110.8, 120.2, 124.5, 126.4, 127.1, 127.3, 128.2, 128.4, 128.6, 128.8, 130.8, 131.5, 134.3, 136.6, 140.2, 142.9, 146.8; IR (KBr, cm^{-1}) *ν*: 1627 (C=N); Anal. calcd for $C_{25}H_{18}N_2O_3$: C, 75.39; H, 4.95; N, 15.29. found: C, 75.51; H, 5.22; N, 15.08.

2,2,6-Trimethyl-3,5-diphenyl-2*H*-1,4-oxazine (4c). Oxazine **4c** (92 mg, 58%) was

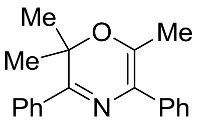

obtained from azirine **1c** (83 mg, 0.57 mmol) and 1-diazo-1-phenylacetone **2a** (102 mg, 0.64 mmol) according to the method ***A*** with subsequent heating under reflux of the mixture for 10 min ($Rh_2(OAc)_4$ as a catalyst,

eluent for chromatography benzene–EtOAc, 200 : 1). Pale-yellow oil; ^{1}H NMR (300 MHz, $CDCl_3$) δ 1.62 (s, 6H), 2.11 (s, 3H), 7.23–7.29 (m, 1H), 7.36–7.43 (m, 5H), 7.61–7.67 (m, 4H); ^{13}C NMR (75 MHz, $CDCl_3$) *δ* 17.2, 24.4 (2C), 73.2, 126.3, 126.8, 127.9, 128.0, 128.0, 128.1, 129.0, 137.5, 137.5, 140.6, 156.1; IR (CCl_4, cm^{-1}) *ν*: 1622 (C=N); HRMS–ESI: $[M + H]^+$ calcd for $C_{19}H_{20}NO^+$, 278.1539; found 278.1543.

1-Phenyl-1-[(triphenylvinyl)imino]propane-2-one (3d) and **6-methyl-2,2,3,5-tetraphenyl-2*H*-1,4-oxazine (4d)**. Oxazine **4d** (15 mg, 7%) and azadiene **3d** (152 mg, 75%) were obtained from azirine **1d** (136 mg, 0.5 mmol) and 1-diazo-1-phenylacetone **2a** (120 mg, 0.75 mmol) according to the method ***A*** ($Rh_2(OAc)_4$ as a catalyst, eluent for chromatography benzene–EtOAc, 400 : 1). Both compounds produce equilibrium **3d**/**4d** mixture in a 4.5 : 1 ratio when standing at room temperature for several days. HRMS–ESI: $[M + H]^+$ calcd for $C_{29}H_{24}NO^+$, 402.1852; found 402.1849.

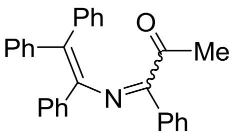

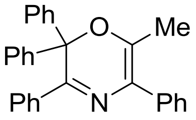

Compound **3d** (1.2:1 mixture of stereoisomers across C=N bond). Orange semi-solid; ^{1}H NMR (300 MHz, $CDCl_3$) δ 1.69 (s, 3H) *Z*-, 2.59 (s, 3H) *E*-, 6.86–7.54 (m, 18H), 7.69 (d, *J* = 7.1 Hz, 2H), *Z*-; ^{13}C NMR (75 MHz, $CDCl_3$) δ 26.4 *E*-, 28.5 *Z*-, 126.5, 126.7, 126.8, 127.0, 127.1, 127.6, 127.7, 128.7 *E*-, 129.2 *Z*-, 129.9 *E*-, 130.6 *E*-, 130.8 *Z*-, 131.2 *Z*-, 131.4 *E*-, 131.8 *Z*-, 133.3 *E*-, 133.9 *Z*-, 138.3 *E*-, 138.8 *Z*-, 141.3, 141.5, 141.9, 143.9 *E*-, 144.9 *Z*-, 165.9 *E*-, 167.1 *Z*-, 200.3 *Z*-, 204.1 *E*-. Compound **4d**. Pale-yellow oil; ^{1}H NMR (300 MHz, $CDCl_3$) δ 2.11 (s, 3H), 7.09–7.54 (m, 20H); ^{13}C NMR (75 MHz, $CDCl_3$) *δ* 16.9, 82.4, 126.4, 127.5, 127.8, 127.8, 127.9, 128.3, 128.6, 128.9, 129.1, 129.2, 137.0, 137.9, 141.0, 141.4, 152.2.

1-(4-Chlorophenyl)-2-[(2-methyl-1-phenylprop-1-enyl)imino]ethanone (3e). Crude

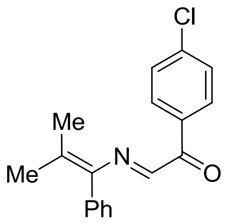

compound **3e** (79 mg, 52%) was obtained from azirine **1e** (73 mg, 0.5 mmol) and 1-diazo-1-phenylpropan-2-one **2b** (271 mg, 1.5 mmol) according to the method ***A*** at 60 °C ($Rh_2(OAc)_4$ as a catalyst, eluent for chromatography hexane–EtOAc, 15 : 1). Trituration of this crude product with cold hexane furnishes 65 mg (43%) of pure **3e** as a pale-yellow solid. Mp 110–117 °C (hexane, dec.); ^{1}H NMR (300 MHz, $CDCl_3$) δ 1.78 (s, 3H), 2.27 (s, 3H), 7.08 (d, J = 6.7 Hz, 2H), 7.33 (s, 1H), 7.36–7.51 (m, 5H), 8.23 (d, J = 8.5 Hz, 2H); ^{13}C NMR (75 MHz, $CDCl_3$) δ 20.1, 22.6, 127.9, 128.3, 128.9, 129.9, 132.1, 134.5, 135.1, 139.1, 144.3, 144.5, 152.1, 190.9; HRMS–ESI: $[M + Na]^+$ calcd for $C_{18}H_{16}ClNNaO^+$, 320.0813; found 320.0813. Crystal data (CCDC-1006655): $C_{18}H_{16}ClNO$, M = 297.77, monoclinic, space group $P$21/n, a = 9.03599(16), b = 15.0636(2), c = 12.0065(2) Å, β = 109.561(2)°, V = 1539.93(5) Å^3, Z = 4, d = 1.284 mg mm^{-3}, F(000) = 624, D_{calc} = 1.284 mg m^{-3}, μ = 2.168 mm^{-1}. 13292 reflections were collected yielding 3028 unique (R_{int} = 0.0258). The final wR_2 = 0.0862 (all data) and R_1 = 0.0335 for 2805 reflections with I ≥ 2σ, GOF = 1.015.

6-(4-Chlorophenyl)-2,2-dimethyl-3-phenyl-2*H*-1,4-oxazine (4e). Azadiene **3e** (60 mg,

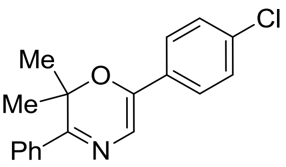

0.2 mmol) in dry DCE (2 mL) was refluxed under argon for 3.5 h to give an equilibrium 6 : 1 mixture of oxazine **4e** and azadiene **3e** (according to ^{1}H NMR spectrum). After refluxing the mixture for additional 1.5 h no change in **3e**/**4e** ratio was observed. The solvent was removed in vacuo, the residue was purified by column chromatography on silica (hexane–EtOAc, 15 : 1) to give 9 mg (15%) of recovered **3e** and 40 mg (67%) of **4e** as a pale-yellow solid. Mp 92–93 °C (hexane). ^{1}H NMR (300 MHz, $CDCl_3$) δ 1.66 (s, 6H), 7.20 (s, 1H), 7.32–7.49 (m, 5H), 7.54–7.68 (m, 4H). ^{13}C NMR (75 MHz, $CDCl_3$) δ 24.3, 74.3, 114.8, 126.3,

127.8, 128.1, 128.6, 129.4, 131.4, 134.8, 137.2, 142.0, 159.8; HRMS–ESI: $[M + H]^+$ calcd for $C_{18}H_{17}ClNO^+$, 298.0993; found 298.1006.

1-[6-Methyl-3-(4-methylphenyl)-2*H*-1,4-oxazin-5-yl]ethanone (4f), (*Z*)-3-[2-acetyl-3-methyl-5-(4-methylphenyl)-4,6-dioxa-1-azabicyclo[3.2.1]oct-2-en-7-yliden]butane-2-one (6f), 9-acetyl-3,4,8-trimethyl-6-(4-methylphenyl)-5,7-dioxa-1-azabicyclo[4.3.1]deca-3,8-diene-2-one (7f) and **6-(4-methylphenyl)-3,4,8,9-tetramethyl-5,7-dioxa-1-azabicylo[4.4.1]undeca-3,8-diene-2,10-dione (8f)**. Compounds **4f** (25 mg, 22%), **6f** (38 mg, 23%), **7f** (12 mg, 7%) and **8f** (7 mg, 4%) were obtained from azirine **1e** (66 mg, 0.5 mmol) and diazoacetylacetone **2c** (221 mg, 1.75 mmol) according to the method ***A*** ($Rh_2(Oct)_4$ as a catalyst, eluent for chromatography petroleum ether–EtOAc–Et_3N, gradient from 75 : 25 : 1 to 50 : 50 : 1).

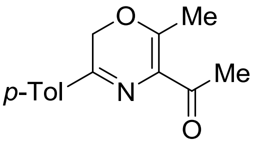

Compound **4f**. Colorless solid; ^1H NMR (300 MHz, $CDCl_3$) δ 2.37 (s, 3H), 2.42 (s, 3H), 2.54 (s, 3H), 4.78 (s, 2H), 7.28 (d, *J* = 8.2 Hz, 2H), 7.76 (d, *J* = 8.2 Hz, 2H); ^{13}C NMR (75 MHz, $CDCl_3$) δ 17.9, 21.4, 28.0, 61.9, 126.2, 128.1, 129.5, 132.3, 140.8, 146.8, 156.4, 199.1; HRMS–ESI: $[M + Na]^+$ calcd for $C_{14}H_{15}NNaO_2^+$, 252.0995; found 252.0999.

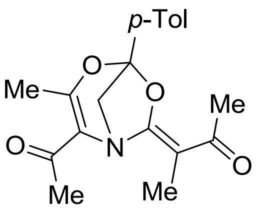

Compound **6f**. Colorless solid; Mp 115–125 °C (Et_2O, dec.); ^1H NMR (300 MHz, $CDCl_3$) δ 1.88 (s, 3H), 2.28 (s, 3H), 2.43 (s, 3H), 2.47 (s, 3H), 2.51 (s, 3H), 3.34 and 3.50 (AB-q, *J* = 11.5 Hz, 2H), 7.31 (d, *J* = 8.2 Hz, 2H), 7.51 (d, *J* = 8.2 Hz, 2H). ^{13}C NMR (75 MHz, $CDCl_3$) δ 13.2, 18.3, 21.3, 28.3, 31.8, 56.8, 101.5, 104.7, 125.7, 126.4, 129.5, 129.9, 140.4, 159.6, 167.2, 196.3, 198.2; HRMS–ESI: $[M + H]^+$ calcd for $C_{19}H_{22}NO_4^+$, 328.1543; found 328.1529; Crystal data (CCDC-1006654): $C_{19}H_{21}NO_4$, *M* = 327.37, monoclinic, space group *P*21/n, *a* = 7.7750(6), *b* = 11.9067(4), *c* = 17.8823(14) Å, β = 99.397(7) °, *V* =

1633.23(19) Å^3, Z = 4, d = 1.331 mg mm^{-3}, F(000) = 696, D_{calc} = 1.331 mg m^{-3}, μ = 0.692 mm^{-1}. 12560 reflections were collected yielding 3244 unique (R_{int} = 0.0218). The final wR_2 = 0.0904 (all data) and R_1 = 0.0350 for 2890 reflections with I ≥ 2σ, GOF = 1.063.

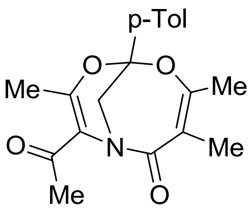

Compound **7f**. Colorless oil; ^{1}H NMR (400 MHz, $CDCl_3$) δ 1.97 (d, J = 0.8 Hz, 3H), 2.02 (d, J = 0.8 Hz, 3H), 2.40 (s, 6H), 2.42 (s, 3H), 3.26 (d, J = 14.8 Hz, 1H), 4.46 (d, J = 14.8 Hz, 1H), 7.26 and 7.35 (AB-q, J = 8.4 Hz, 4H); ^{13}C NMR (100 MHz, $CDCl_3$) δ 14.5, 17.8, 19.4, 21.2, 28.5, 49.8, 107.9, 113.3, 120.8, 125.0, 129.3, 135.5, 139.7, 152.5, 154.8, 172.0, 196.1; HRMS–ESI: [M + H]$^+$ calcd for $C_{19}H_{22}NO_4^+$, 328.1543; found 328.1539.

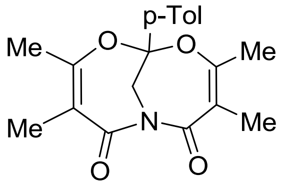

Compound **8f**. Colorless solid; mp 150–165 °C (Et_2O, dec.); ^{1}H NMR (400 MHz, $CDCl_3$) δ 1.91 (d, J = 0.8 Hz, 6H), 1.95 (d, J = 0.8 Hz, 6H), 2.41 (s, 3H), 4.36 (s, 2H), 7.26 (d, J = 8.0 Hz, 2H), 7.43 (d, J = 8.0 Hz, 2H); ^{13}C NMR (100 MHz, $CDCl_3$) δ 15.1, 21.0 (2C), 21.2 48.0, 111.9, 113.1, 124.7, 129.4, 135.8, 139.9, 157.0, 167.2; HRMS–ESI: [M + H]$^+$ calcd for $C_{19}H_{22}NO_4^+$, 328.1543; found 328.1540.

1-[6-Methyl-3-(4-methoxyphenyl)-2*H*-1,4-oxazin-5-yl]ethanone (4g) and **9-acetyl-3,4,8-trimethyl-6-(4-methoxyphenyl)-5,7-dioxa-1-azabicyclo[4.3.1]deca-3,8-diene-2-one (7g)**. Oxazine **4g** (22 mg, 18%) and adduct **7g** (10 mg, 6%) were obtained from azirine **1f** (74 mg, 0.5 mmol) and diazoacetylacetone **2c** (221 mg, 1.75 mmol) according to the method ***A*** ($Rh_2(Oct)_4$ as a catalyst, eluent for chromatography petroleum ether–EtOAc, gradient from 5 : 1 to 2 : 1).

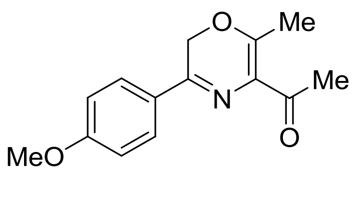

Compound **4g**. Colorless oil; ^{1}H NMR (400 MHz, $CDCl_3$) δ 2.37 (s, 3H), 2.54 (s, 3H), 3.89 (s, 3H), 4.78 (s, 2H), 6.99 (d,

J = 8.8 Hz, 2H), 7.83 (d, *J* = 8.8 Hz, 2H); ^{13}C NMR (100 MHz, $CDCl_3$) δ 17.9, 28.0, 55.4, 61.8, 114.2, 127.8, 127.9, 128.1, 146.5, 156.0, 161.5, 199.2; HRMS–ESI: $[M + H]^+$ calcd for $C_{14}H_{16}NO_3^+$, 246.1125; found 246.1121.

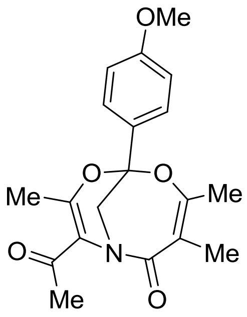

Compound **7g**. Colorless oil; ^{1}H NMR (400 MHz, $CDCl_3$) δ 1.97 (s, 3H), 2.02 (s, 3H), 2.40 (s, 3H), 2.42 (s, 3H), 3.27 (d, *J* = 14.7 Hz, 1H), 3.86 (s, 3H), 4.45 (d, *J* = 14.7 Hz, 1H), 6.96 (d, *J* = 8.8 Hz, 2H), 7.40 (d, *J* = 8.8 Hz, 2H); ^{13}C NMR (100 MHz, $CDCl_3$) δ 14.5, 17.8, 19.3, 28.5, 49.8, 55.4, 107.9, 113.4, 114.0, 120.8, 126.5, 130.6, 152.5, 154.8, 160.5, 172.0, 196.1; HRMS–ESI: $[M + H]^+$ calcd for $C_{19}H_{22}NO_5^+$, 344.1492; found 344.1496.

1-[6-Methyl-3-(4-nitrophenyl)-2*H*-1,4-oxazin-5-yl]ethanone (4h) and **9-acetyl-3,4,8-trimethyl-6-(4-nitrophenyl)-5,7-dioxa-1-azabicyclo[4.3.1]deca-3,8-diene-2-one (7h)**. Oxazine **4h** (75 mg, 58%) and adduct **7h** (23 mg, 13%) were obtained from azirine **1g** (81 mg, 0.5 mmol) and diazoacetylacetone **2c** (221 mg, 1.75 mmol) according to the method ***A*** ($Rh_2(Oct)_4$ as a catalyst, eluent for chromatography petroleum ether–EtOAc, gradient from 5 : 1 to 2 : 1).

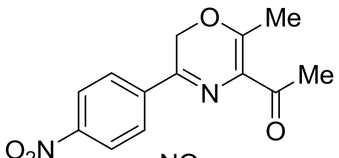

Compound **4h**. Pale-yellow needles; mp 148–150 °C (Et_2O); ^{1}H NMR (400 MHz, $CDCl_3$) δ 2.40 (s, 3H), 2.54 (s, 3H), 4.86 (s, 2H), 8.03 (d, *J* = 8.8 Hz, 2H), 8.31 (d, *J* = 8.8 Hz, 2H); ^{13}C NMR (100 MHz, $CDCl_3$) δ 18.0, 28.0, 61.7, 124.0, 127.1, 128.6, 140.5, 143.9, 148.6, 157.9, 198.2. HRMS–ESI: $[M + H]^+$ calcd for $C_{13}H_{13}N_2O_4^+$ 261.0870; found 261.0867.

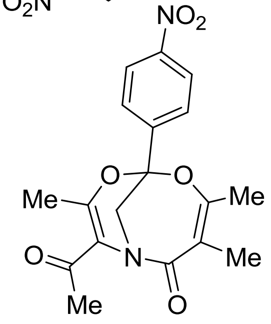

Compound **7h**. Colorless solid; mp 208–209 °C (Et_2O); ^{1}H NMR (400 MHz, $CDCl_3$) δ 1.99 (s, 3H), 2.03 (s, 3H), 2.41 (s, 3H), 2.42 (s, 3H), 3.30 (d, *J* = 14.7 Hz, 1H), 4.54 (d, *J* = 14.7 Hz, 1H), 7.68 (d, *J* = 8.8 Hz, 2H), 8.32 (d, *J* = 8.8 Hz, 2H); ^{13}C NMR (100 MHz, $CDCl_3$) δ

14.6, 17.6, 19.4, 28.5, 49.1, 106.4, 113.8, 121.0, 124.0, 126.3, 144.7, 148.6, 152.1, 154.0 (2C), 171.6, 195.8; HRMS–ESI: $[M + H]^+$ calcd for $C_{18}H_{19}N_2O_6^+$, 359.1238; found 359.1240; Crystal data (CCDC-1007812): $C_{18}H_{18}N_2O_6$, M = 358.34, monoclinic, space group $P$21/n, a = 8.1140(5), b = 9.0367(4), c = 23.1559(12) Å, β = 97.008(6)°, V = 1685.20(16) $Å^3$, Z = 4, d = 1.412 mg mm^{-3}, F(000) = 752, D_{calc} = 1.412 mg m^{-3}, μ = 0.107 mm^{-1}. 7656 reflections were collected yielding 3876 unique (R_{int} = 0.0419). The final wR_2 = 0.1133 (all data) and R_1 = 0.0557 for 2415 reflections with I ≥ 2σ, GOF = 1.017.

1-(6-Methyl-2,3-diphenyl-2*H*-1,4-oxazin-5-yl)ethanone (4i). Oxazine **4i** (73 mg, 47%, 53% based on recovery of azirine) was obtained from azirine **1a** (96 mg, 0.5 mmol) and diazoacetylacetone **2c** (255 mg, 2.02 mmol) according to the method ***B*** ($Rh_2(OAc)_4$ as a catalyst, eluent for chromatography petroleum ether–EtOAc, 8 : 1). Pale-yellow oil; ^1H NMR (400 MHz, $CDCl_3$) δ 2.33 (s, 3H), 2.61 (s, 3H), 6.33 (s, 1H), 7.31–7.38 (m 5H), 7.41–7.47 (m, 3H), 7.85–7.95 (m, 2H); ^{13}C NMR (100 MHz, $CDCl_3$) δ 18.5, 28.1, 72.3, 126.4, 127.1, 127.7, 128.6, 128.8, 129.4, 130.3, 134.9, 135.5, 147.9, 154.4, 198.6; IR (CCl_4, cm^{-1}) ν: 1682 (C=O), 1559 (C=N); HRMS–ESI: $[M + Na]^+$ calcd for $C_{19}H_{17}NNaO_2^+$, 314.1151; found 314.1150.

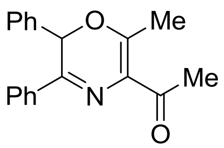

Ethyl 3,4,8-trimethyl-6-(4-nitrophenyl)-2-oxo-5,7-dioxa-1-azabicyclo[4.3.1]deca-3,8-diene-9-carboxylate (7k). Adduct **7k** (8 mg, 2%) was obtained from azirine **1g** (162 mg, 1 mmol), diazoacetylacetone **2c** (150 mg, 1.19 mmol) and ethyl diazoacetoacetate **2d** (280 mg, 1.79 mmol) according to the method ***A*** by use of $Rh_2(Oct)_4$ as a catalyst and

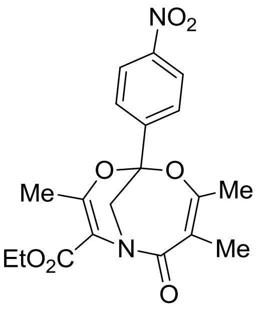

double chromatographic purification (petroleum ether–EtOAc, 2 : 1 and benzene–EtOAc, 10 : 1). ^{1}H NMR (400 MHz, $CDCl_3$) δ 1.38 (t, *J* = 7.1 Hz, 3H), 1.98 (s, 3H), 2.01 (s, 3H), 2.43 (s, 3H), 3.27 (d, *J* = 14.7 Hz, 1H), 4.37 (m, 2H), 4.53 (d, *J* = 14.7 Hz, 1H), 7.68 (d, *J* = 8.9 Hz, 2H), 8.32 (d, *J* = 8.9 Hz, 2H); ^{13}C NMR (100 MHz, $CDCl_3$) δ 14.3, 14.5, 17.5, 19.2, 49.3, 61.1, 106.7, 114.2, 114.4, 124.0, 126.4, 144.9, 148.6, 151.4, 154.9, 163.9, 171.1; HRMS–ESI: $[M + Na]^+$ calcd for $C_{19}H_{20}N_2NaO_7^+$, 411.1163; found 411.1173.

1-(6′-Methyl-3′-phenylspiro[fluorene-9,2′-[1,4]oxazine]-5-yl)ethanone (16) and **2,5,6-trimethyl-2- (6′-methyl-3′-phenylspiro[fluorene-9,2′-[1,4]oxazine]-5′-yl)-4*H*-1,3-dioxin-4-one (17)**. Oxazine **16** (104 mg, 57%, 61% based on recovery of azirine) and oxazine **17** (17 mg, 7%) were obtained from azirine **1h** (134 mg, 0.5 mmol) and diazoacetylacetone **2c** (255 mg, 2.02 mmol) according to the method ***B*** ($Rh_2(OAc)_4$ as a catalyst) by use of chromatographic purification (petroleum ether–EtOAc, 8 : 1) for compound **16** and additional chromatographic purification (benzene–EtOAc, 100 : 1) for compound **17**.

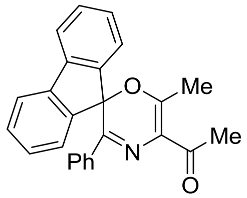

Compound **16**. Colorless solid; mp 169–184 °C (Et_2O, dec.); ^{1}H NMR (300 MHz, $CDCl_3$) δ 2.38 (s, 3H), 2.69 (s, 3H), 7.05–7.13 (m, 2H), 7.16–7.28 (m, 5H), 7.36–7.52 (m, 4H), 7.36–7.52 (d, *J* = 7.4 Hz, 2H); ^{13}C NMR (75 MHz, $CDCl_3$) δ 18.7, 28.3, 83.2, 120.6, 125.7, 126.5, 127.4, 127.7, 128.7, 129.6, 130.6, 135.5, 140.0, 144.6, 150.7, 157.0, 198.8; IR (KBr, cm^{-1}) *ν*: 1671 (C=O), 1568 (C=N); Anal. calcd for $C_{25}H_{19}NO_2$: C, 82.17; H, 5.24; N, 3.83; found: C, 82.18; H, 5.20; N, 4.04.

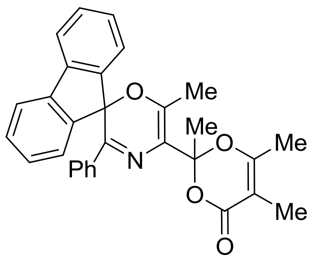

Compound **17**. Colorless solid; mp 134–137 °C (Et_2O, dec.); ^1H NMR (300 MHz, $CDCl_3$) δ 1.98 (s, 3H), 2.04 (s, 3H), 2.08 (s, 3H), 2.11 (s, 3H), 6.99–7.08 (m, 2H), 7.11–7.23 (m, 5H), 7.31–7.51 (m, 4H), 7.69–7.78 (m, 2H); ^{13}C NMR (75 MHz, $CDCl_3$) δ 10.6, 17.0, 17.3, 26.0, 82.3, 102.4, 104.7, 120.4, 120.7, 124.0, 125.2, 125.9, 127.5, 127.6, 128.5, 128.6, 129.3, 130.1, 130.4, 135.6, 139.8, 140.2, 144.5, 145.7, 146.3, 151.4, 162.9, 164.1; HRMS–ESI: $[M + Na]^+$ calcd for $C_{30}H_{25}NNaO_4^+$, 486.1676; found 486.1665; Crystal data (CCDC-1006653): $C_{30}H_{25}NO_4$, M = 463.51, triclinic, space group P-1, a = 7.5692(3), b = 8.5518(3), c = 18.8070(5) Å, α = 79.225(3), β = 87.061(3), γ = 80.555(3), V = 1179.44(7) Å^3, Z = 2, d = 1.305 mg mm^{-3}, F(000) = 488, D_{calc} = 1.305 mg m^{-3}, μ = 0.087 mm^{-1}. 11744 reflections were collected yielding 5376 unique (R_{int} = 0.0285). The final wR_2 = 0.1216 (all data) and R_1 = 0.0479 for 4303 reflections with I ≥ 2σ, GOF = 1.024.

2-{1-[(Fluorene-9-yliden)(phenyl)methylimino]-2-oxopropyl}-2,5,6-trimethyl-4*H*-1,3-dioxin-4-one (18). Oxazine **17** (17 mg, 0.037 mmol) in dry $CDCl_3$ (0.5 mL) was kept at room temperature in darkness for a week to give an equilibrium mixture of oxazine **17** and azadiene **18** in a 1 : 2.3 ratio (according to ^1H NMR spectrum).

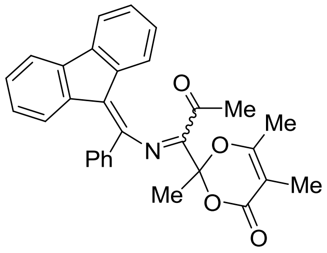

The solvent was removed in vacuo, the residue was purified by column chromatography on silica (petroleum ether–EtOAc, 8 : 1) to give recovered oxazine **17** (5 mg, 30%) and azadiene **18** (12 mg, 70%), which at room temperature slowly transforms back to oxazine **17**. Compound **18**. Orange solid; ^1H NMR (300 MHz, $CDCl_3$) δ 1.77 (s, 3H), 1.92 (s, 3H), 1.97 (s, 6H), 6.61 (d, J = 8.2 Hz, 1H), 6.92 (t, J = 7.6 Hz, 1H), 7.24 (t, J = 7.6, 1H, Hz), 7.30–7.55 (m, 7H), 7.71 (d, J = 7.8 Hz, 1H), 7.77 (d, J = 6.7 Hz, 2H); ^{13}C NMR (75 MHz, $CDCl_3$) δ 10.4, 17.3, 24.3, 29.4, 29.7, 102.9, 103.4, 119.5, 119.6, 122.2,

123.8, 125.9, 126.4, 126.9, 127.3, 127.6, 128.9, 129.5, 130.0, 137.2, 137.3, 137.4, 139.7, 140.1, 145.2, 161.0, 164.2, 167.8, 200.7; HRMS–ESI: $[M + Na]^+$ calcd for $C_{30}H_{25}NNaO_4^+$, 486.1676; found 486.1681.

$Rh_2(Oct)_4$-catalyzed reaction of azirine 1e with the mixture of diazo compounds 2d and 2c.

A solution of azirine **1e** (61 mg, 0.5 mmol), diazo compound **2d** (137 mg, 0.88 mmol) and diazo compound **2c** (81 mg, 0.64 mmol) in anhydrous DCE (3 mL) was heated to reflux under argon and then catalyst ($Rh_2(Oct)_4$, 2 mol% on azirine) was added in one portion. The mixture was stirred under reflux for 5 min until nitrogen evolution stopped and was analyzed by ^1H NMR. The products ratio is represented on the Scheme S-1.

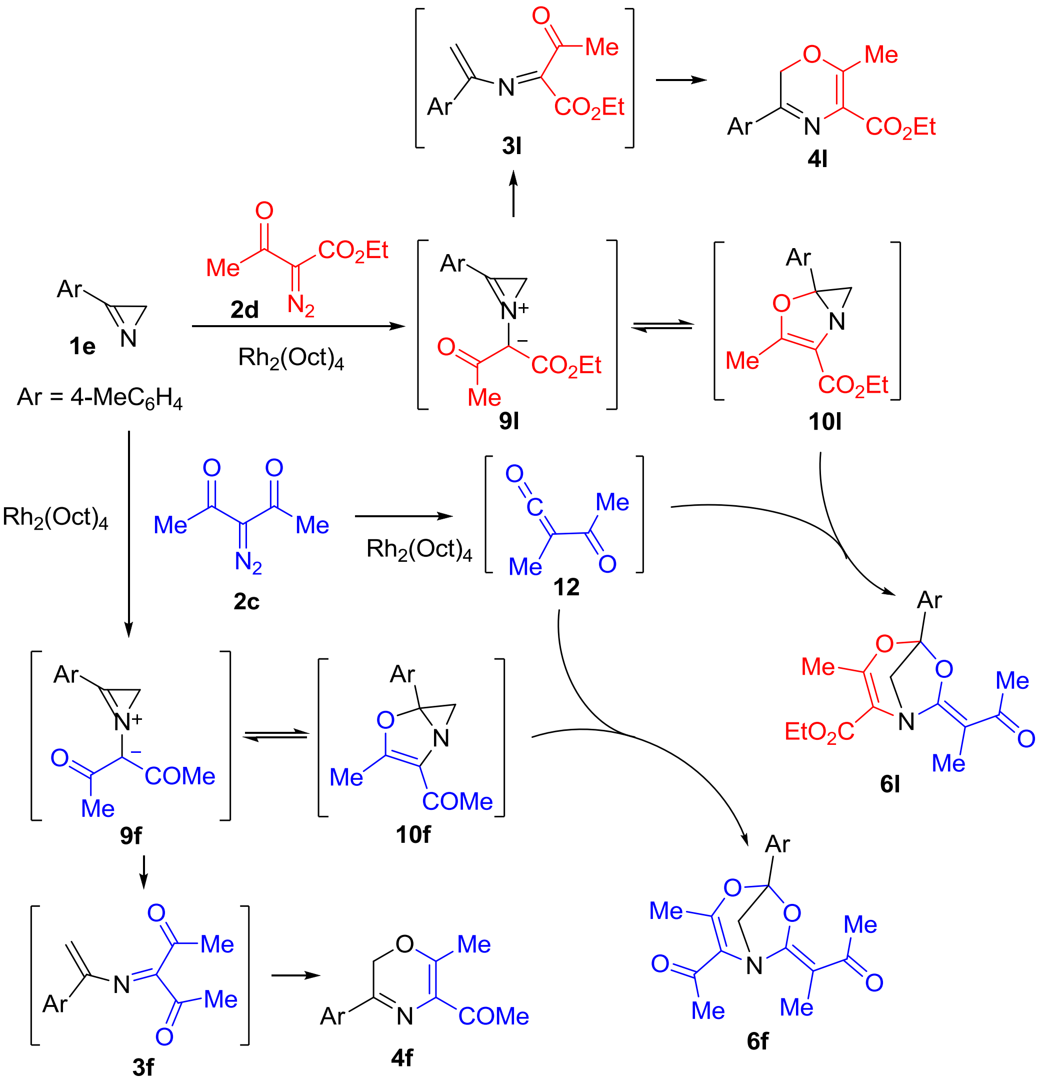

According to ^{1}H NMR
4l : **4f** : **6l** : **6f** = 10 :1 : 5 : 6

Scheme S1: $Rh_2(Oct)_4$-catalyzed reaction of azirine **1e** with diazo compound **2d** in the presence of **2c**.

^{1}H and ^{13}C NMR spectra of new compounds

1-{(*Z*)-[1,2-Diphenylvinyl]imino}-1-phenylpropane-2-one (*Z*-3a)

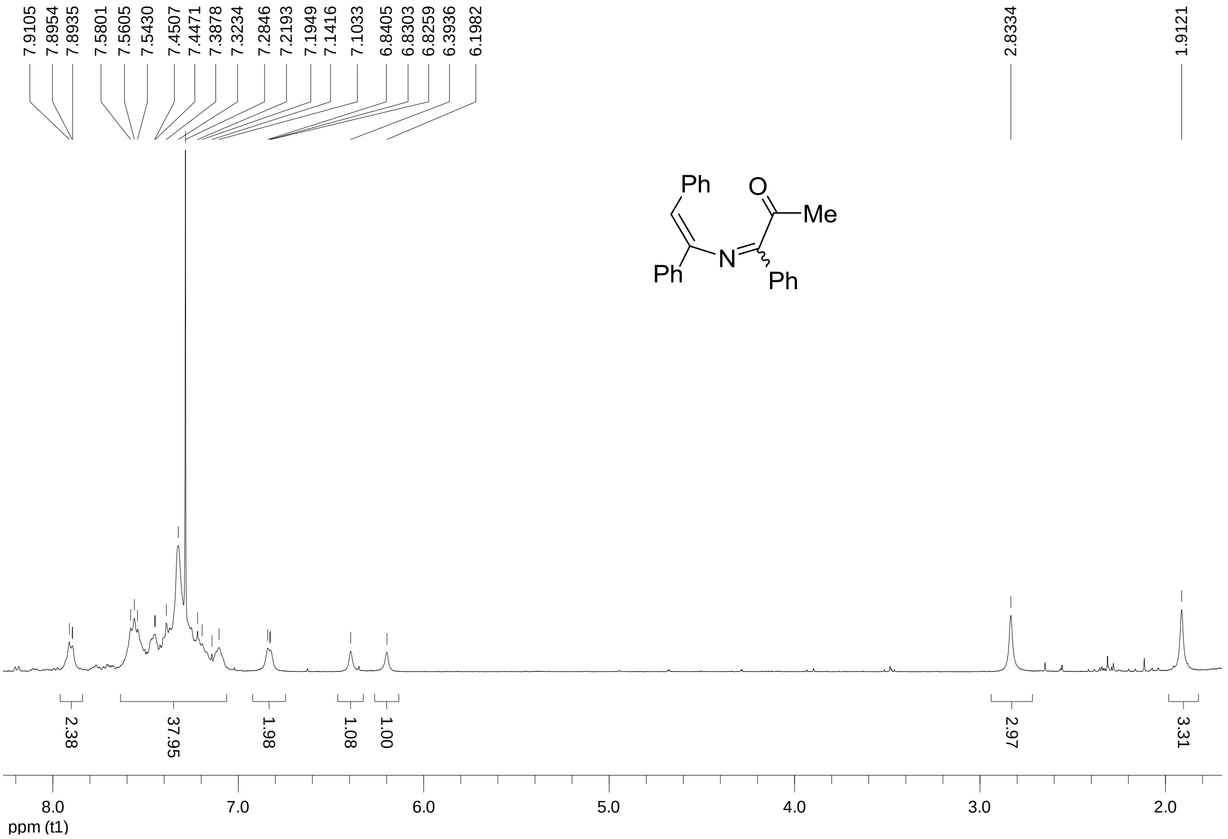

6-Methyl-2,3,5-triphenyl-2*H*-1,4-oxazine (4a)

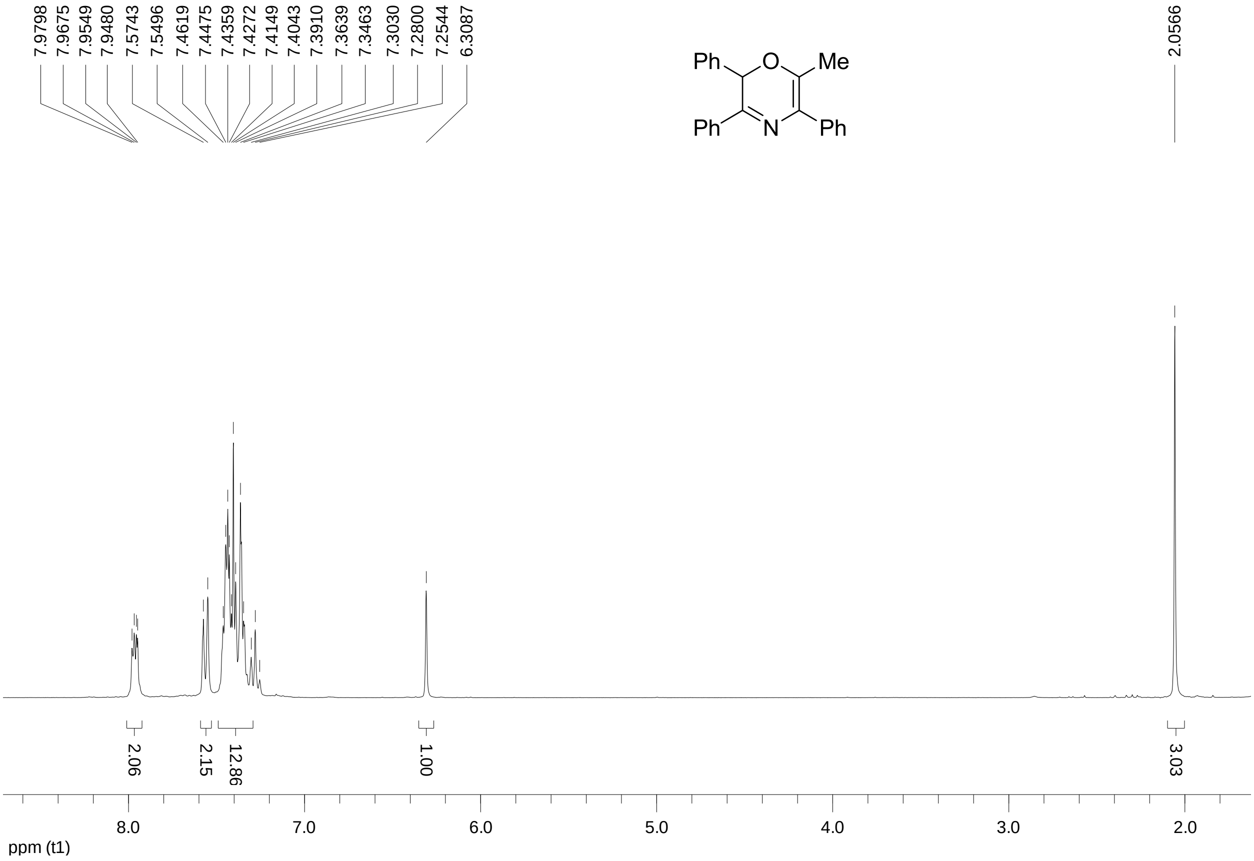

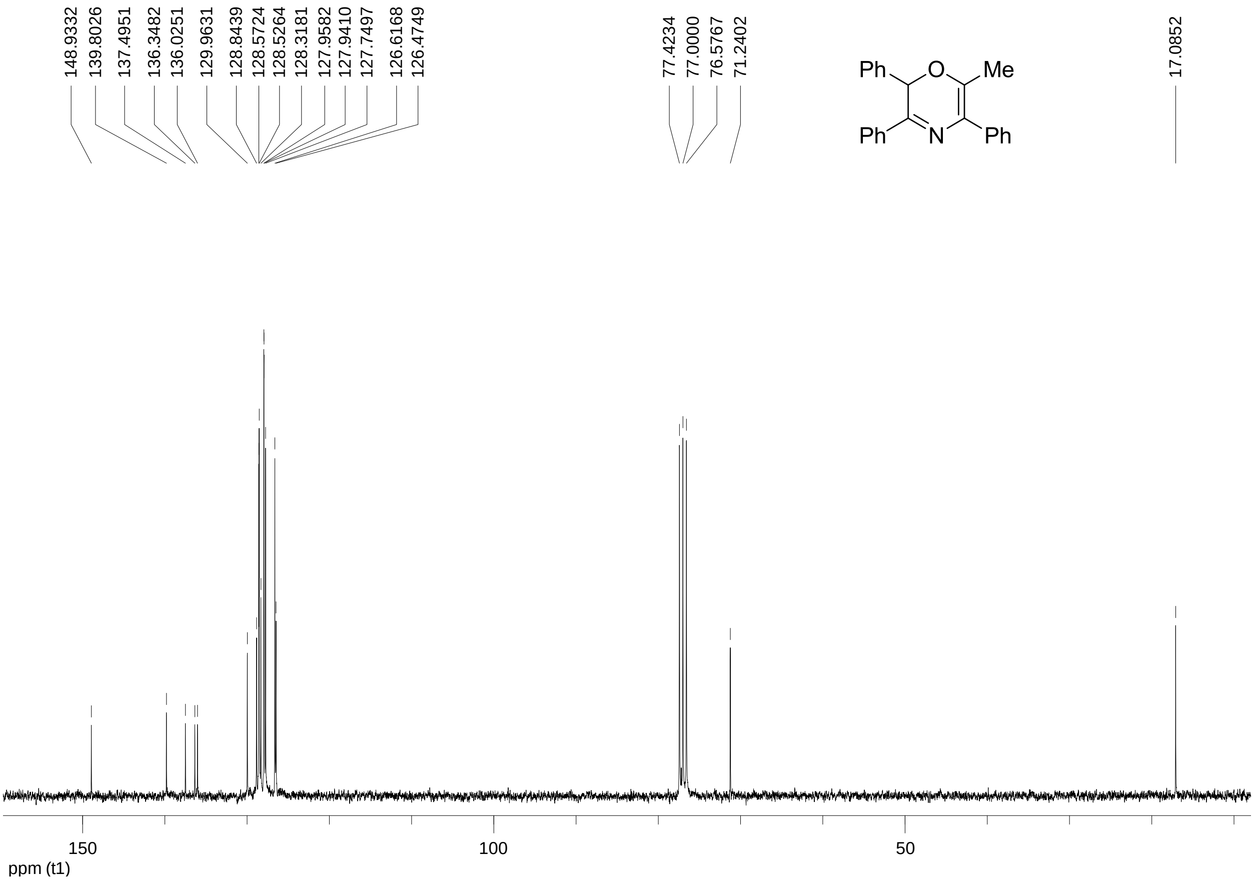

1-(6-Methyl-3,5-diphenyl-2*H*-1,4-oxazin-2-yl)-1*H*-benzotriazole (4b)

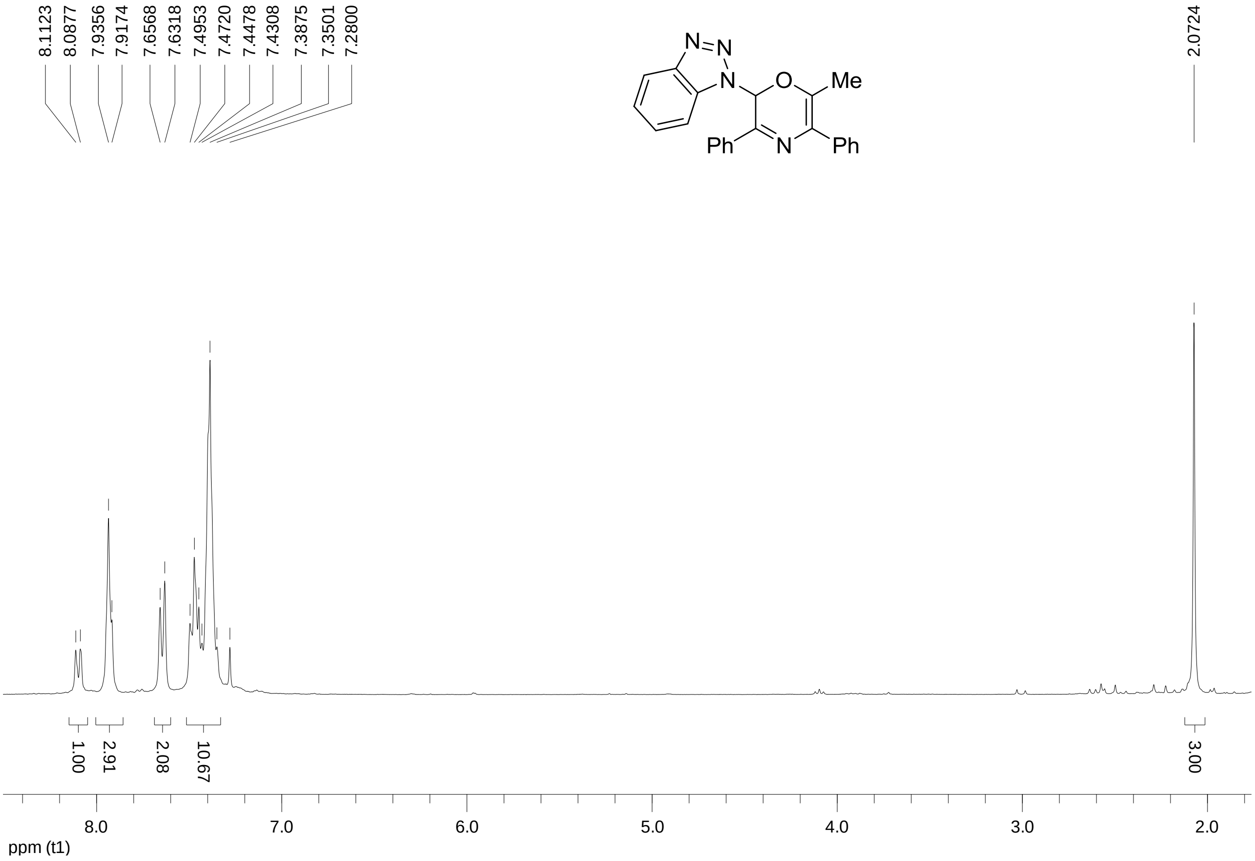

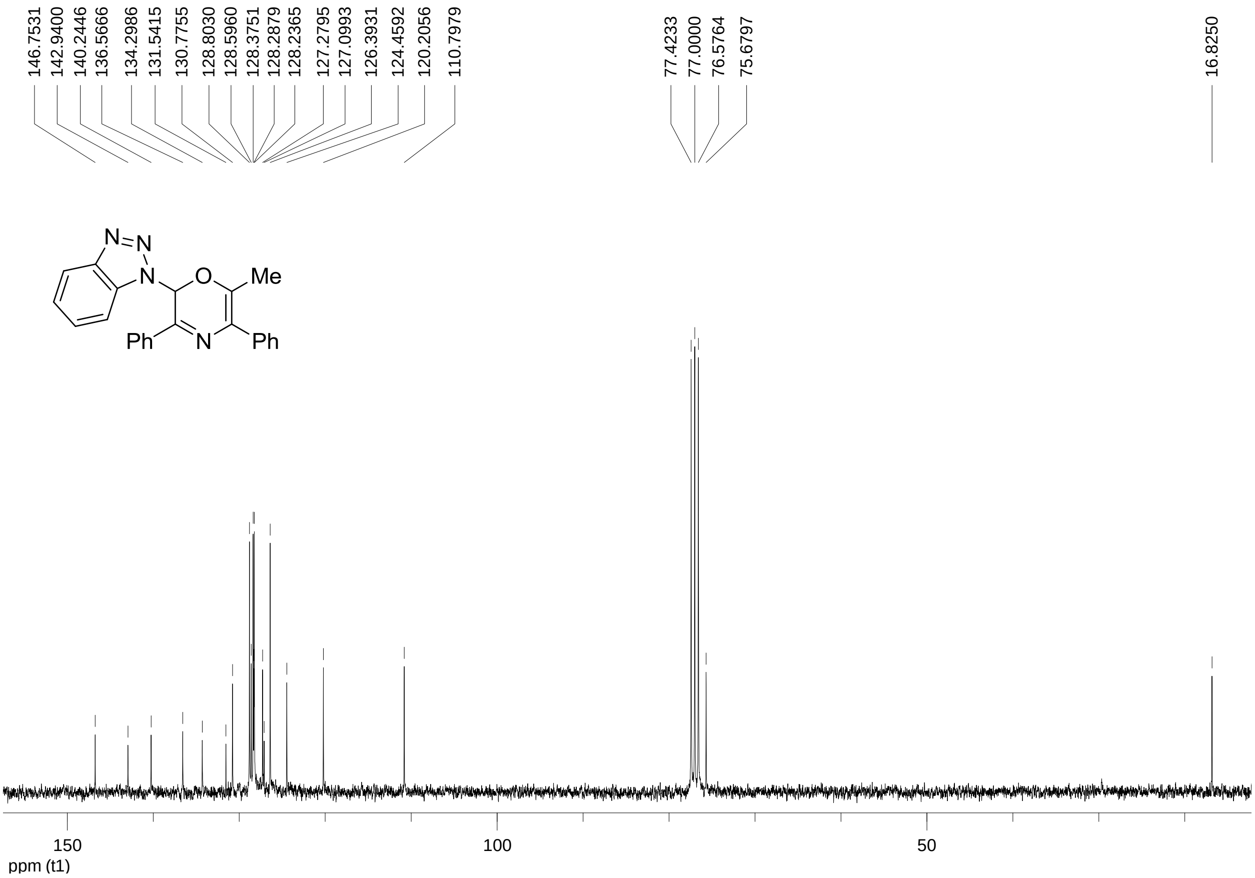

2,2,6-Trimethyl-3,5-diphenyl-2*H*-1,4-oxazine (4c)

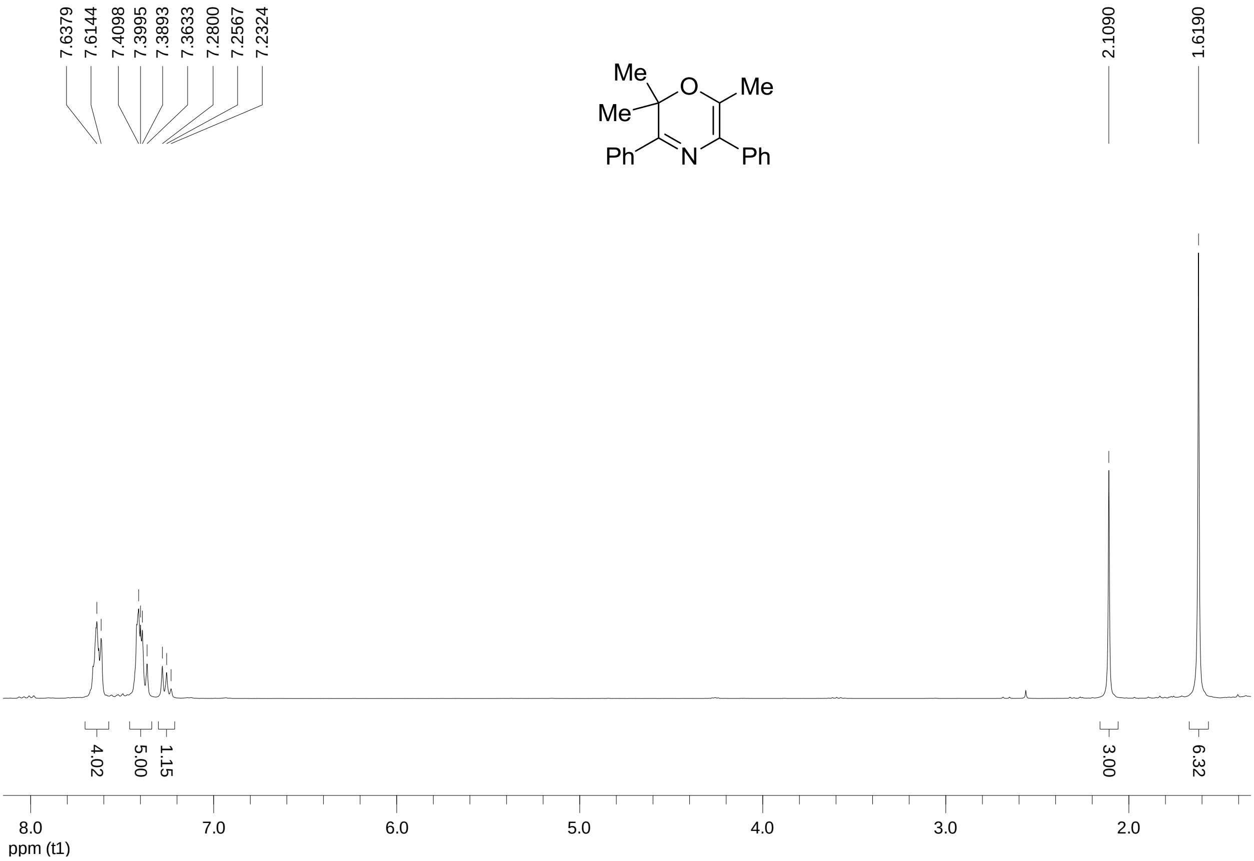

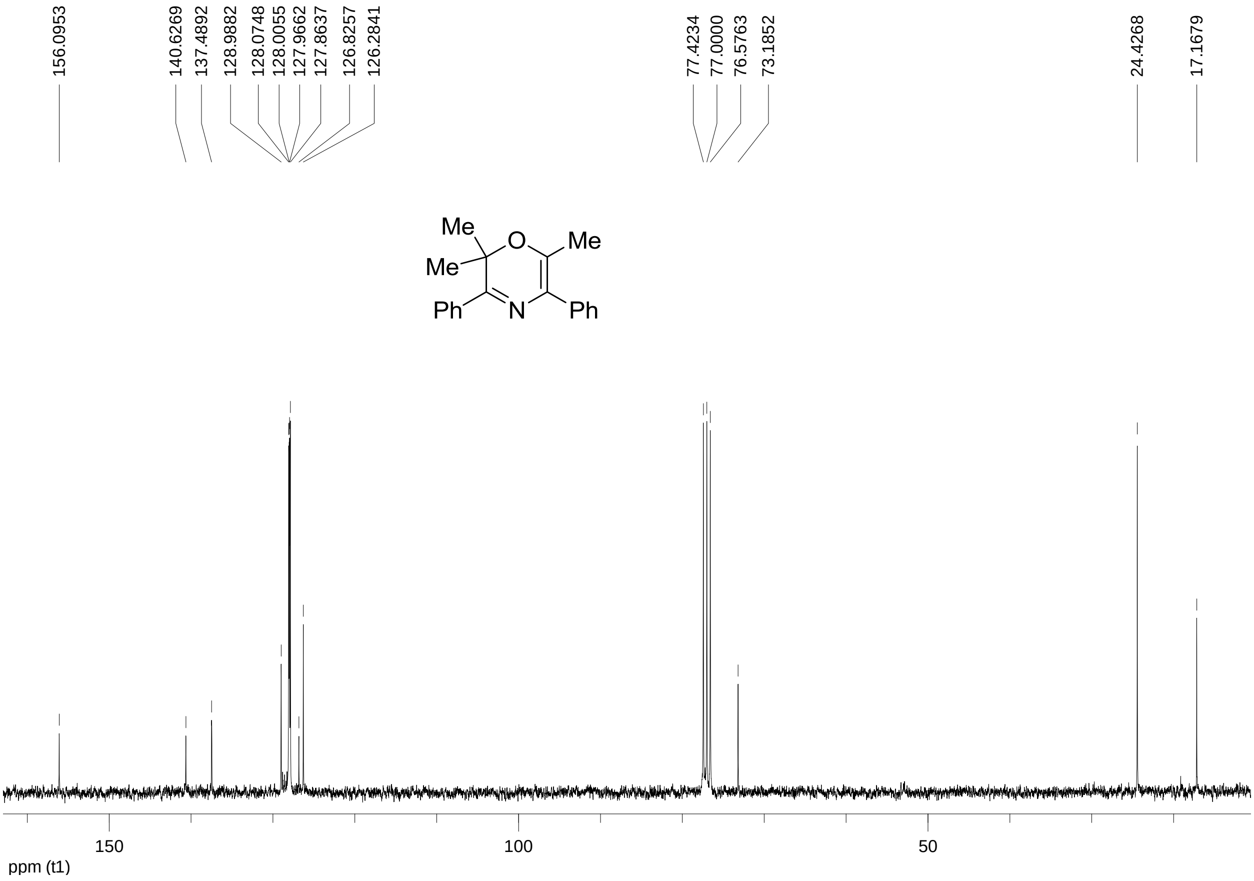

1-Phenyl-1-[(triphenylvinyl)imino]propane-2-one (3d)

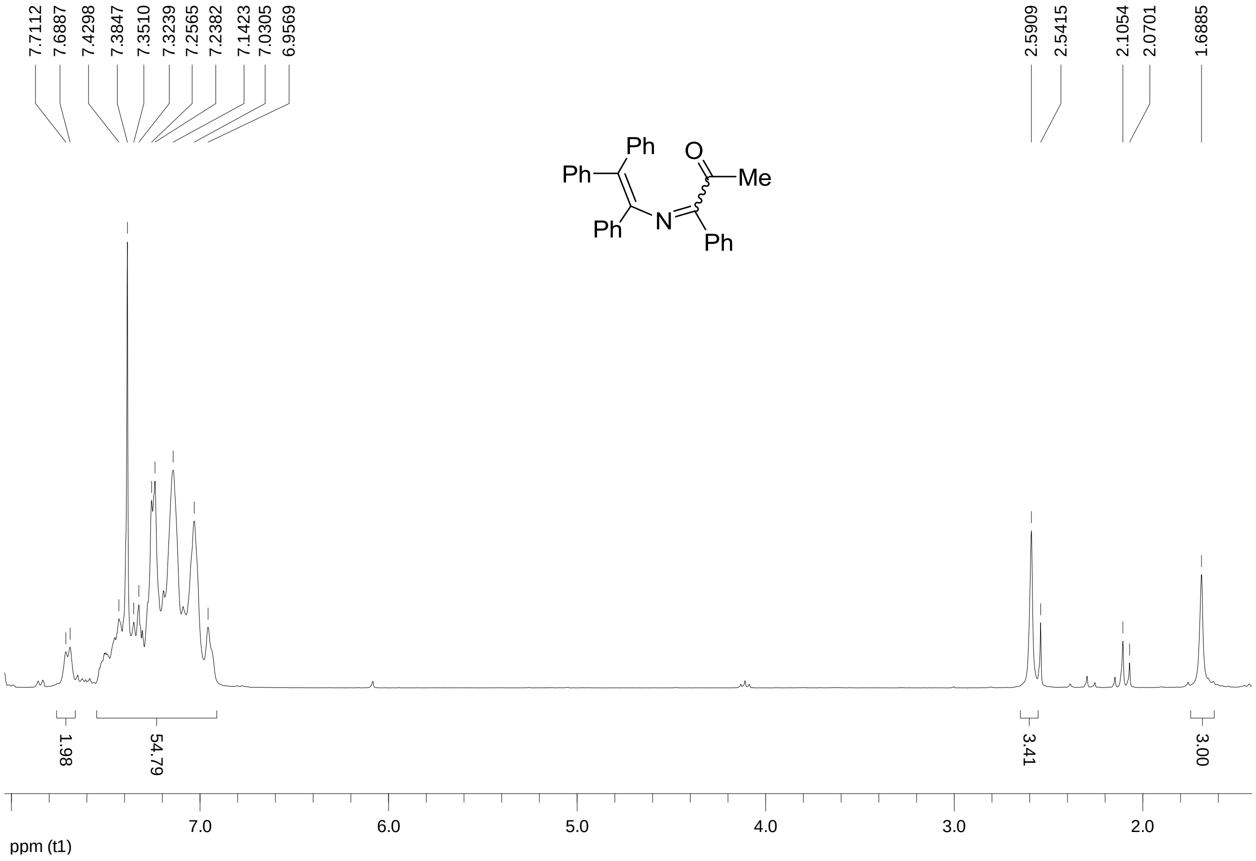

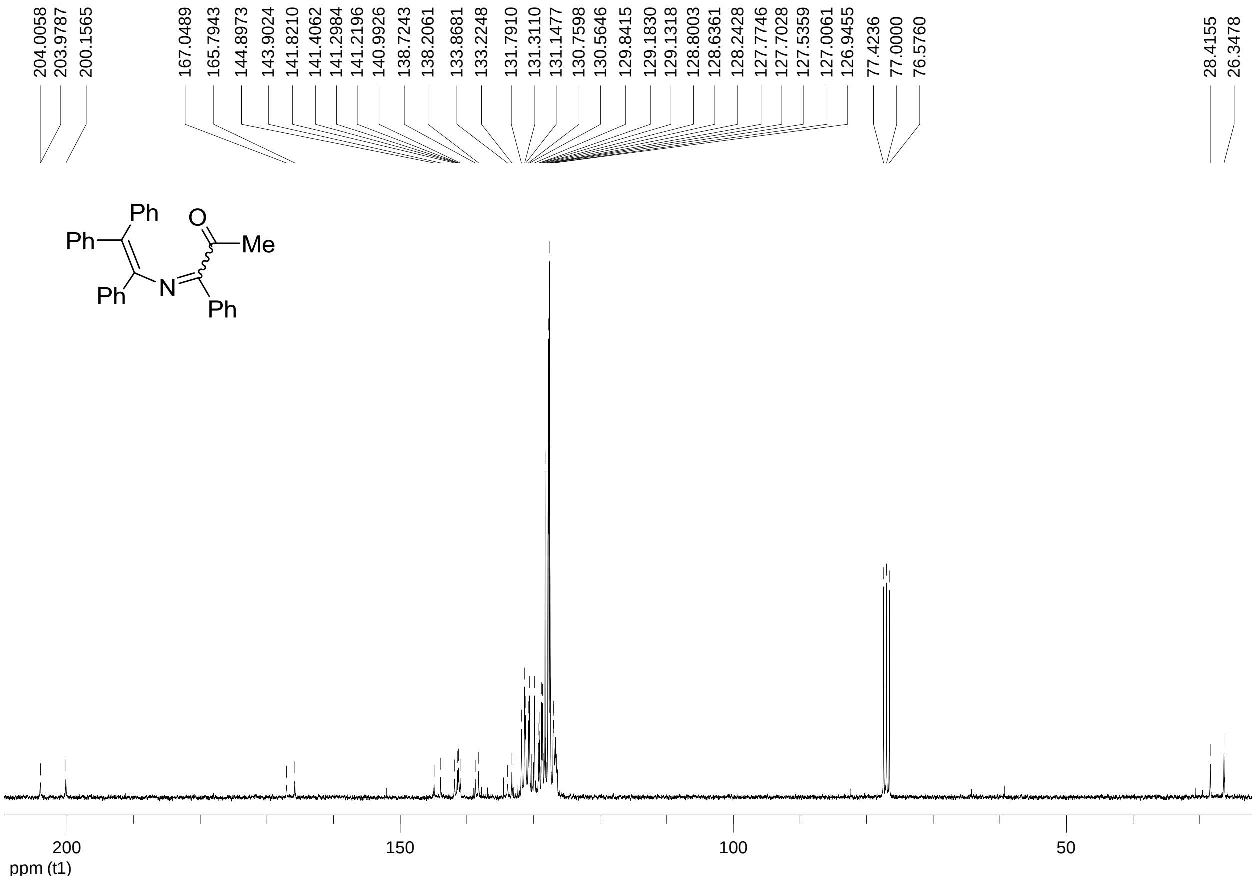

6-Methyl-2,2,3,5-tetraphenyl-2*H*-1,4-oxazine (4d)

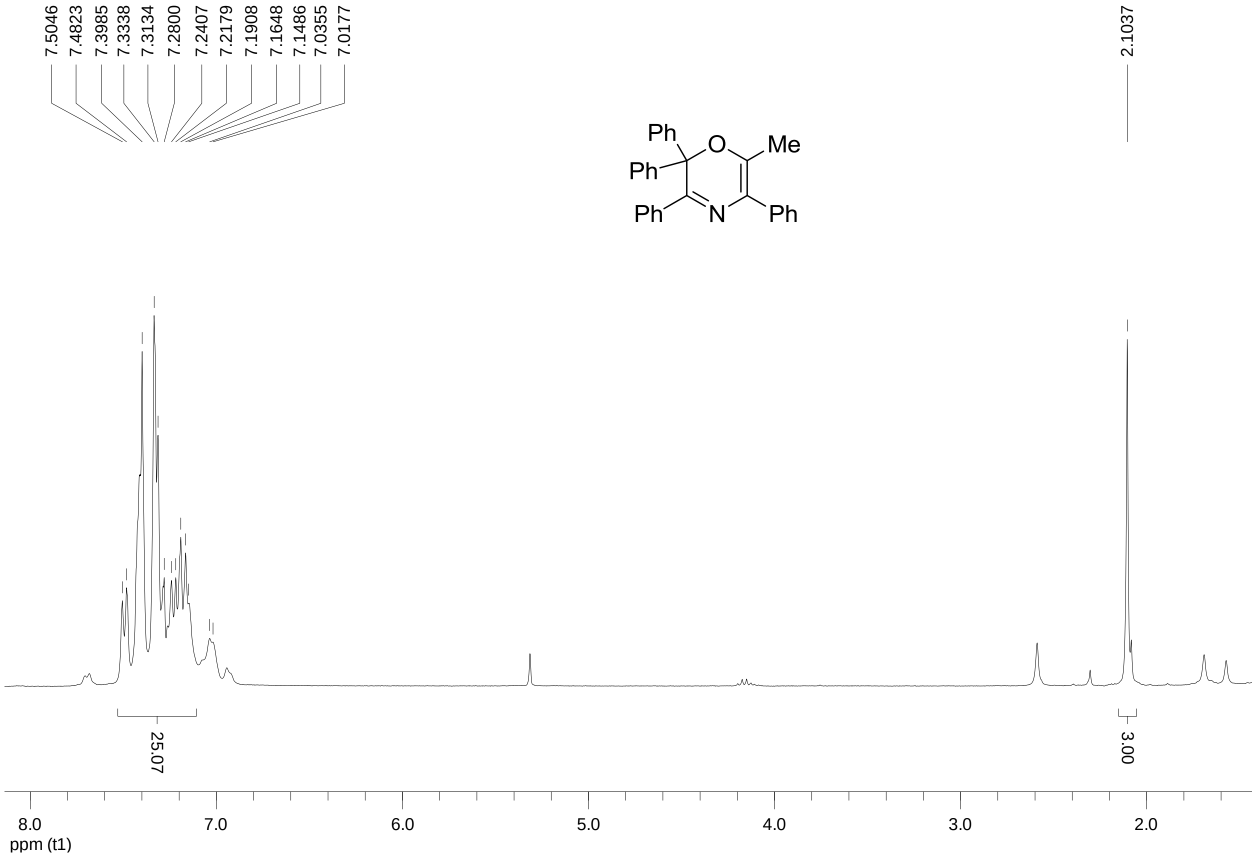

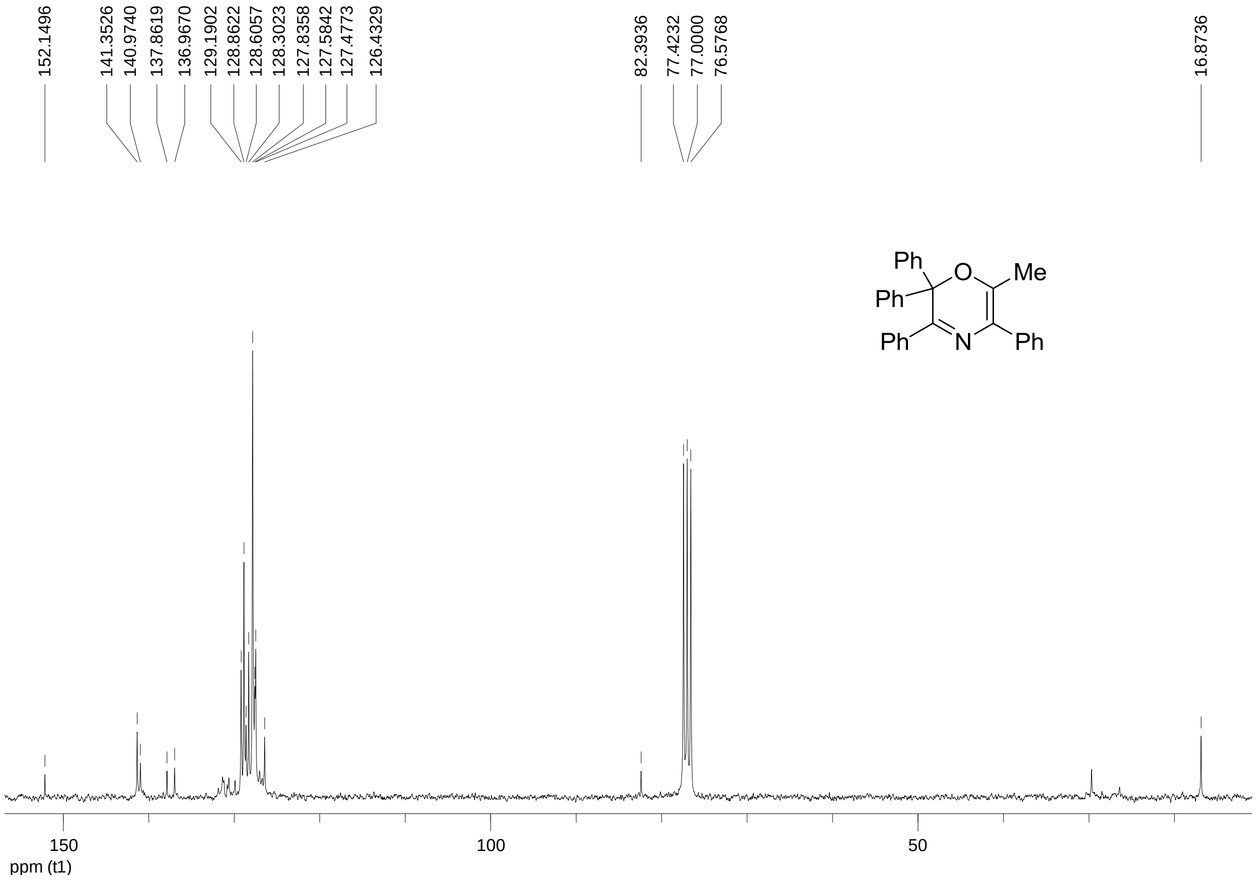

1-(4-Chlorophenyl)-2-[(2-methyl-1-phenylprop-1-enyl)imino]ethanone (3e)

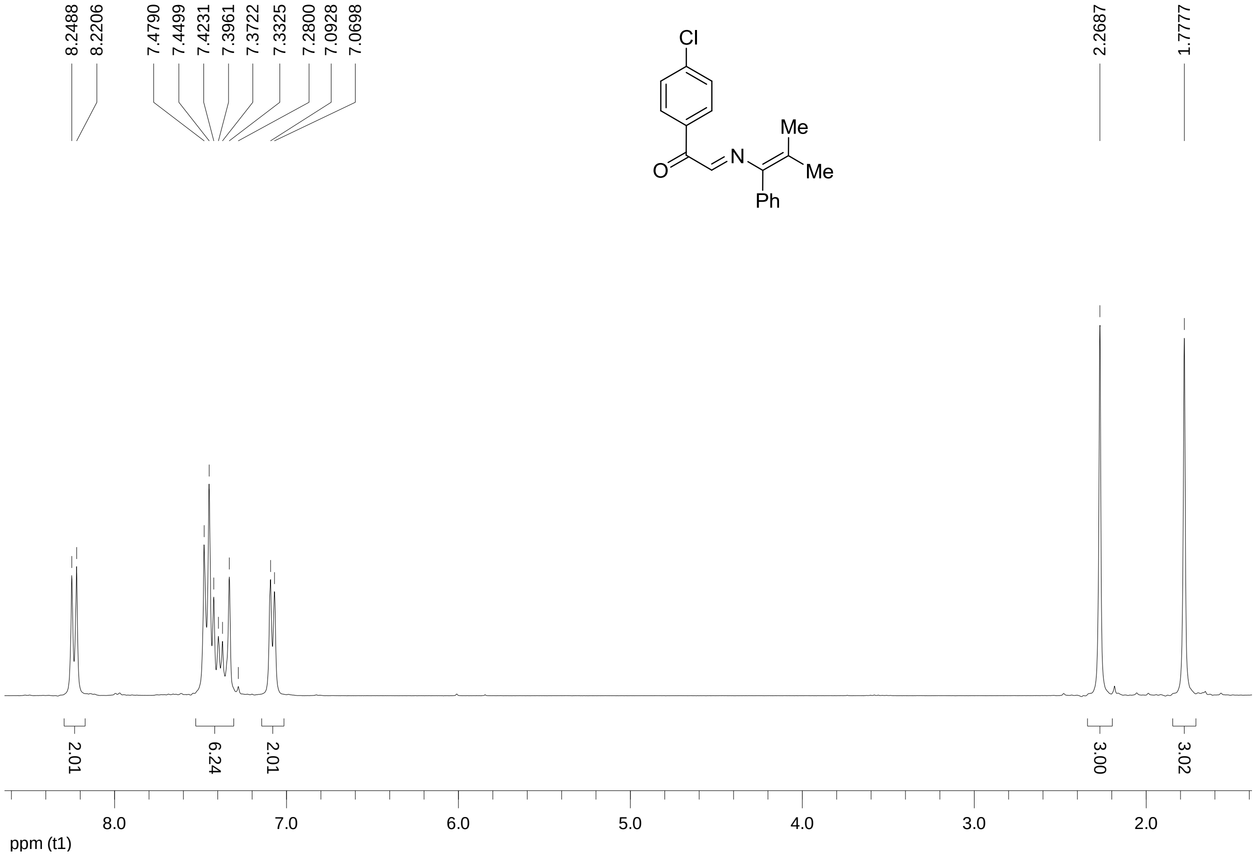

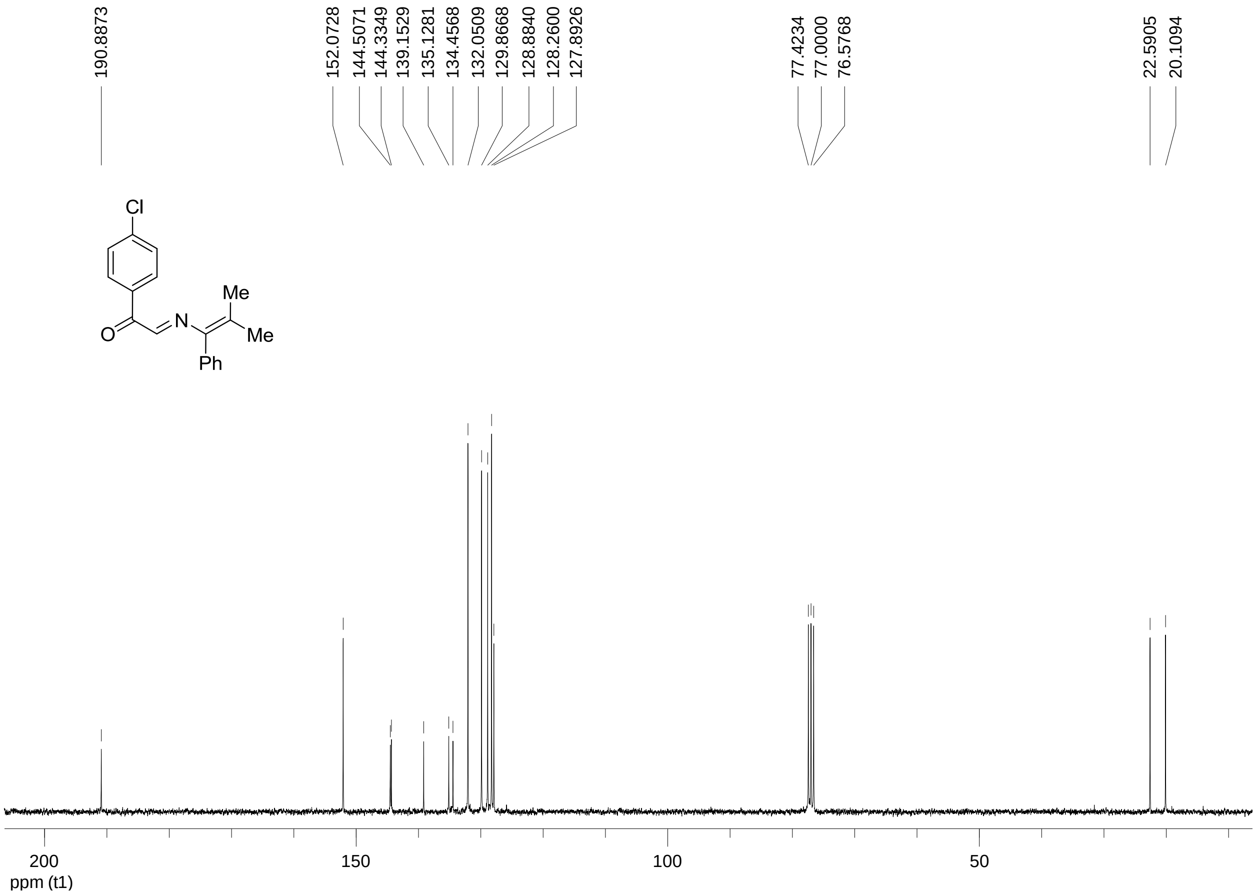

Thermal ellipsoid plot for the crystal structure 3e

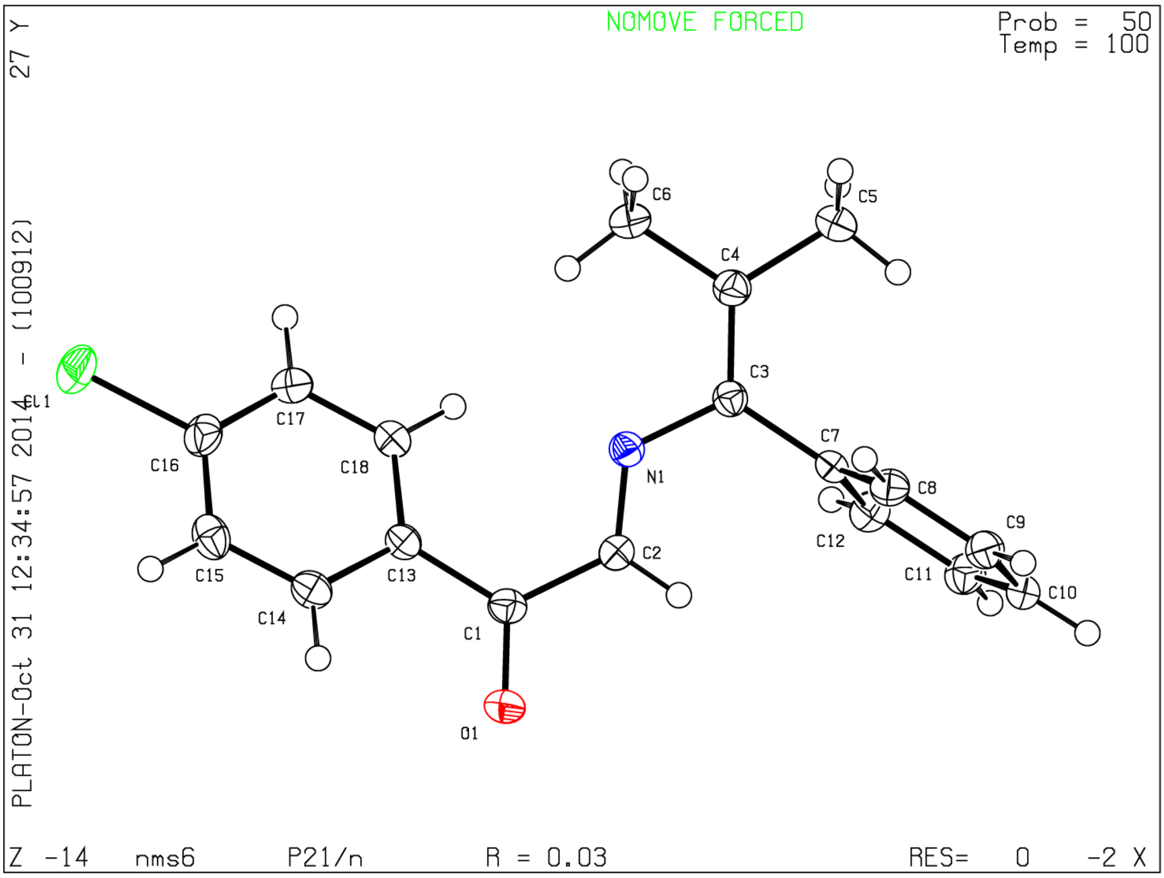

6-(4-Chlorophenyl)-2,2-dimethyl-3-phenyl-2*H*-1,4-oxazine (4e)

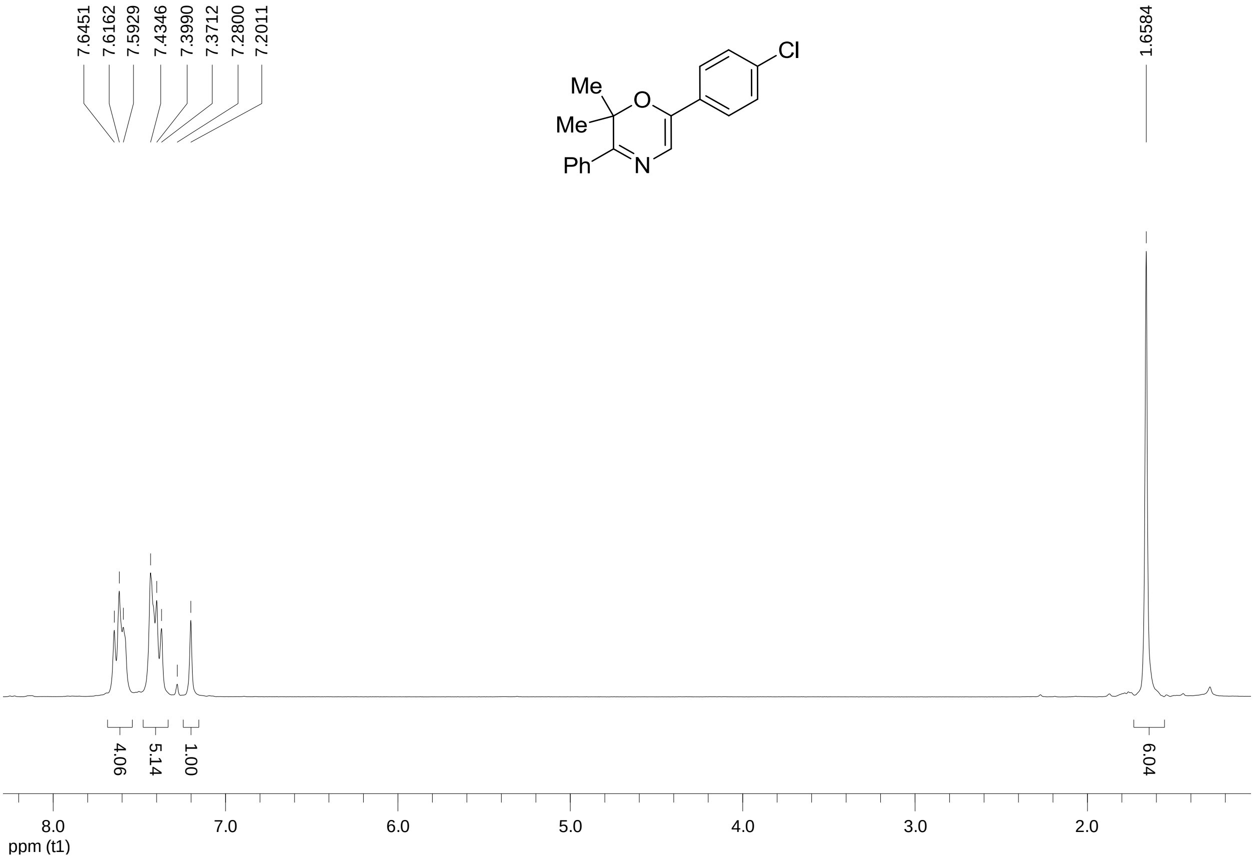

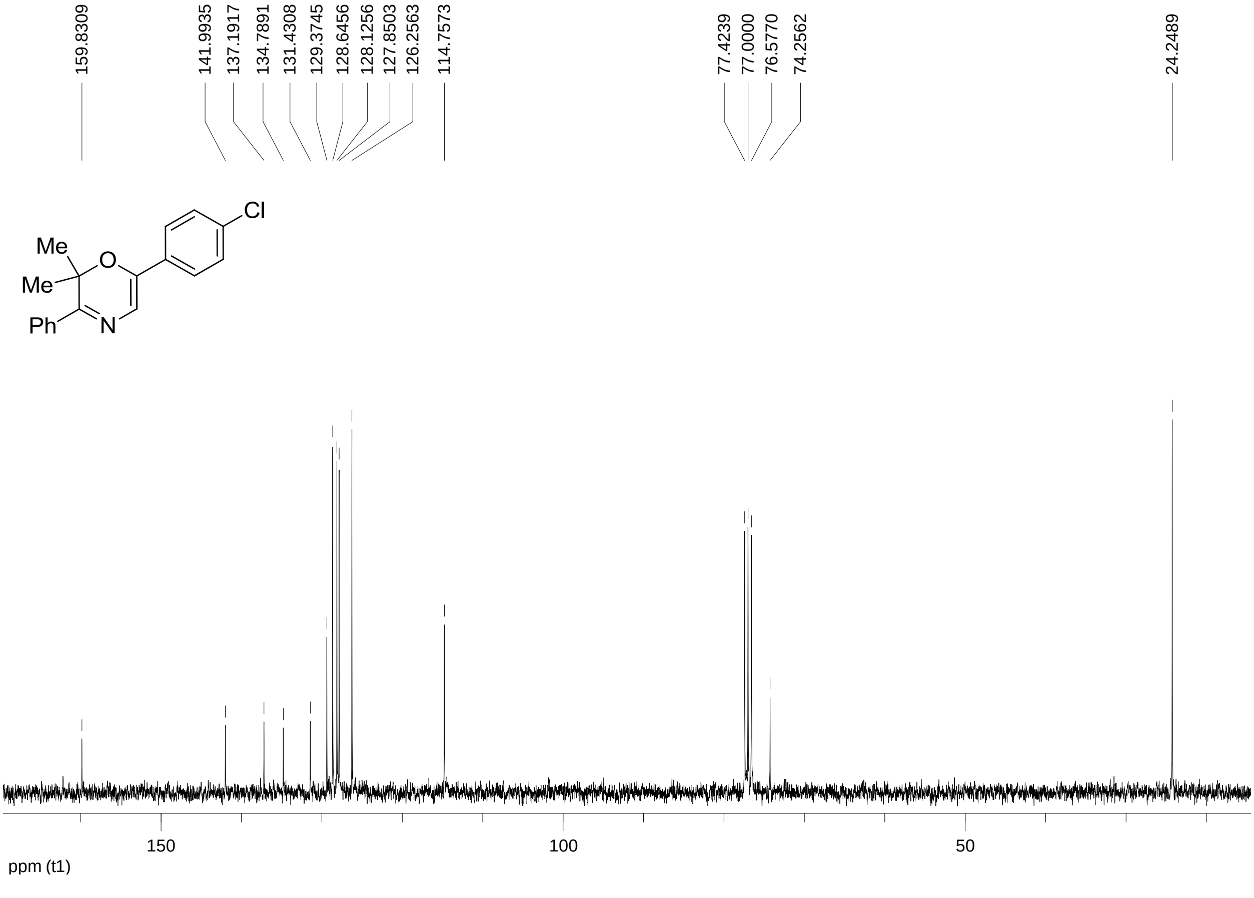

1-[6-Methyl-3-(4-methylphenyl)-2*H*-1,4-oxazin-5-yl]ethanone (4f)

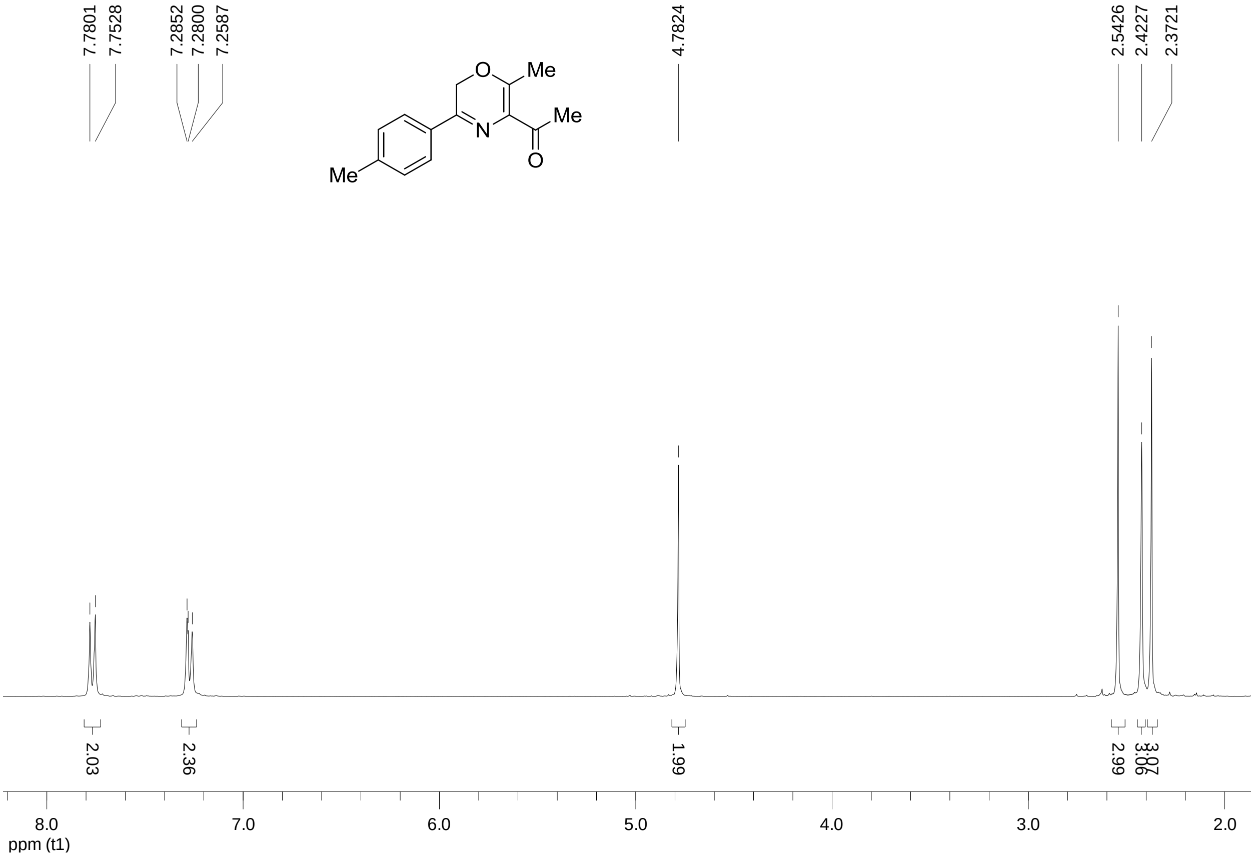

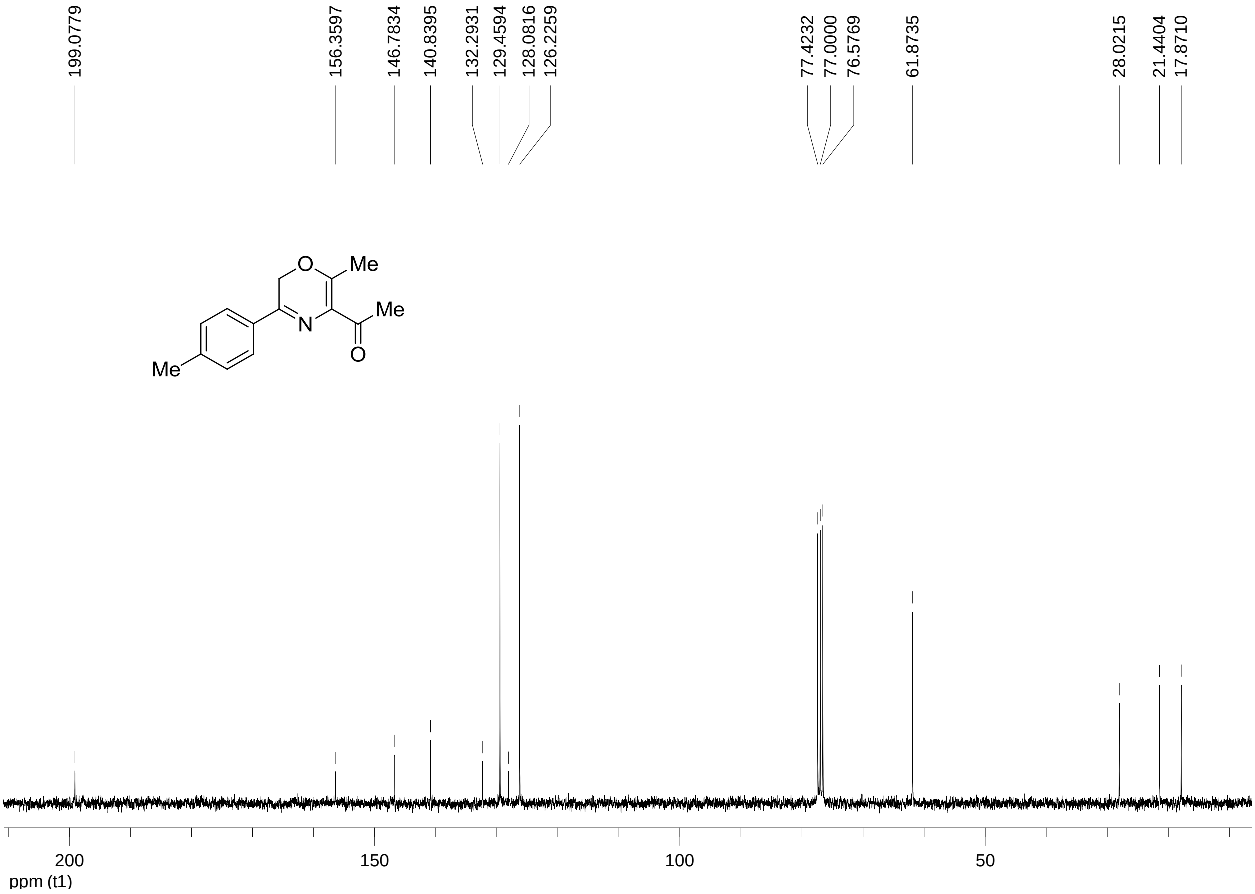

(*Z*)-3-[2-Acetyl-3-methyl-5-(4-methylphenyl)-4,6-dioxa-1-azabicyclo[3.2.1]oct-2-en-7-yliden]butane-2-one (6f)

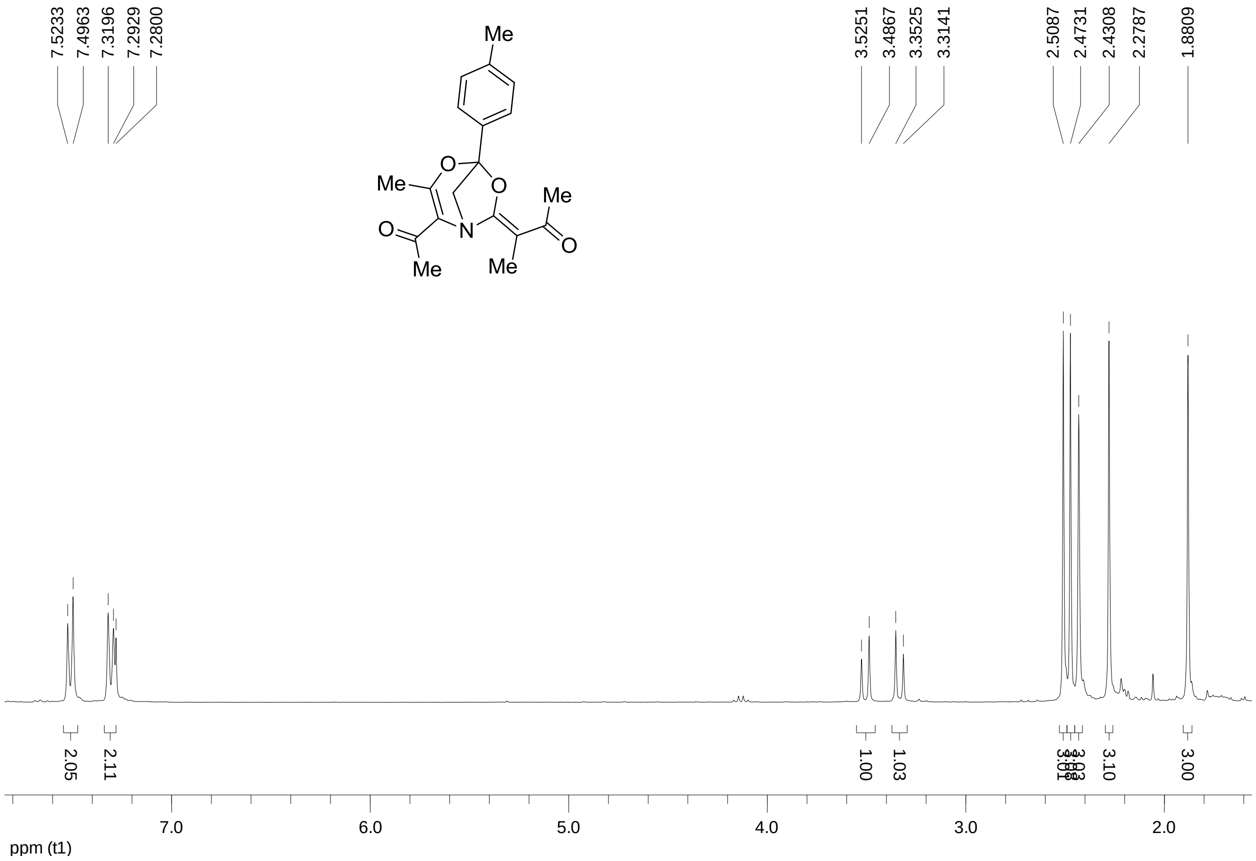

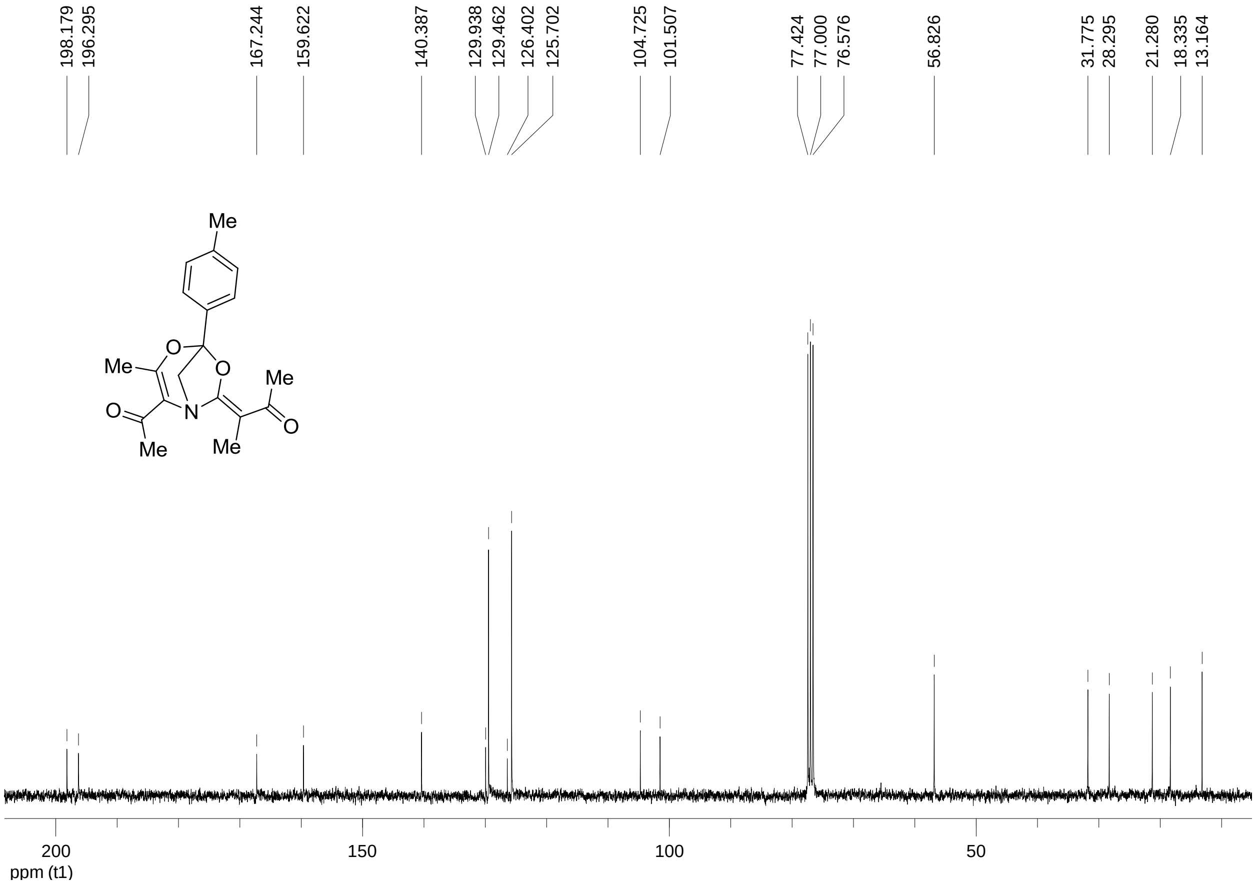

Thermal ellipsoid plot for the crystal structure 6f

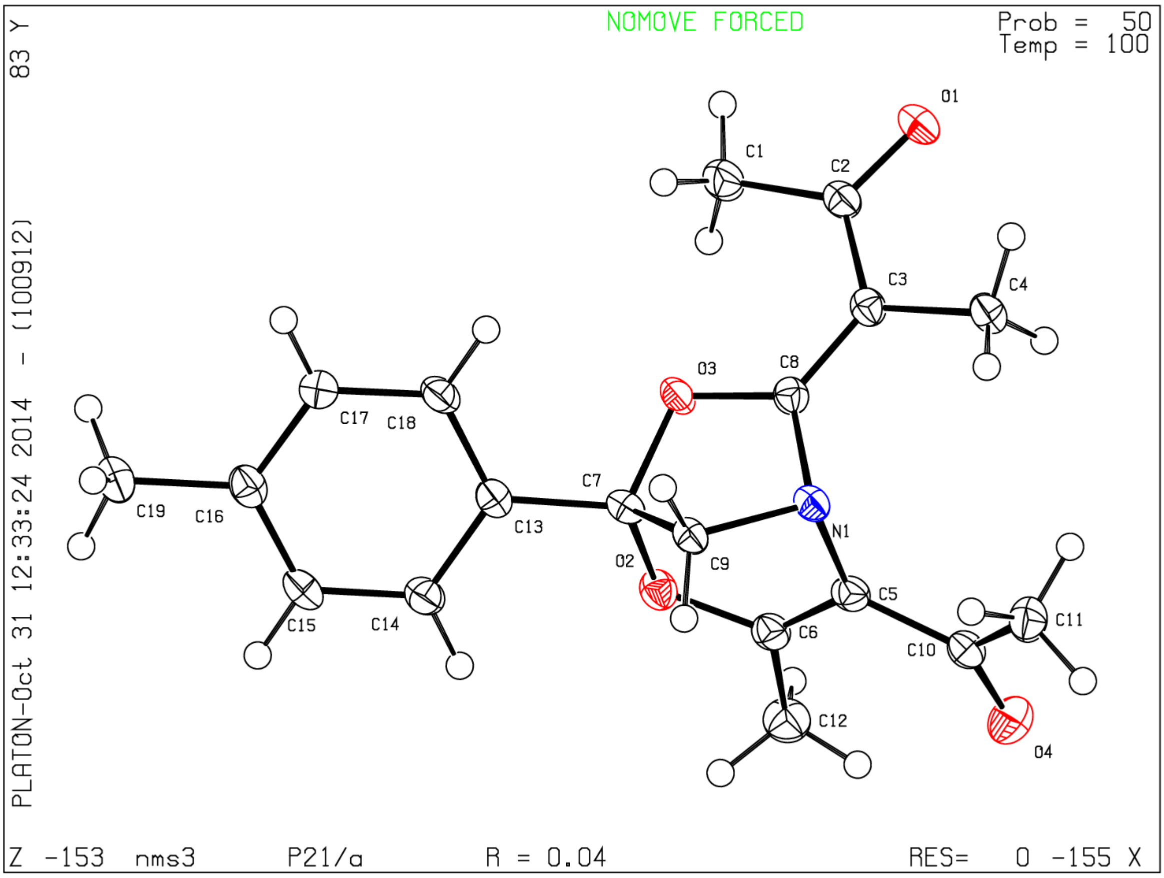

9-Acetyl-3,4,8-trimethyl-6-(4-methylphenyl)-5,7-dioxa-1-azabicyclo[4.3.1]deca-3,8-diene-2-one (7f)

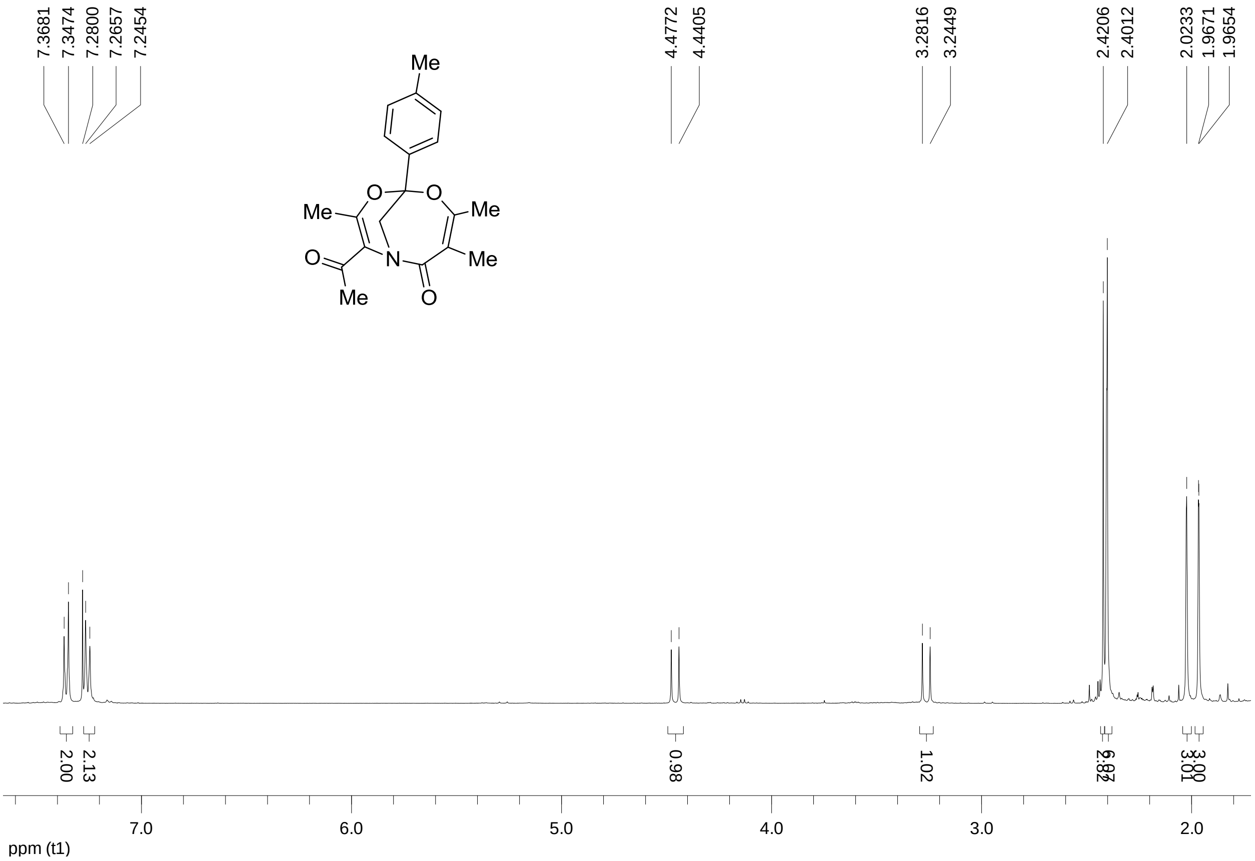

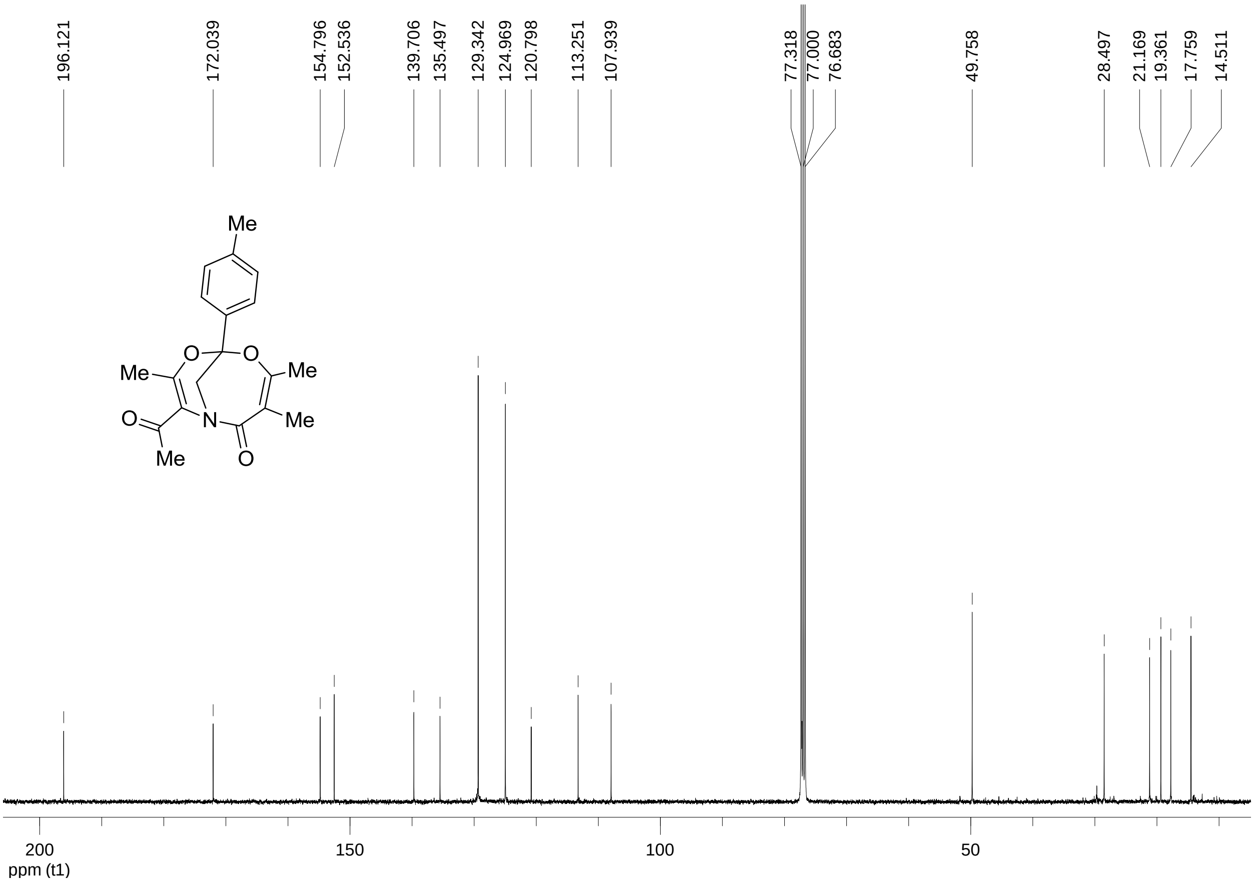

6-(4-Methylphenyl)-3,4,8,9-tetramethyl-5,7-dioxa-1-azabicylo[4.4.1]undeca-3,8-diene-2,10-dione (8f)

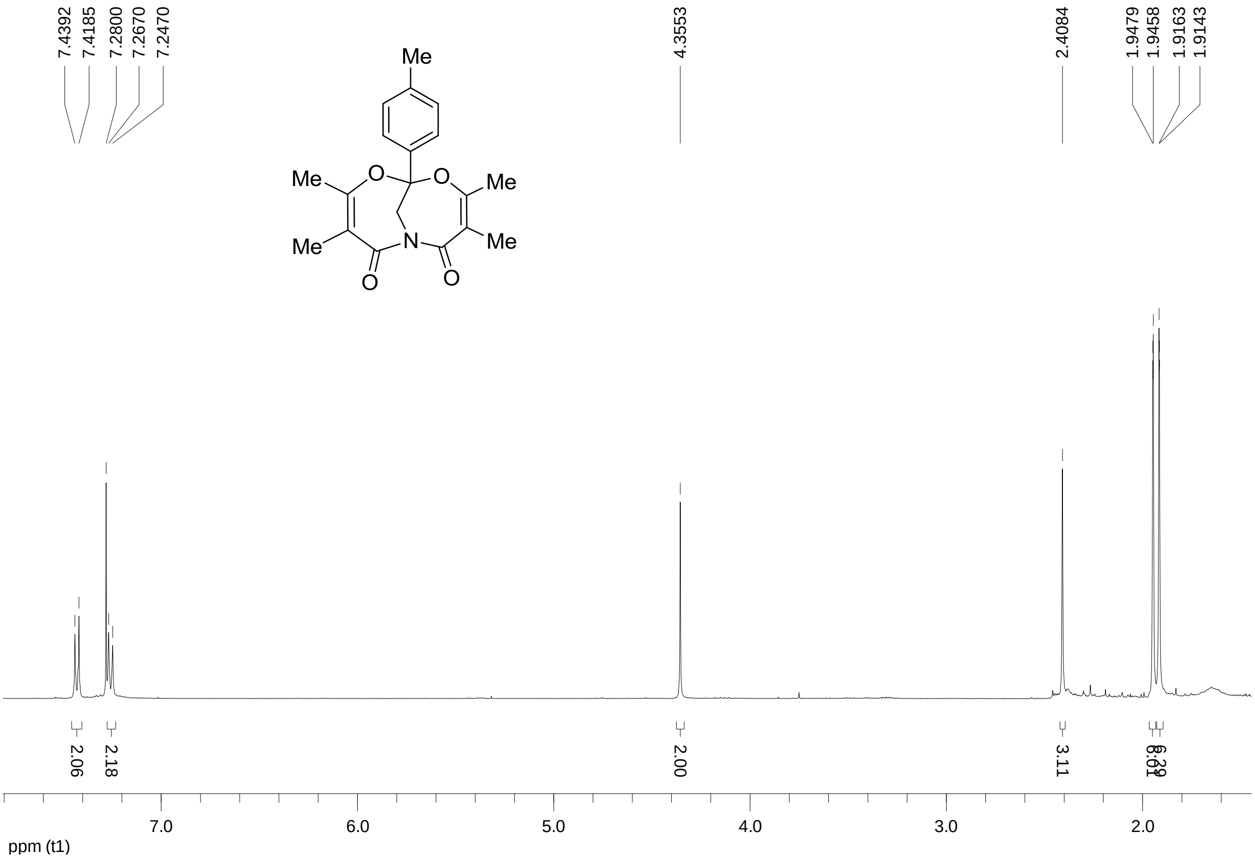

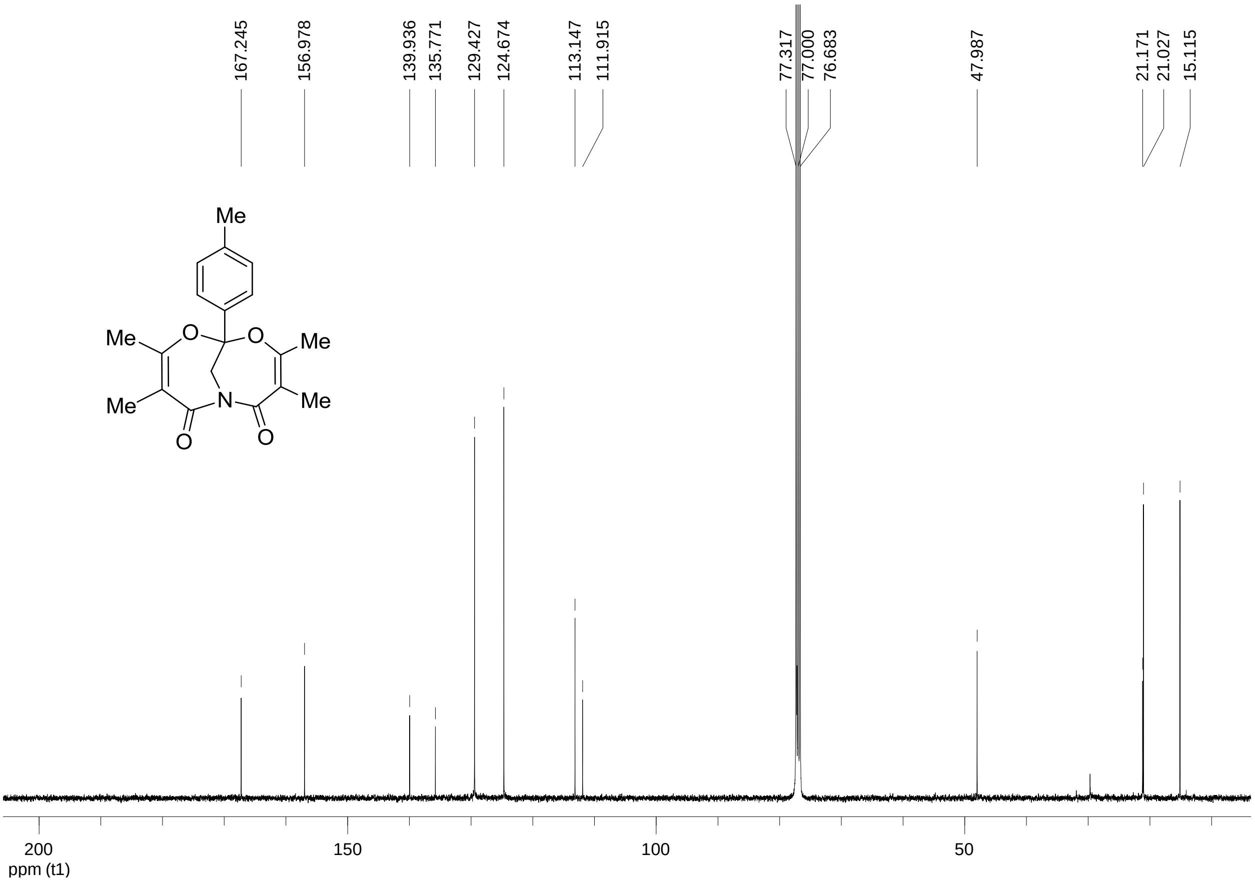

1-[6-Methyl-3-(4-methoxyphenyl)-2*H*-1,4-oxazin-5-yl]ethanone (4g)

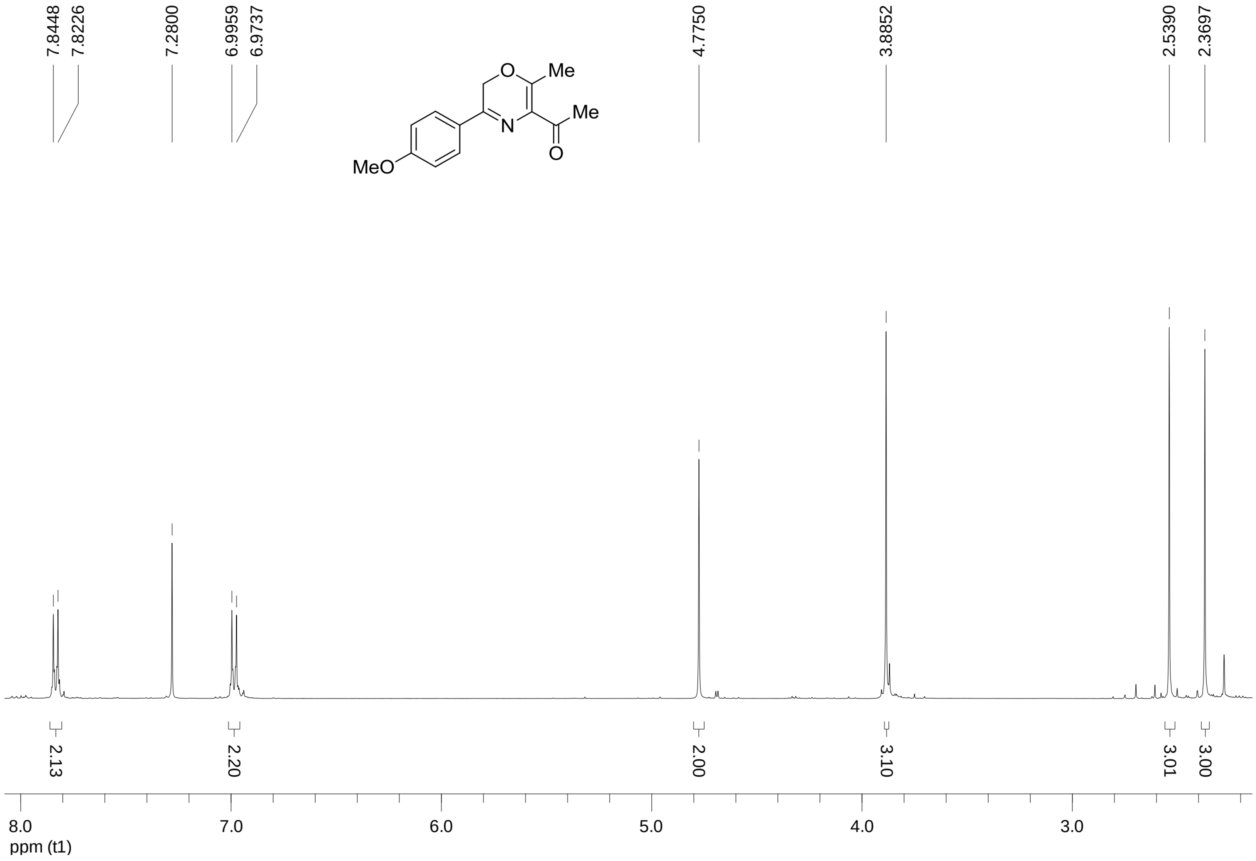

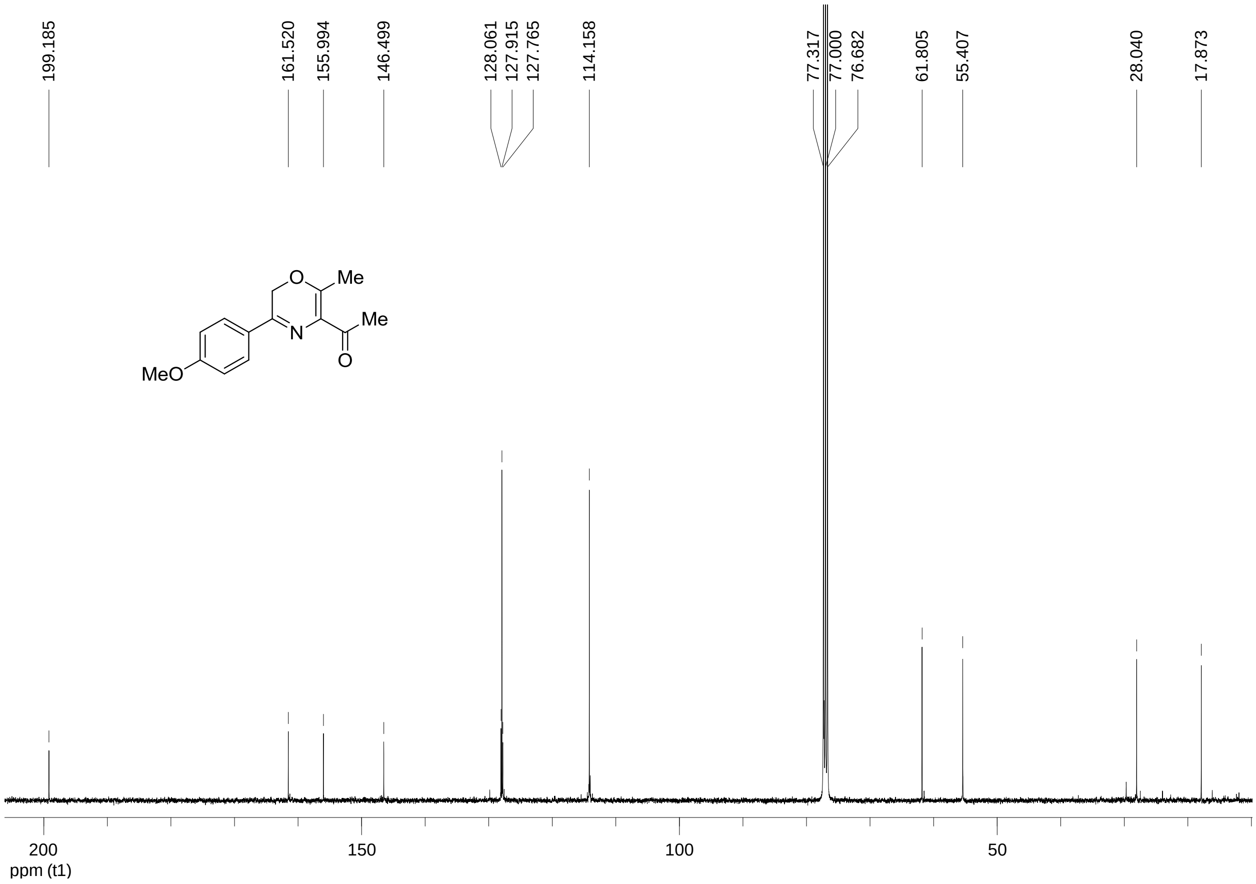

9-Acetyl-3,4,8-trimethyl-6-(4-methoxyphenyl)-5,7-dioxa-1-azabicyclo[4.3.1]deca-3,8-diene-2-one (7g)

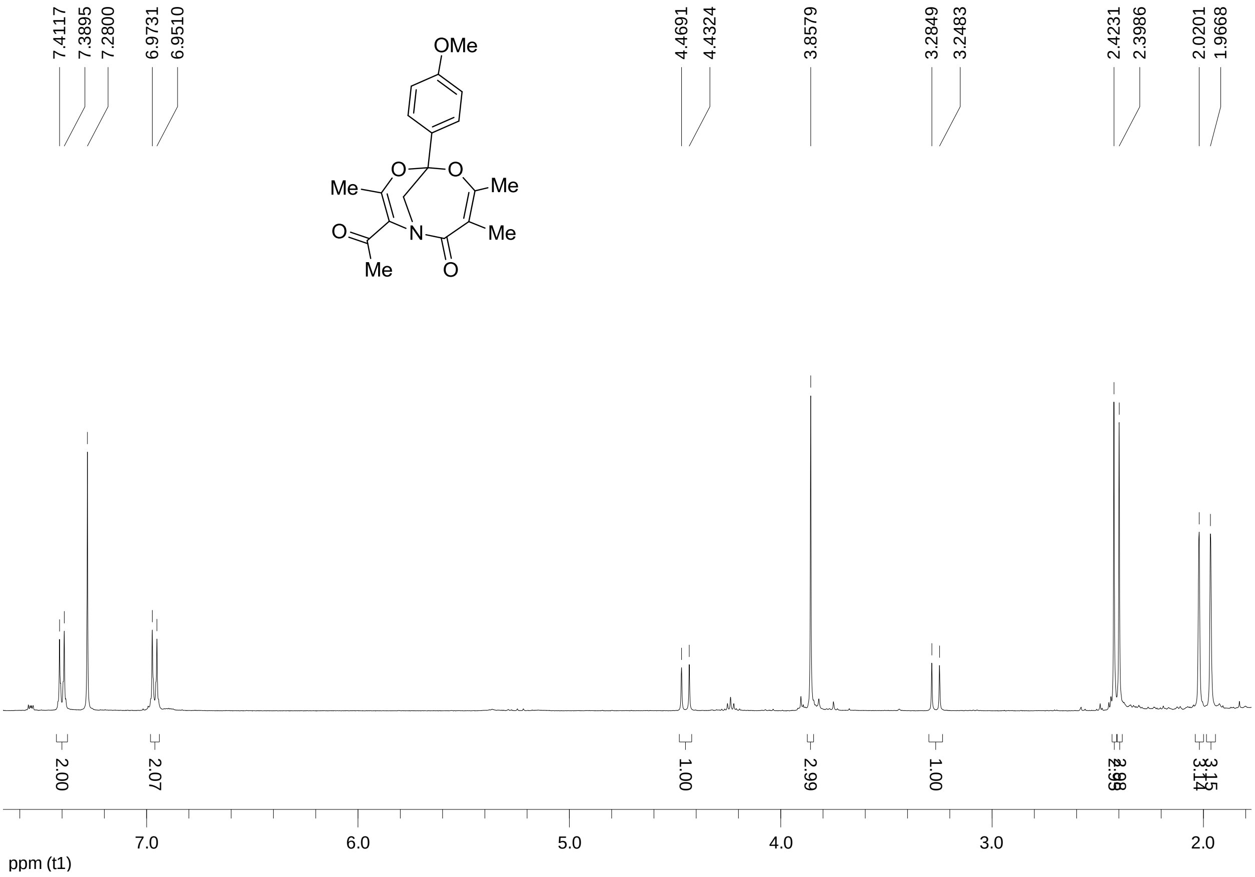

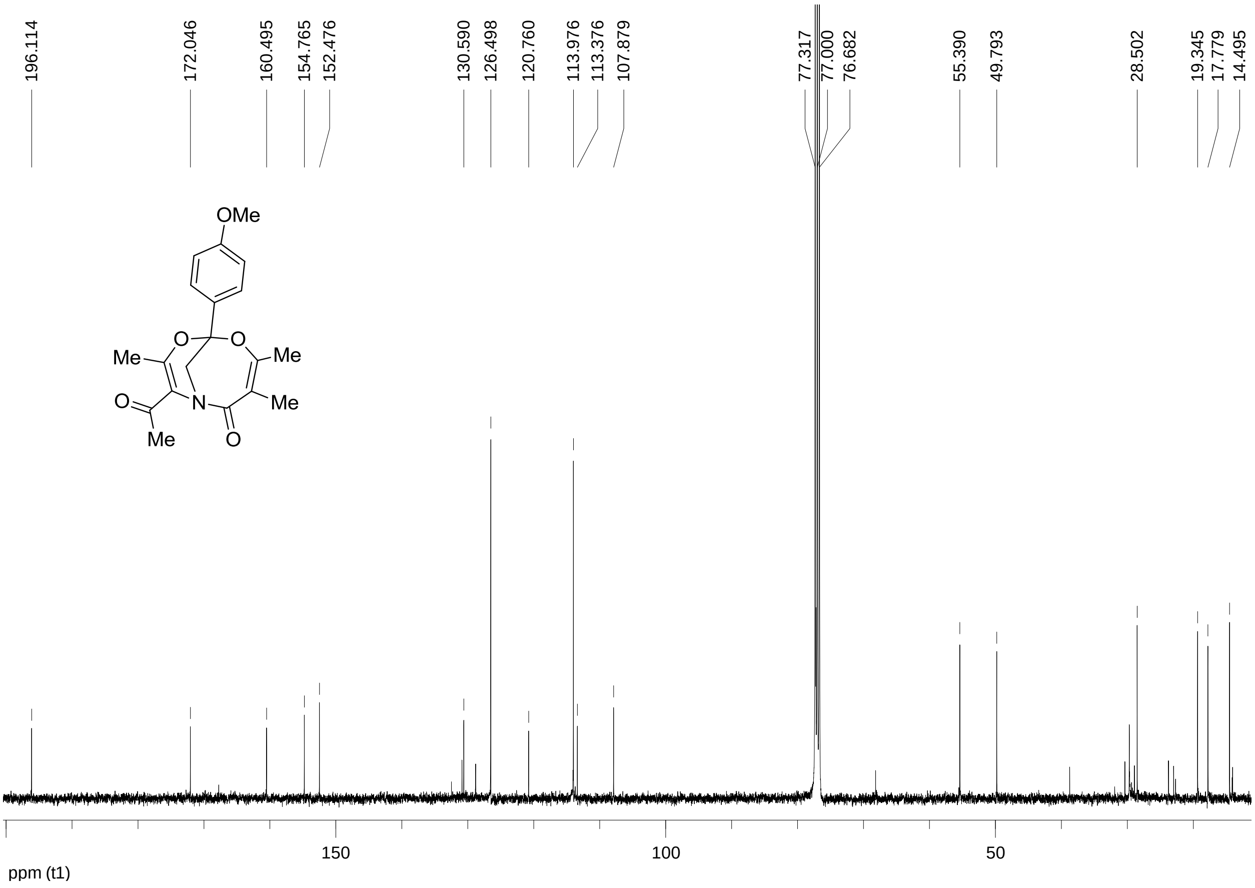

The signals of compounds 6g and 8g in the reaction mixture

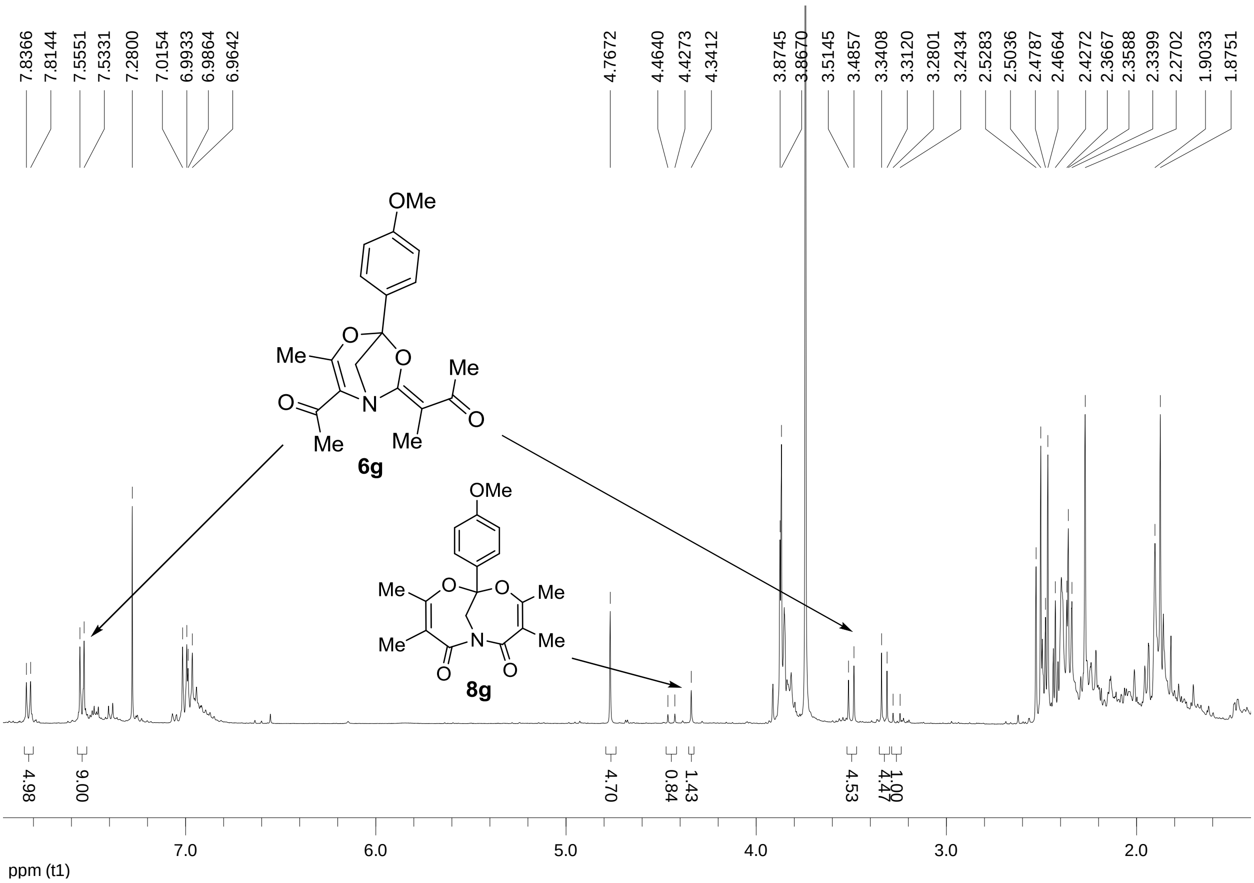

1-[6-Methyl-3-(4-nitrophenyl)-2*H*-1,4-oxazin-5-yl]ethanone (4h)

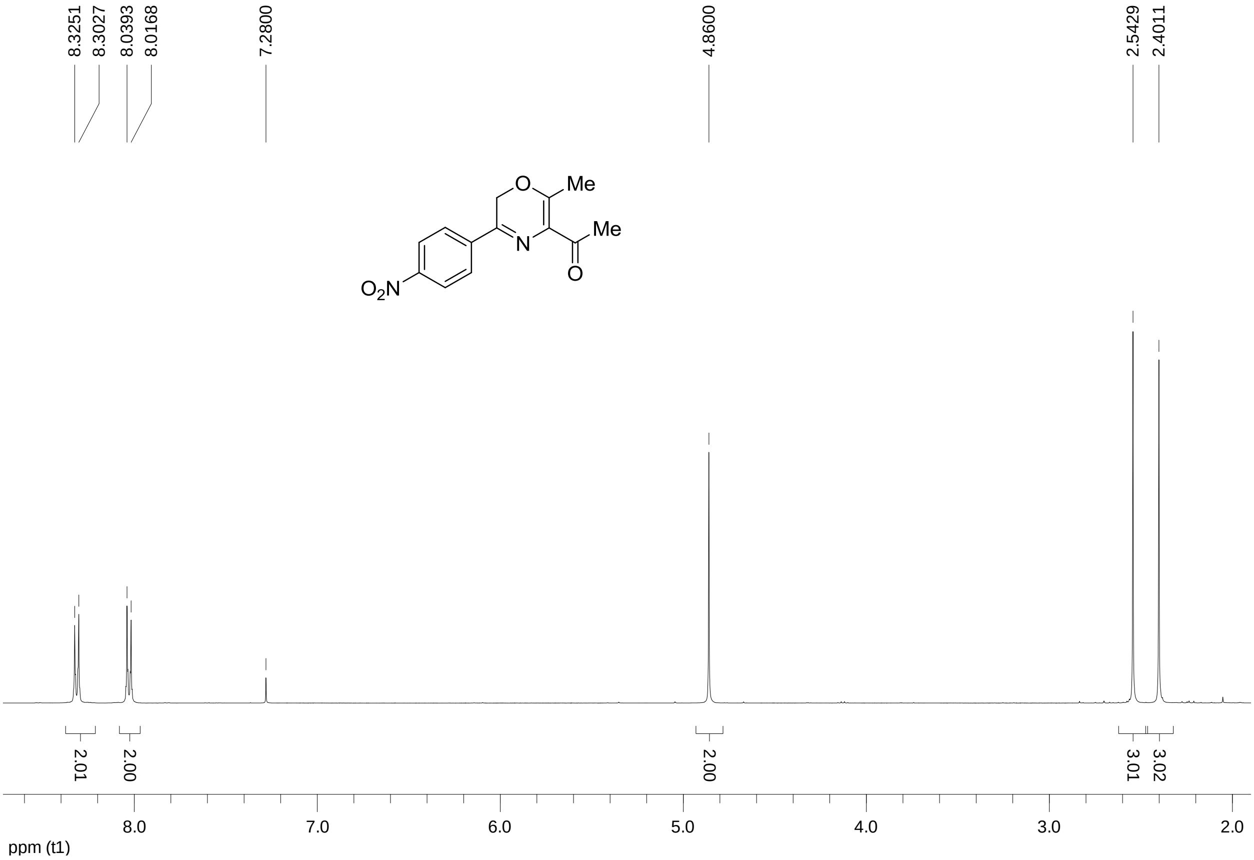

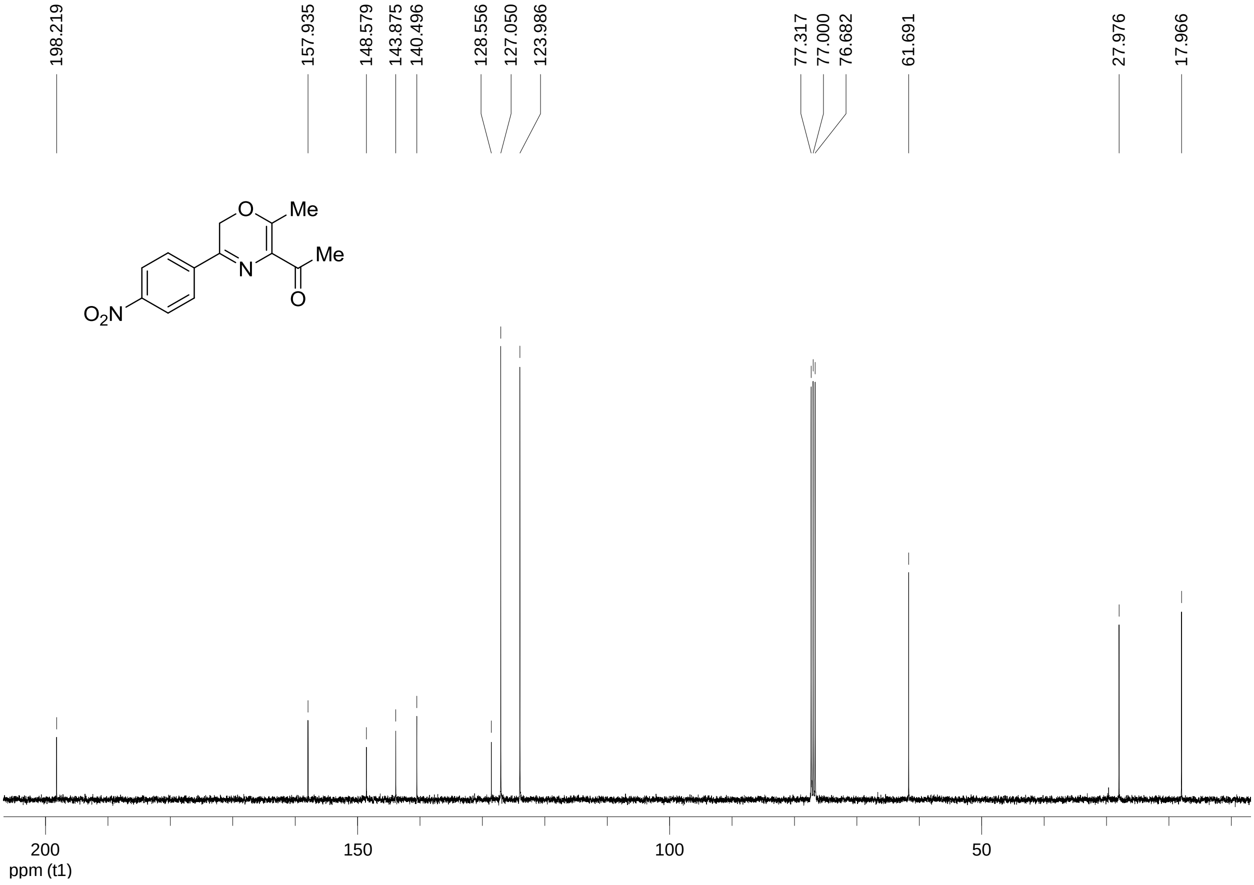

9-Acetyl-3,4,8-trimethyl-6-(4-nitrophenyl)-5,7-dioxa-1-azabicyclo[4.3.1]deca-3,8-diene-2-one (7h)

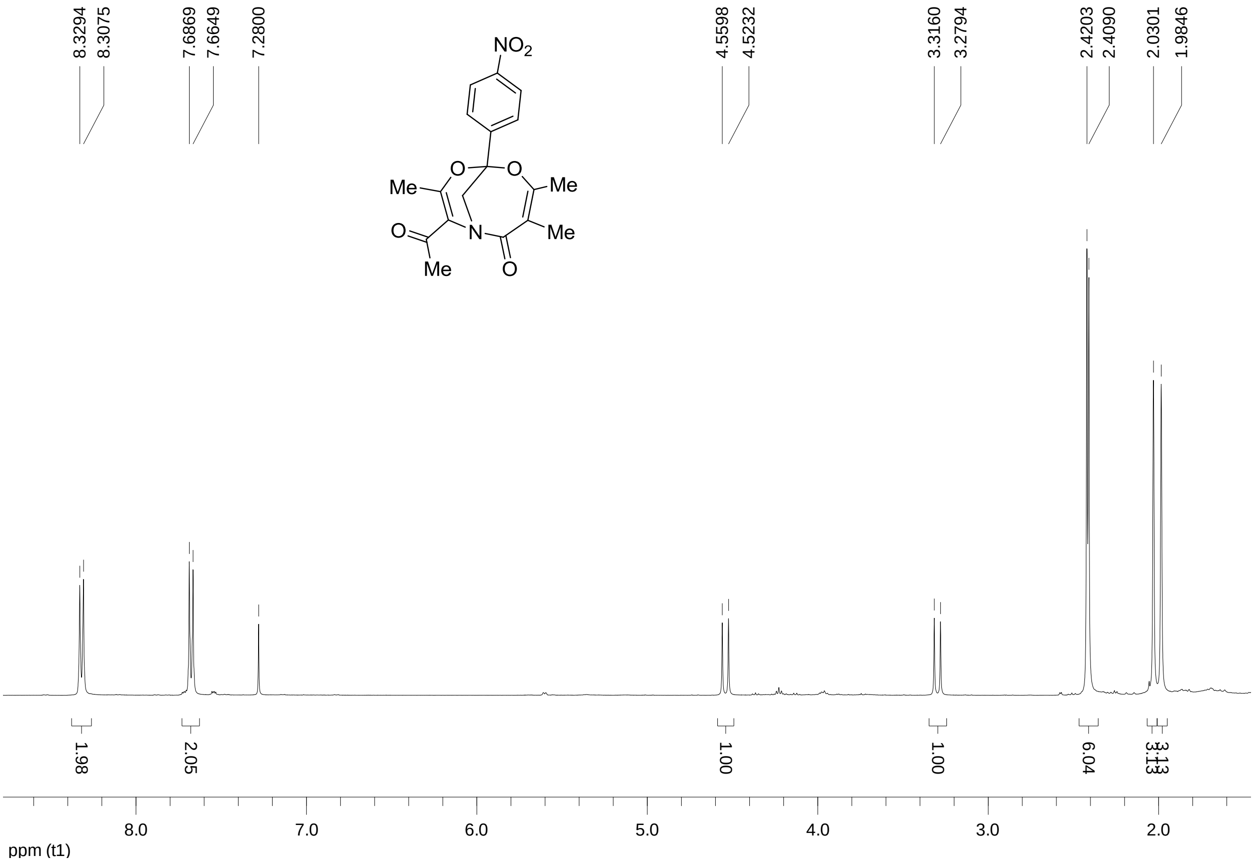

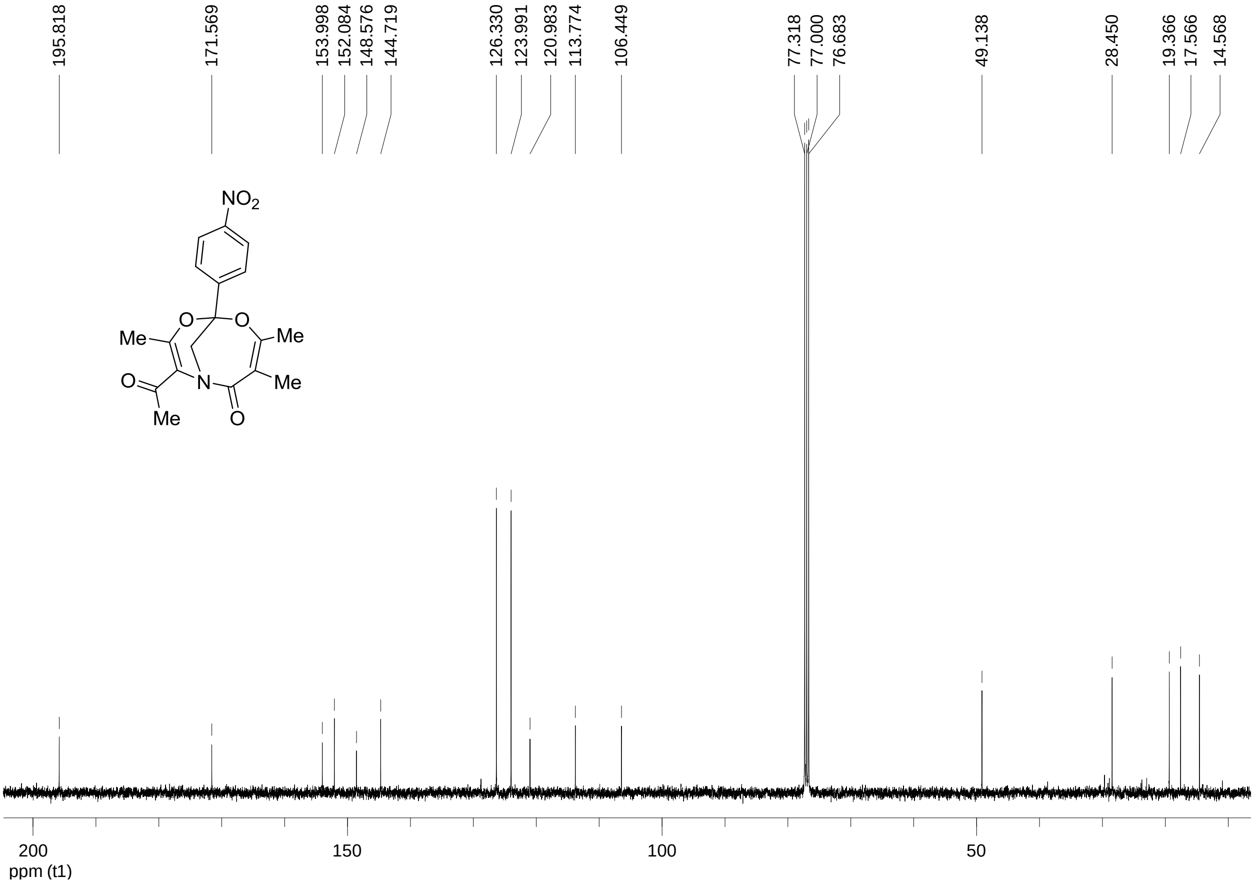

Thermal ellipsoid plot for the crystal structure 7h

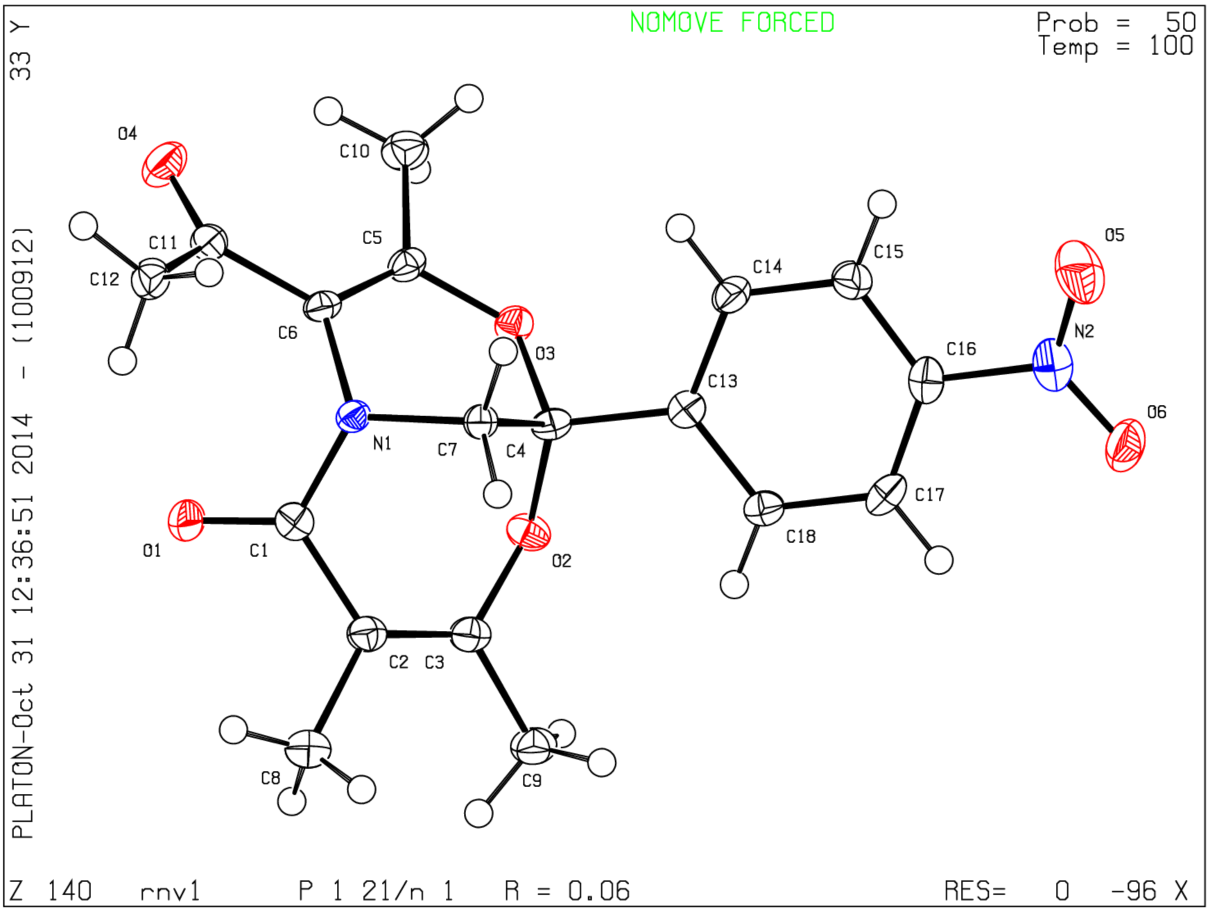

1-(6-Methyl-2,3-diphenyl-2*H*-1,4-oxazin-5-yl)ethanone (4i)

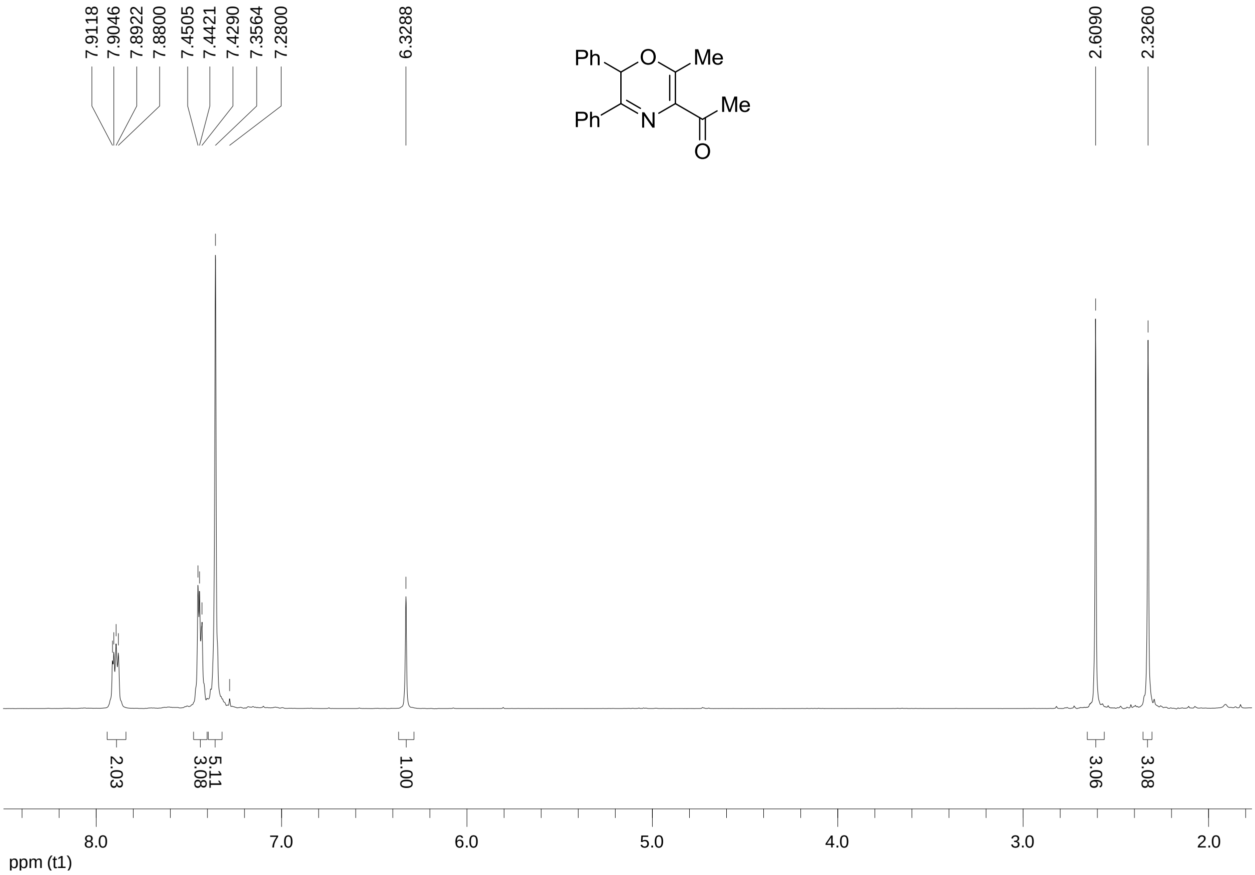

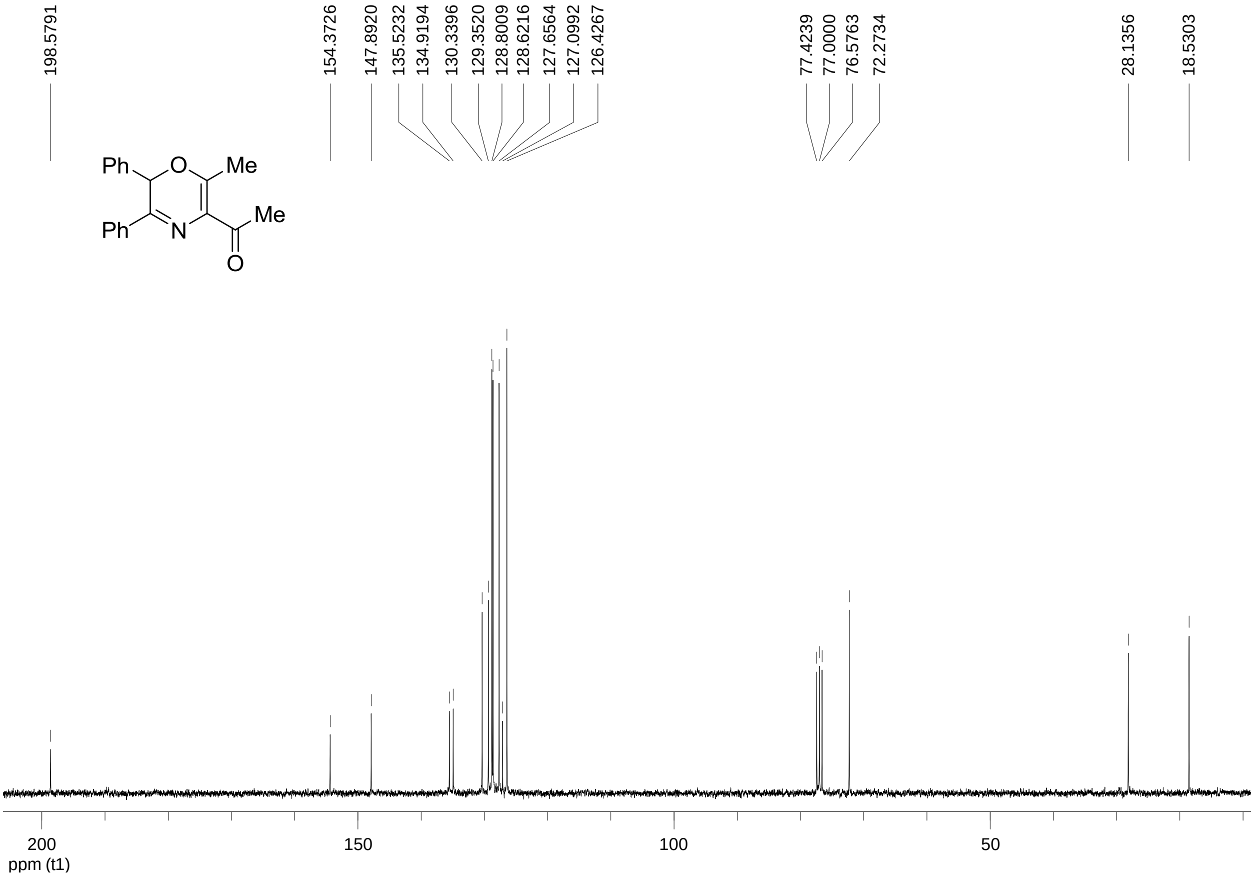

Ethyl 3,4,8-trimethyl-6-(4-nitrophenyl)-2-oxo-5,7-dioxa-1-azabicyclo[4.3.1]deca-3,8-diene- 9-carboxylate (7k)

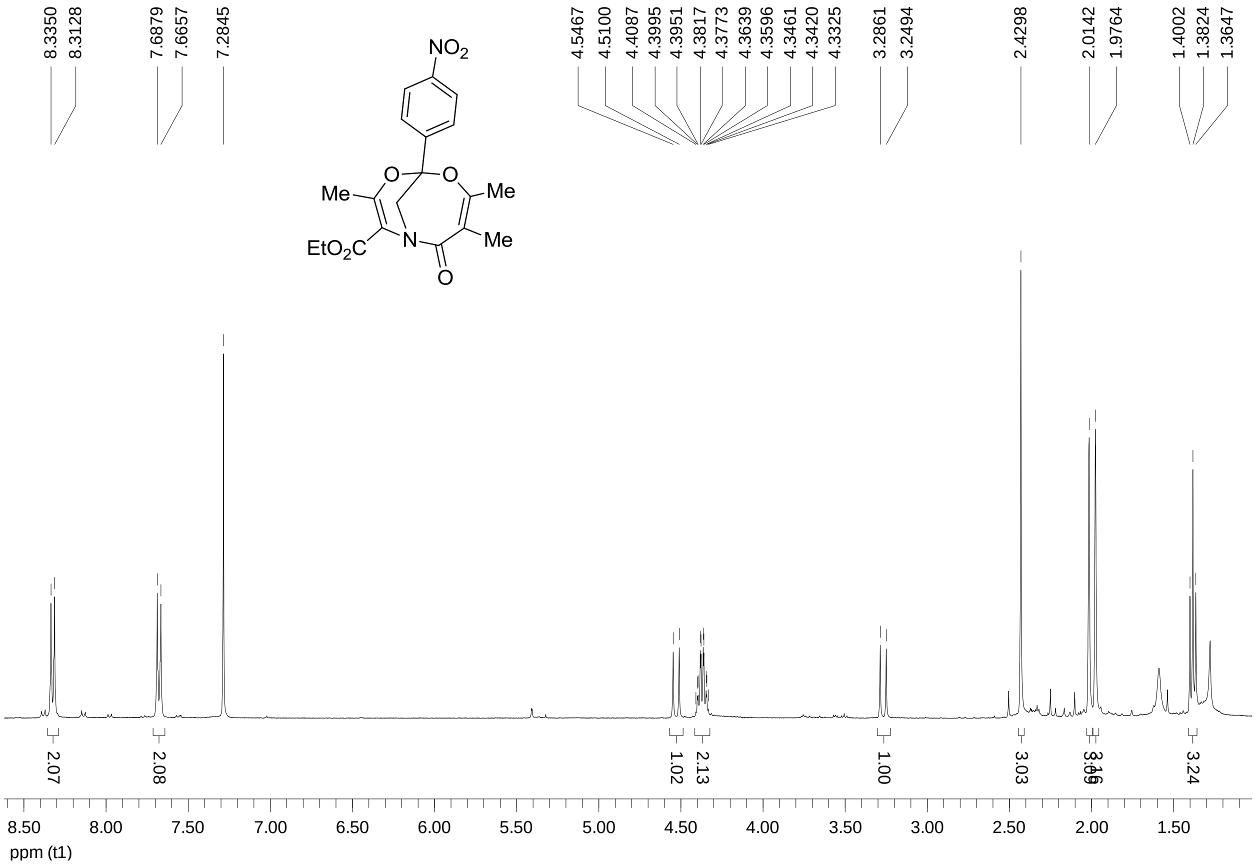

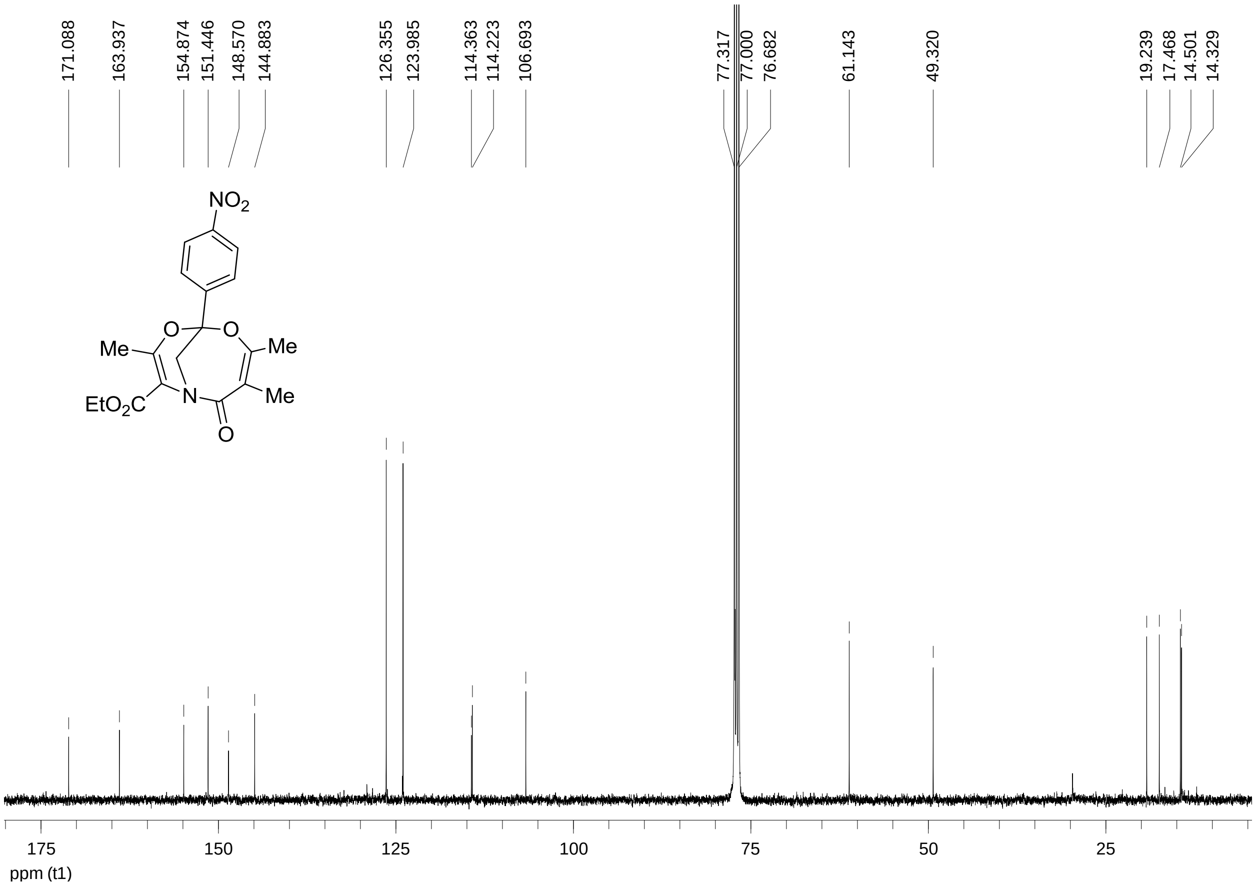

The signals of compounds 4f,l and 6f,l in the reaction mixture

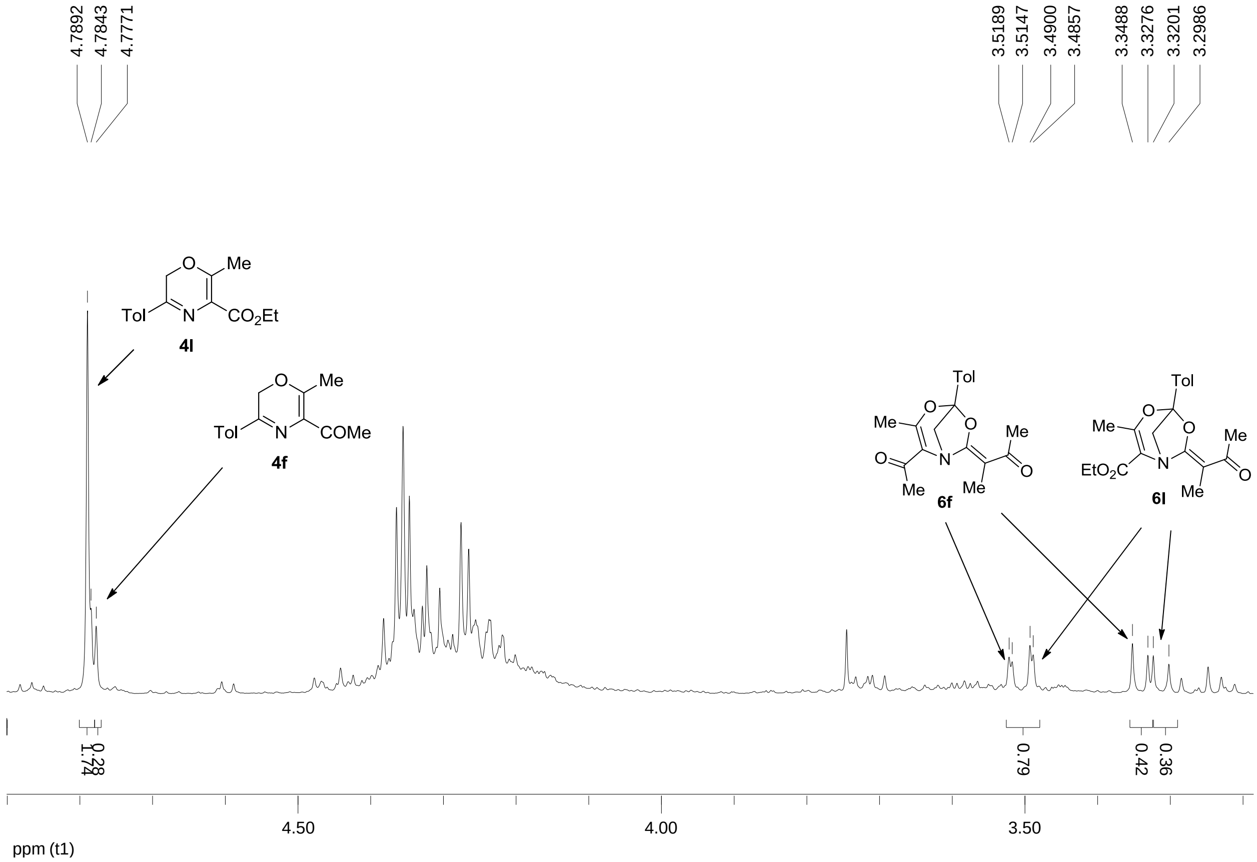

1-(6′-Methyl-3′-phenylspiro[fluorene-9,2′-[1,4]oxazine]-5-yl)ethanone (16)

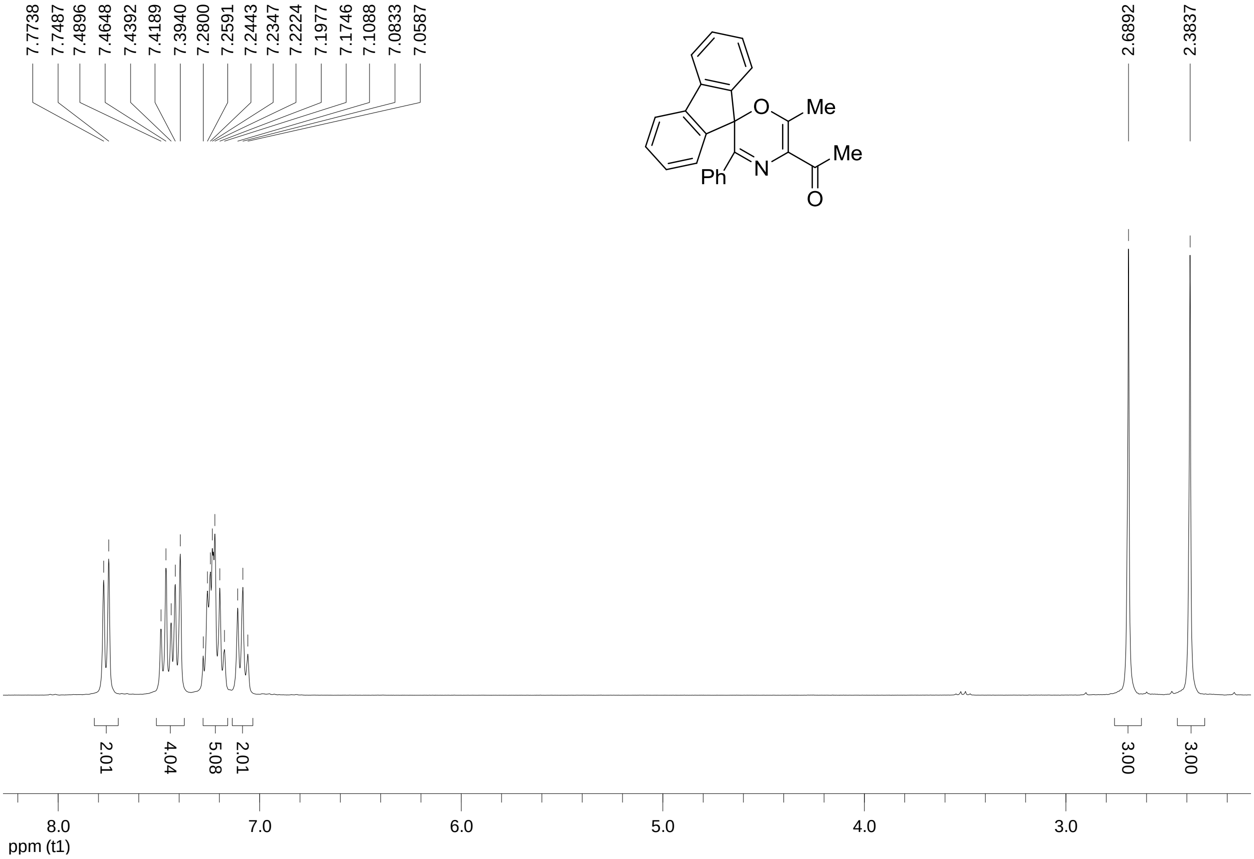

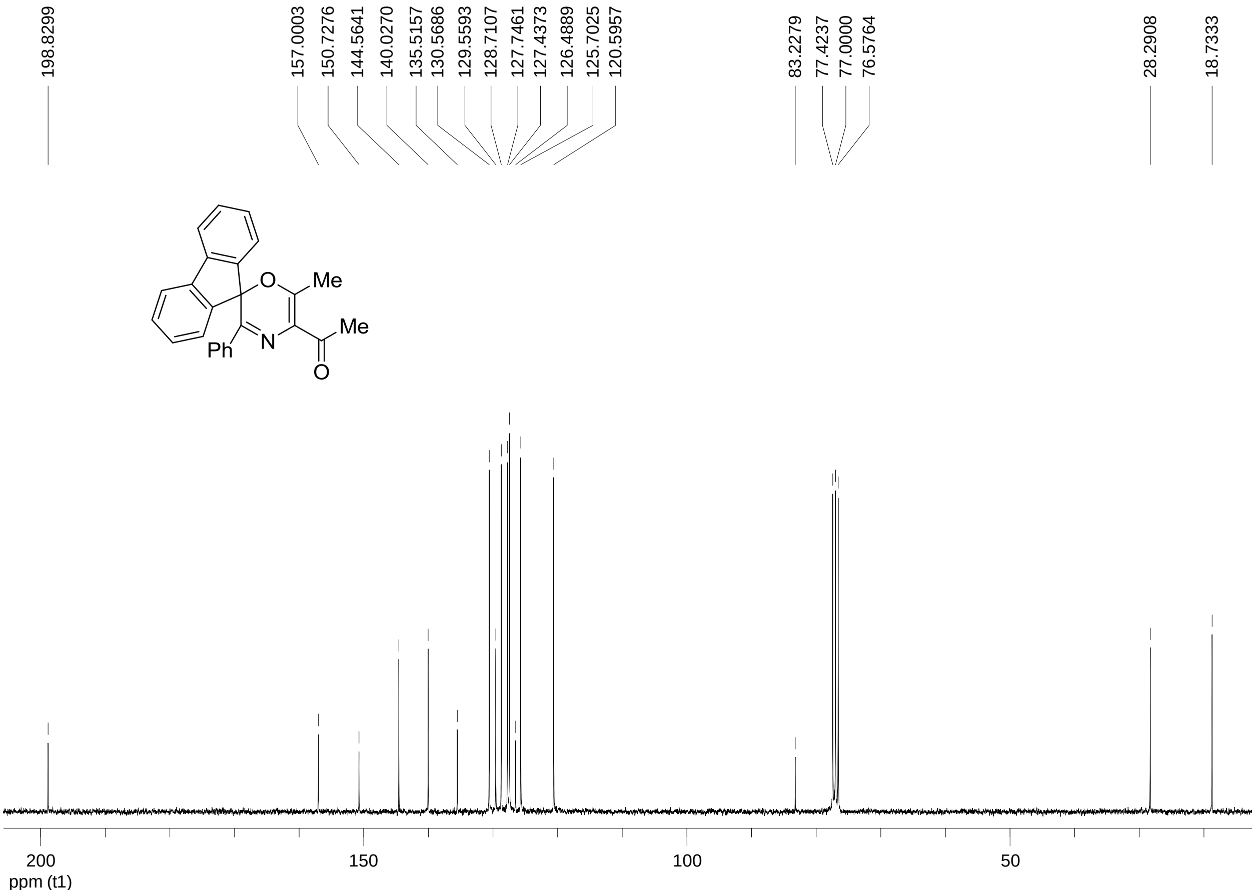

2,5,6-Trimethyl-2- (6′-methyl-3′-phenylspiro[fluorene-9,2′-[1,4]oxazine]-5′-yl)-4*H*-1,3-dioxin-4-one (17)

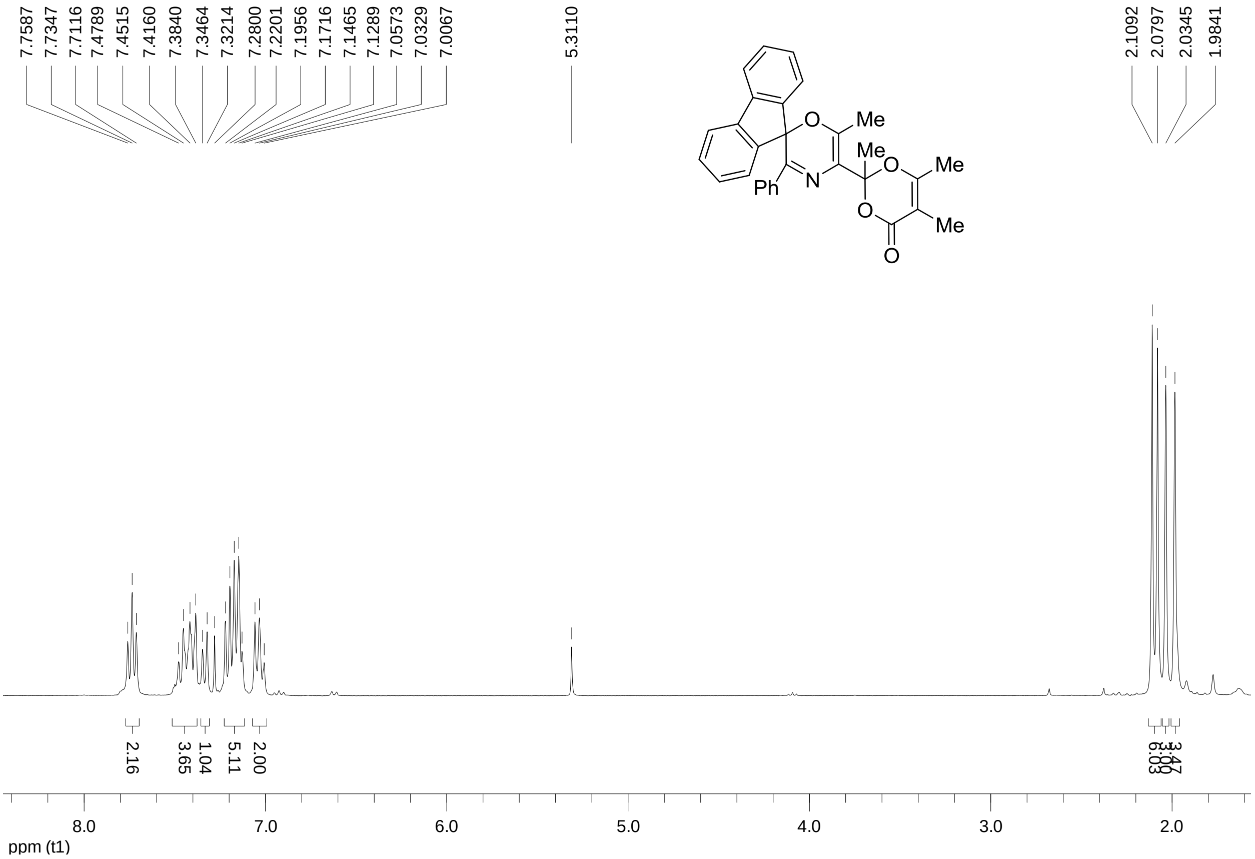

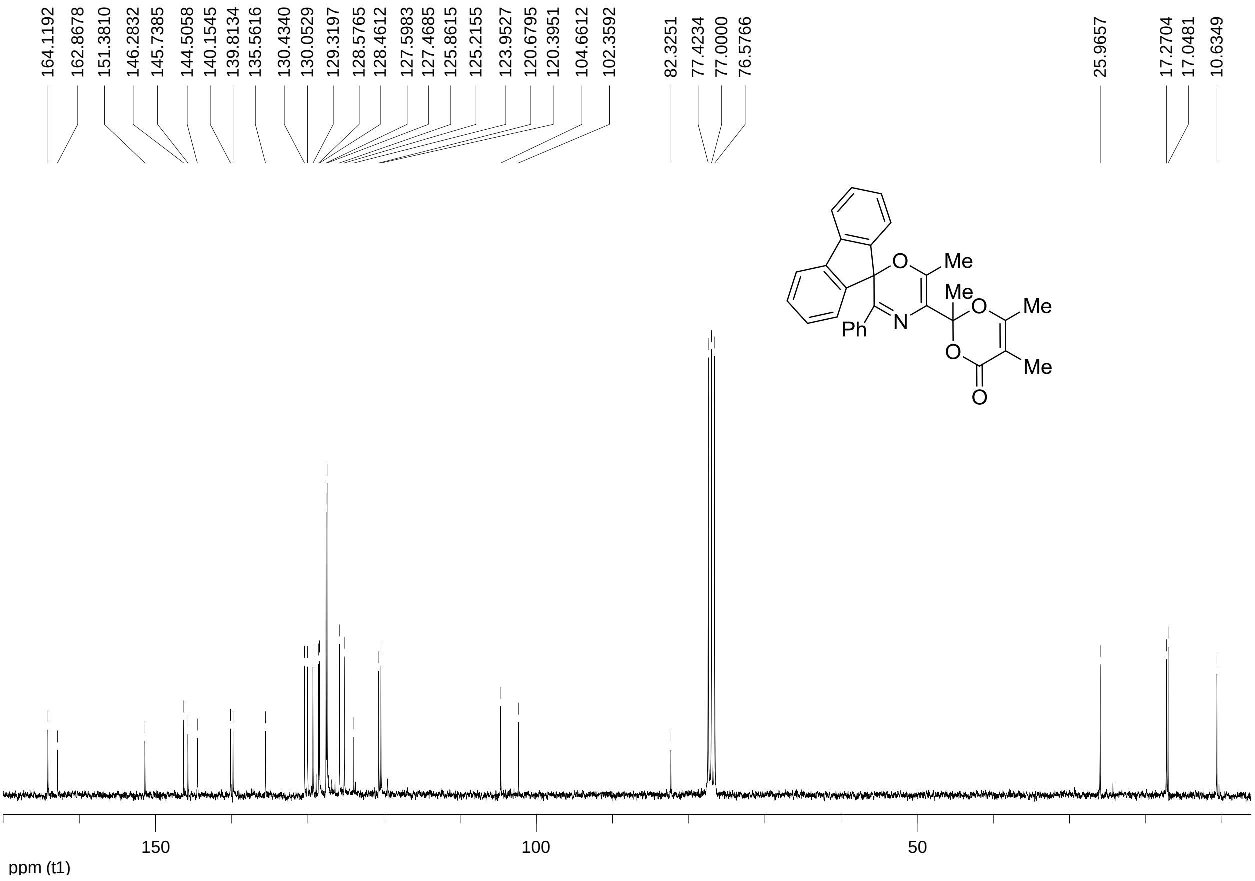

2-{1-[(Fluorene-9-yliden)(phenyl)methylimino]-2-oxopropyl}-2,5,6-trimethyl-4*H*-1,3-dioxin-4-one (18)

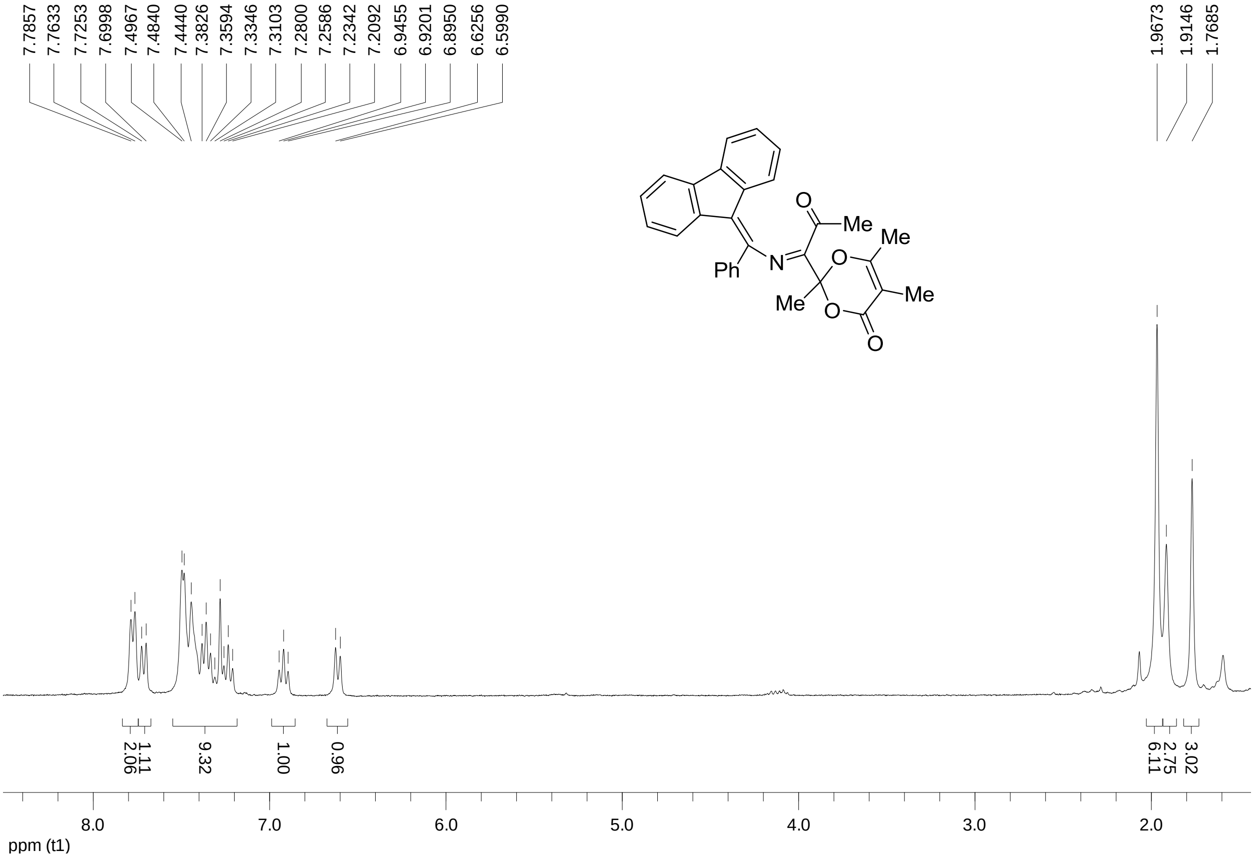

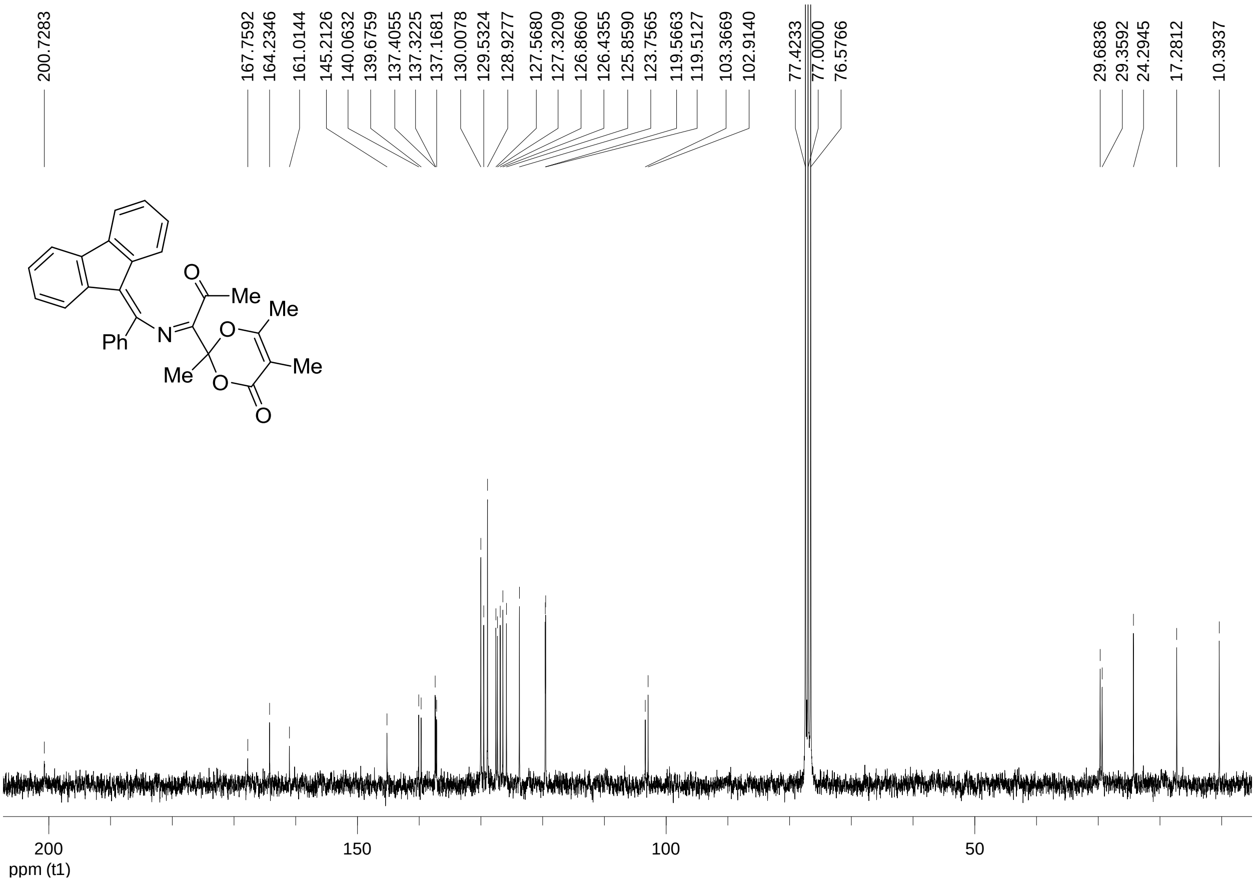

Thermal ellipsoid plot for the crystal structure 17

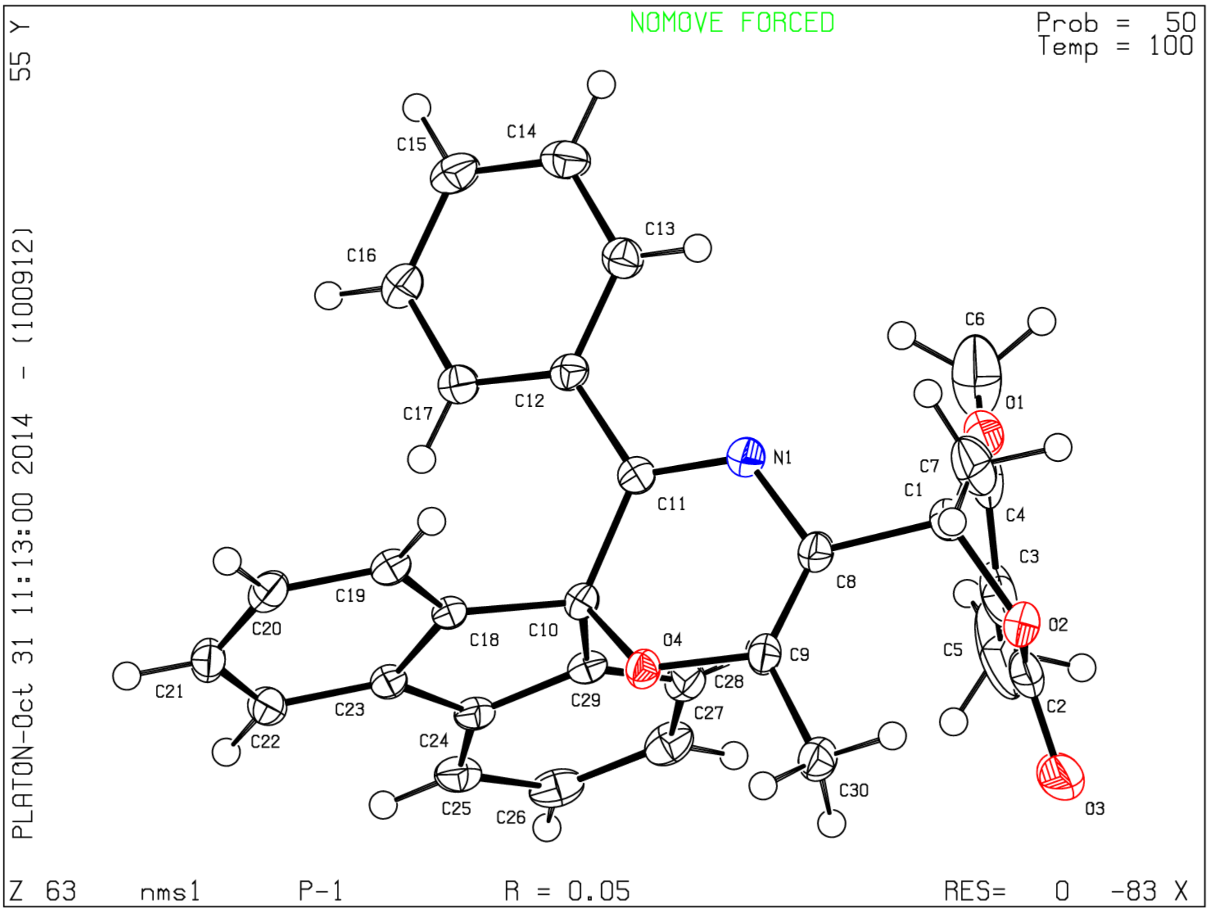

Computational details

All calculations were performed with the density functional method by using the Gaussian 09 suite of quantum chemical programs [13]. Geometry optimizations of intermediates, transition states, reactants, and products in 1,2-dichloroethane were performed at the DFT B3LYP/6-31+G(d,p) level using PCM model. Stationary points on the respective potential-energy surfaces were characterized at the same level of theory by evaluating the corresponding Hessian indices. Careful verification of the unique imaginary frequencies for transition states was carried out to check whether the frequency indeed pertains to the desired reaction coordinate. A transition states search (TS1, TS2, TS10, TS11) was carried out for the most stable W-conformations of the ylides **9j,m**. For locating transition states TS3 and TS5 and estimating reaction barriers two conformations of acetyl(methyl)ketene **12**, conformation 1 (for TS3) and conformation 2 (for TS5) were used. For both modes of attack of dihydroazireno[2,1-*b*]oxazole **10j** on ketene **12** two local minima corresponding to atropoisomeric azireno[2,1-*b*]oxazolium betaines **13j**,**13′j** were found. The calculation of zero point corrected energies E_0 reveals no activation barrier for isomerization of **13j**,**13′j** to oxazinium betaines **14j**,**14′j**.

Table S1: Energies and Cartesian coordinates of compounds **3j, 6j, 7j, 9j, 10j, 13j, 13′j, 14j, 14′j, 15j** and transition states TS1-TS9

Azadiene **3j**

E_0 = -708.196020, E = -708.173894
Imaginary frequency = 0.

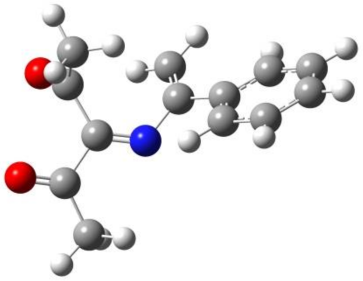

N	0.56337000	0.33537400	-0.72769300
C	1.68956400	0.11179200	-0.16515600
C	-0.46637500	-0.63485700	-0.79351400
C	-0.24445000	-1.84131000	-1.35580900
C	2.73981100	1.20818100	-0.17776100
C	2.09586100	-1.17679700	0.56099100
C	-1.79316000	-0.15235700	-0.32152600
C	-2.99126400	-0.75570700	-0.74808000
C	-4.22299100	-0.30794400	-0.27204200
C	-4.28661100	0.75679100	0.63446100
C	-3.10571300	1.37176100	1.05658800
C	-1.87093000	0.92320300	0.58092300
O	3.73121800	1.07074000	0.52744000
C	2.50171700	2.41742300	-1.03933000
O	2.89120400	-1.92605400	0.01787700
C	1.52006100	-1.41421100	1.92930300
H	0.73139800	-2.11258800	-1.74001700
H	-1.04625600	-2.55750900	-1.48795200
H	-2.96685700	-1.56668900	-1.46820900
H	-5.13572900	-0.78426200	-0.61765100
H	-5.24739000	1.10579900	1.00093900
H	-3.14188200	2.20169000	1.75612500
H	-0.96083900	1.41028600	0.91485900
H	1.53698500	2.87442200	-0.80109500
H	3.31051000	3.13307500	-0.88756300
H	2.45740600	2.12137300	-2.09306900
H	1.91563100	-0.65278200	2.61336700
H	1.80018200	-2.40680900	2.28611700
H	0.43074700	-1.30691600	1.92118400

Ylide **9j**

E_0 = -708.153370, E = -708.132232
Imaginary frequency = 0.

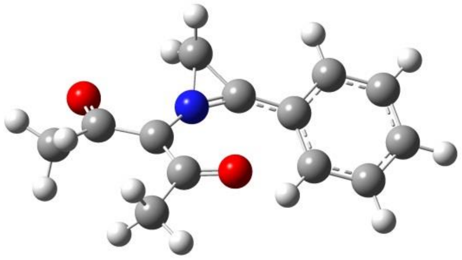

N	0.65553200	-0.77012200	0.10345200
C	1.80682400	-0.00315300	0.02340600
C	-0.61434500	-0.93525600	0.16352300
C	0.19397100	-2.14387000	0.43479000
C	3.00079200	-0.77149500	-0.29227200
C	1.62279900	1.40611000	0.30768600
C	-1.92110600	-0.37087700	0.02257200
C	-3.02098800	-1.19823700	0.34102200
C	-4.31967800	-0.71934600	0.19514000
C	-4.53593800	0.58058900	-0.27825300
C	-3.44908000	1.40128400	-0.60601700
C	-2.14442600	0.93647400	-0.46249700
O	2.90441000	-1.95560400	-0.67161200
C	4.39153900	-0.17436200	-0.15634400
O	0.51260900	1.82295600	0.69805800
C	2.74713800	2.41320800	0.13441300
H	0.35300400	-2.88881300	-0.33923400
H	0.34458200	-2.47150000	1.46374400
H	-2.84376800	-2.20645800	0.70246200
H	-5.16200800	-1.35623000	0.44605100
H	-5.54948700	0.95169000	-0.39676200
H	-3.62173600	2.40659000	-0.97758700
H	-1.29784500	1.56769700	-0.69920400
H	4.62620100	0.46278300	-1.01602500
H	4.51680300	0.42291800	0.74906000
H	5.10349300	-1.00161600	-0.14656100
H	3.44105400	2.37249300	0.98096500
H	2.29704500	3.40740500	0.11461200
H	3.32278400	2.25865900	-0.78026200

Azirenooxazole **10j**

E_0 = -708.175792, E = -708.155691
Imaginary frequency = 0.

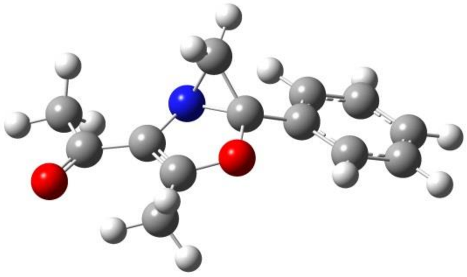

O	0.23722400	1.44507500	0.39612700
C	1.53503900	1.25126000	0.02025900
C	1.85171400	-0.07386200	0.00244200
N	0.70728400	-0.86297400	0.41025900
C	-0.32913600	0.15357500	0.66453000
C	0.27178700	-0.57181300	1.81234600
C	2.30217100	2.47829600	-0.30544400
C	3.09675900	-0.71240700	-0.42395000
O	4.09889200	-0.05463800	-0.73215400
C	3.11196400	-2.22490700	-0.46219000
C	-1.73512900	0.00892800	0.18608400
C	-2.53359200	1.13748400	-0.04540000
C	-3.86037300	0.98509200	-0.45895000
C	-4.39949900	-0.29080400	-0.64490400
C	-3.60280500	-1.41816800	-0.41754700
C	-2.27748300	-1.27045200	-0.00438000
H	-5.43002200	-0.40670600	-0.96720500
H	-0.28957600	-1.37481300	2.27962900
H	0.96356900	-0.02813900	2.45198000
H	1.76723600	3.06560700	-1.05992800
H	2.40370500	3.10432300	0.58863800
H	3.29159900	2.21086600	-0.67248000
H	2.96809500	-2.63134500	0.54558300
H	4.06759900	-2.56731200	-0.86179000
H	2.28690100	-2.60451400	-1.07347200
H	-2.11916700	2.13000400	0.09356000
H	-4.47030800	1.86595800	-0.63676100
H	-4.01084000	-2.41346700	-0.56709100
H	-1.65720400	-2.14647100	0.15835800

[3.2.1] adduct **6j**

E_0 = -1052.702594, E = -1052.672408,
Imaginary frequency = 0.

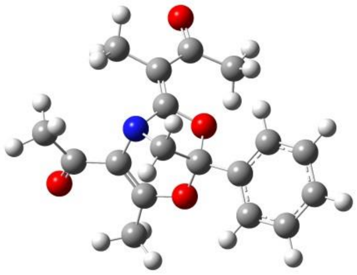

O	-0.67279400	-1.36219500	0.84710700
C	0.59785200	-1.86438600	0.82318600
C	1.51893700	-1.40245500	-0.07676300
N	1.07702600	-0.37776200	-1.03554100
C	-1.02457800	-0.36442600	-0.13155000
C	-0.30818200	-0.69653000	-1.44239700
C	0.76010800	-2.93963000	1.84640600
C	2.87339400	-1.97173800	-0.22135600
O	3.40757700	-2.61156300	0.69001400
C	3.59920600	-1.78346800	-1.53858900
C	-2.52054700	-0.19736800	-0.14922100
C	-3.36127600	-1.26781300	0.18368200
C	-4.74844200	-1.12204300	0.09595000
C	-5.30442700	0.08826200	-0.32706900
C	-4.46650300	1.15595400	-0.66370400
C	-3.07996100	1.01523400	-0.57735500
H	-6.38258000	0.19995800	-0.39227800
C	0.89297100	0.85556700	-0.32276300
O	-0.37459400	0.86440400	0.23446000
C	1.78250500	1.87027900	-0.20596600
C	3.09786500	1.81782400	-0.94625600
C	1.56097200	3.07135400	0.63717200
O	2.44183200	3.93905600	0.67133200
C	0.30363400	3.26502800	1.45899100
H	-0.63712500	-0.02548300	-2.23871200
H	-0.40711700	-1.73513900	-1.75530700
H	1.05453900	-2.49709500	2.80531000
H	-0.19601200	-3.45015700	1.98745400
H	1.53985000	-3.64356600	1.56070200
H	2.92033000	-1.63280300	-2.37911500
H	4.23386600	-2.65654900	-1.70567800
H	4.25127100	-0.90571800	-1.47169200
H	-2.93441500	-2.20723800	0.51588700
H	-5.39228400	-1.95529800	0.36081700
H	-4.89033700	2.10079700	-0.99016700
H	-2.43398500	1.84845100	-0.83217900
H	3.07818700	1.05414700	-1.72286400
H	3.30202700	2.78837100	-1.40609500
H	3.93275000	1.60828300	-0.26713800
H	0.40371600	4.19127400	2.02686200
H	-0.57918600	3.32157000	0.81518600
H	0.13999600	2.42504100	2.14009800

[4.3.1] adduct **7j**

E_0 = -1052.714043, E = -1052.683982
Imaginary frequency = 0.

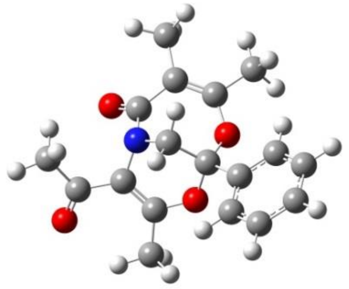

O	-0.31618600	-1.01587200	1.09829800
C	0.89504400	-1.62066300	0.90774500
C	1.80924400	-1.12612300	0.02594600
N	1.39577300	0.07399900	-0.65245300
C	-0.85314200	-0.01381200	0.19048600
C	0.00224300	0.02498400	-1.06592000
C	1.00757800	-2.84419700	1.75674800
C	3.09192400	-1.76173600	-0.34137200
O	3.63386500	-2.61528600	0.36643600
C	3.71905500	-1.33180600	-1.65116600
C	-2.30659600	-0.39725300	-0.06427700
C	-2.62407800	-1.72742100	-0.37996800
C	-3.93763600	-2.08838600	-0.68369800
C	-4.95116300	-1.12422900	-0.67608200
C	-4.64061500	0.20042800	-0.36153600
C	-3.32413300	0.56375400	-0.05940100
H	-5.97366500	-1.40548400	-0.90981600
C	1.95397600	1.27513400	-0.22695400
O	3.14713300	1.34036300	0.08368700
C	1.07058400	2.45954400	-0.11219600
C	1.71426800	3.79674900	-0.43025000
C	-0.14882800	2.38073700	0.47986800
O	-0.81849300	1.23469900	0.87545500
C	-0.95563500	3.57293900	0.90921300
H	-0.25988300	0.88761200	-1.67883600
H	-0.15712300	-0.87916200	-1.65660300
H	1.16528600	-2.54704900	2.80061000
H	0.07332500	-3.41167300	1.71221500
H	1.85194000	-3.45696100	1.44863600
H	2.98808300	-1.35615700	-2.46600900
H	4.55623000	-1.99379800	-1.87737100
H	4.07647800	-0.30110400	-1.56407400
H	-1.84944100	-2.48729500	-0.37718800
H	-4.16900800	-3.12228500	-0.92175500
H	-5.42095300	0.95546900	-0.34713100
H	-3.09405400	1.59152800	0.19219200
H	2.56161800	3.64917600	-1.10303200
H	1.01070700	4.47573700	-0.91843100
H	2.09781100	4.29381200	0.46854200
H	-1.35861700	3.39651500	1.91105200
H	-0.36466600	4.48757800	0.92562400
H	-1.80757800	3.72243900	0.23442400

Ketene **12** (conformation 1)

E_0 = -344.500317, E = -344.489495
Imaginary frequency = 0.

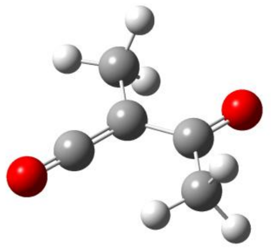

C	-1.39841400	-0.27906700	-0.00196800
O	-2.37458800	-0.91871800	-0.00417600
C	-0.29848600	0.47105400	0.00000900
C	-0.41949900	1.98539100	0.00603100
C	1.02798200	-0.16338600	-0.00389300
O	2.03705400	0.54529000	-0.00817000
C	1.15219200	-1.67327200	0.00625700
H	0.07469600	2.39260800	0.89289200
H	0.07177900	2.39939400	-0.87928500
H	-1.46250900	2.30828200	0.00896800
H	1.78244500	-1.98260900	-0.83264400
H	1.65643000	-1.97879200	0.92869400
H	0.19477700	-2.19578300	-0.05846500

Ketene **12** (conformation 2)

E_0 = -344.497903, E = -344.487303
Imaginary frequency = 0.

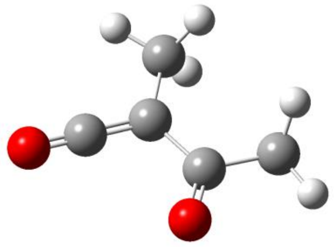

C	-1.48131300	-0.10349000	0.00000900
O	-2.54538200	-0.57611200	-0.00011900
C	-0.26324800	0.44129000	0.00004500
C	-0.10477800	1.95390200	0.00005500
C	0.85083100	-0.52287700	-0.00014300
O	0.65727600	-1.74411800	0.00017800
C	2.24980800	0.05285800	-0.00022100
H	-1.07651200	2.45260200	0.00444800
H	0.44424100	2.28706800	0.88624400
H	0.43704100	2.28823400	-0.89015600
H	2.97287000	-0.76355800	-0.00586300
H	2.40817000	0.68878300	-0.87826500
H	2.41123600	0.67861300	0.88465200

Betaine **13j**

E_0 = -1052.659509, E = -1052.628396
Imaginary frequency = 0.

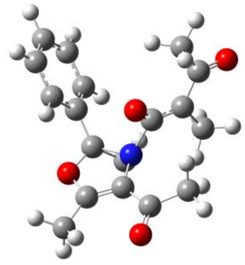

O	-0.95144600	-2.35150800	0.42327700
C	-2.14901400	-1.81500200	0.05425900
C	-2.12889200	-0.45389200	0.03627600
N	-0.81930500	0.00910000	0.49633700
C	-0.04598800	-1.34357500	0.78625700
C	-0.44583300	-0.43954700	1.87723200
C	-3.20597300	-2.80719500	-0.25512300
C	-3.23973700	0.43205200	-0.36886300
O	-4.33272100	-0.06552600	-0.64435300
C	-3.03281700	1.92470500	-0.44122500
C	1.34654300	-1.60842700	0.34923500
C	1.57354300	-2.21243700	-0.89847300
C	2.87535100	-2.49341700	-1.30932400
C	3.95750800	-2.17511800	-0.48099400
C	3.73461400	-1.57986400	0.76329400
C	2.43218000	-1.29797600	1.18048000
H	4.97073800	-2.39434200	-0.80384100
C	-0.02224900	1.11489600	-0.37163900
O	0.03128900	0.75472500	-1.54550100
C	0.44506700	2.20979000	0.34736500
C	-0.01909200	2.60480900	1.73425900
C	1.39273600	3.12122500	-0.27858400
O	1.80391600	4.12171800	0.34839400
C	1.92004800	2.88391900	-1.68374100
H	0.32988500	0.16220200	2.33026200
H	-1.28581100	-0.69119900	2.51658700
H	-3.94609300	-2.37633300	-0.92655800
H	-2.74960500	-3.69745200	-0.69564900
H	-3.71582900	-3.10835100	0.66791600
H	-2.64105500	2.32354600	0.49851100
H	-3.99156600	2.39346300	-0.66500500
H	-2.31409900	2.17262900	-1.22856300
H	0.73540400	-2.45703700	-1.54114500
H	3.04596400	-2.95724700	-2.27566800
H	4.57022900	-1.33997000	1.41290800
H	2.27249000	-0.85219500	2.15628300
H	0.74217200	2.43444900	2.50778800
H	-0.22322900	3.67970800	1.74564300
H	-0.93516700	2.09779800	2.04179500
H	2.71122900	3.60995300	-1.88180400
H	2.30259800	1.86720900	-1.81026600
H	1.12061800	3.00453500	-2.42224000

Betaine **13′j**

E_0 = -1052.661966, E = -1052.630505
Imaginary frequency = 0.

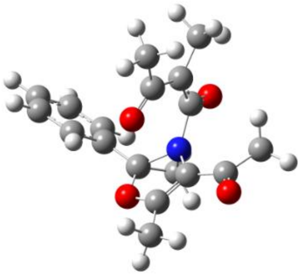

O	-0.19062900	-2.18755100	0.37178500
C	1.15660000	-2.03903500	0.53156700
C	1.64980600	-0.96824000	-0.14319300
N	0.55088200	-0.34044900	-0.87119300
C	-0.68600300	-1.25237600	-0.55187100
C	-0.06987400	-1.23844500	-1.89508000
C	1.80442700	-3.05570200	1.39154200
C	3.05535200	-0.55447900	-0.27206500
O	3.90360100	-1.01845400	0.49327400
C	3.45302900	0.40236300	-1.37481100
C	-2.05339400	-0.75714800	-0.24630900
C	-2.45471500	-0.59227800	1.08947400
C	-3.75297200	-0.17496800	1.37943000
C	-4.65886700	0.08319300	0.34475200
C	-4.26485500	-0.08581400	-0.98498700
C	-2.96836700	-0.51113400	-1.28123200
H	-5.66838300	0.41061800	0.57476800
C	0.43500700	1.20533300	-1.12483800
O	0.27030800	1.46061000	-2.32370600
C	0.58595000	2.06274600	-0.03117500
C	0.69622000	3.53697600	-0.38856400
C	0.55035100	1.67175500	1.36230900
O	0.30952300	0.51694200	1.77557700
C	0.79401200	2.76389900	2.39866500
H	-0.56470900	-0.67392800	-2.67521600
H	0.55992700	-2.05960300	-2.21825200
H	1.51635400	-2.88223300	2.43528000
H	1.46313600	-4.05646200	1.11089400
H	2.88733800	-2.98101100	1.31322600
H	2.84000500	0.29451900	-2.27281100
H	4.50405300	0.23096200	-1.61418200
H	3.34286900	1.43277600	-1.02010800
H	-1.74330000	-0.77295800	1.88480000
H	-4.05644700	-0.04689000	2.41389400
H	-4.96512600	0.10366200	-1.79228800
H	-2.68461900	-0.65735300	-2.31747400
H	0.82220600	3.65680000	-1.46582700
H	-0.19749900	4.10574500	-0.09867200
H	1.55241600	4.01153000	0.10391300
H	0.73456200	2.32223500	3.39483700
H	1.77824200	3.22802900	2.26845100
H	0.05013100	3.56445600	2.31600800

Betaine **14j**

E_0 = -1052.676669, E = -1052.645098
Imaginary frequency = 0.

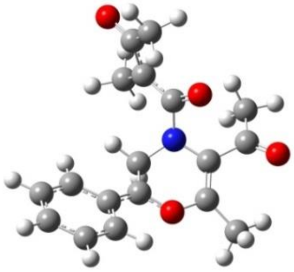

O	-1.18083400	-1.98460200	-0.13852600
C	0.08979100	-2.56252700	-0.10835900
C	1.15020500	-1.70553100	-0.13910300
N	0.93661700	-0.38509800	-0.51711100
C	-1.38412000	-0.76369100	-0.56845300
C	-0.25063400	-0.17389500	-1.34532000
C	0.00909400	-4.04177000	0.04189000
C	2.55300700	-2.17551600	0.16610900
O	2.73218900	-3.12071200	0.92256500
C	3.69673300	-1.49395100	-0.54581200
C	-2.62780900	-0.14465800	-0.22207300
C	-3.55397100	-0.81782800	0.61761300
C	-4.77285100	-0.22976700	0.91831800
C	-5.09121200	1.03499700	0.40015100
C	-4.18490700	1.71253200	-0.42353000
C	-2.96021100	1.13398200	-0.73508100
H	-6.04481400	1.49281600	0.64366300
C	1.52970900	0.68469100	0.29701000
O	1.85704800	0.33446400	1.45334100
C	1.69113200	1.94025700	-0.33626000
C	1.76888400	2.09628100	-1.84297900
C	1.90164000	3.14270800	0.43087300
O	2.11114800	4.23936900	-0.14870600
C	1.84878400	3.15076500	1.95211200
H	-0.38898800	0.88680900	-1.52068800
H	-0.15175500	-0.69098000	-2.31090500
H	1.00011700	-4.48655500	-0.01603700
H	-0.42889300	-4.30829500	1.01031000
H	-0.62579000	-4.46446200	-0.74407100
H	3.49544500	-1.41658800	-1.61914800
H	4.60881100	-2.06695500	-0.37433800
H	3.83439900	-0.47847200	-0.16328500
H	-3.30132200	-1.79069000	1.02237900
H	-5.47762500	-0.74667500	1.56089900
H	-4.43322900	2.69156500	-0.81870900
H	-2.27002300	1.67137600	-1.37386200
H	0.93103800	2.66835000	-2.26662200
H	2.67250100	2.65867600	-2.10370100
H	1.82499200	1.13434300	-2.35819600
H	1.65923700	4.17542300	2.28249600
H	1.09332200	2.47227100	2.35440300
H	2.81025800	2.82311300	2.36314800

Betaine **14′j**

E_0 = -1052.678213, E = -1052.646900
Imaginary frequency = 0.

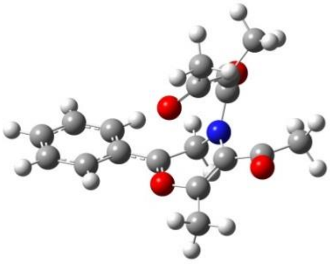

O	0.96357200	1.63753100	-0.00381700
C	-0.37872100	2.00622300	-0.08845500
C	-1.24784400	1.04856700	-0.52490200
N	-0.71013500	-0.09653700	-1.08954200
C	1.45705600	0.61381400	-0.65033700
C	0.58034700	0.07484500	-1.73919700
C	-0.59907700	3.39406600	0.40289500
C	-2.73971100	1.21055700	-0.45281400
O	-3.24722900	1.89677700	0.43187700
C	-3.58604800	0.65970300	-1.58380400
C	2.75183400	0.14448200	-0.24810100
C	3.40630300	0.71617600	0.87245400
C	4.67594800	0.28600600	1.22946900
C	5.31258600	-0.72050200	0.48830300
C	4.67462700	-1.29704000	-0.61601700
C	3.40335000	-0.87285400	-0.98600900
H	6.30352600	-1.05783500	0.77593300
C	-1.26133800	-1.40015400	-0.87154200
O	-1.08727200	-2.24584900	-1.77395400
C	-2.02916200	-1.56369700	0.32421500
C	-3.11524700	-2.61839400	0.28545300
C	-1.57936700	-1.03659000	1.59195100
O	-0.50142200	-0.41575700	1.76642300
C	-2.44841400	-1.31513900	2.81437300
H	0.90761300	-0.88364100	-2.13180900
H	0.51767200	0.80414800	-2.56015000
H	-0.34451100	3.46993900	1.46614100
H	0.03324800	4.09967900	-0.14693800
H	-1.64357800	3.67487800	0.28329200
H	-3.85686000	1.50827200	-2.22509100
H	-4.51040300	0.24287600	-1.17782200
H	-3.07836800	-0.08417500	-2.19680700
H	2.90714300	1.48532700	1.44982500
H	5.17221600	0.72459300	2.08878900
H	5.16784100	-2.07808800	-1.18472100
H	2.92156200	-1.32949500	-1.84223200
H	-3.38935800	-2.84006200	-0.74906500
H	-2.80763800	-3.56891000	0.74594400
H	-4.01880800	-2.28886700	0.81077700
H	-2.01673100	-0.80812500	3.67932100
H	-3.47667500	-0.96660400	2.66551300
H	-2.50087300	-2.39018900	3.02404600

Carbonyl ylide **15j**

E_0 = -708.163881, E = -708.143511
Imaginary frequency = 0.

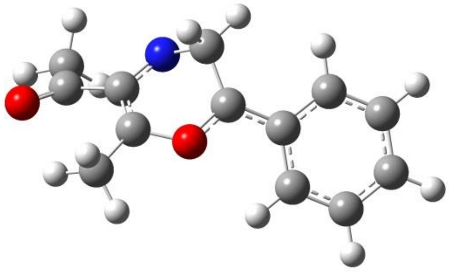

O	-0.15704500	1.08066800	-0.46527900
C	-1.52899100	1.03081400	-0.44994800
C	-2.02415300	-0.25417600	-0.17654900
N	-1.25759300	-1.33897600	-0.34402800
C	0.54823900	-0.02755600	-0.67497200
C	-0.22147700	-1.09424300	-1.34654000
C	-2.16532900	2.37769600	-0.43008000
C	-3.35160100	-0.45168400	0.49580300
O	-4.27670900	0.34081400	0.33353900
C	-3.51418700	-1.68674100	1.35165900
C	1.90903500	-0.03336200	-0.18633800
C	2.39385800	1.02311200	0.62063400
C	3.71923500	1.03625600	1.04457500
C	4.58587700	-0.00610000	0.69206900
C	4.11534500	-1.06249900	-0.09637700
C	2.79401100	-1.07888600	-0.53579100
H	5.61687100	0.00310100	1.03211000
H	0.35588700	-1.99825700	-1.52990500
H	-0.64765600	-0.72573200	-2.29978800
H	-1.49741300	3.10202600	0.04829900
H	-2.37784300	2.73832000	-1.44484400
H	-3.11429300	2.33946200	0.10582700
H	-3.53892400	-2.57634500	0.71405900
H	-4.44860500	-1.61188500	1.91027200
H	-2.66617300	-1.80915900	2.03115600
H	1.72342300	1.82445700	0.91010300
H	4.07677100	1.85557000	1.66088400
H	4.78121100	-1.87428500	-0.37262200
H	2.45074300	-1.90208000	-1.15230400

TS1

E_0 = -708.137105, E = -708.116078,
Imaginary frequency = 1.

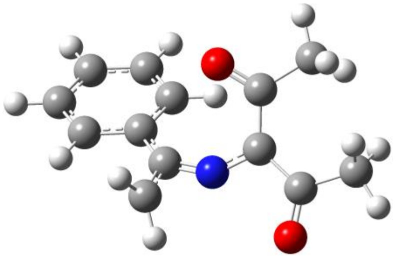

N	0.71279300	-0.98510800	-0.02323100
C	1.70281100	-0.07316200	-0.01612400
C	-0.54744500	-1.15608500	0.18509300
C	-0.16939200	-2.33110000	0.91985800
C	2.98521200	-0.60885400	-0.51471000
C	1.41704900	1.21657600	0.63802100
C	-1.78582100	-0.44514200	-0.04907300
C	-2.98354300	-1.03905300	0.39303300
C	-4.20343000	-0.40519400	0.16742200
C	-4.23994000	0.80831000	-0.52956200
C	-3.05407500	1.39045300	-0.99480400
C	-1.82863100	0.77477700	-0.75212500
O	3.04109100	-1.77343000	-0.93707300
C	4.25620500	0.22009800	-0.52956400
O	0.34818800	1.36420100	1.25028700
C	2.38562500	2.38479000	0.58799800
H	0.29019500	-3.17069200	0.41971800
H	-0.34752400	-2.38949000	1.99326200
H	-2.95002400	-1.99668600	0.90446700
H	-5.12376800	-0.86010200	0.51982500
H	-5.19170900	1.29638000	-0.71677600
H	-3.08711600	2.32785700	-1.54115700
H	-0.91036000	1.23099100	-1.10101000
H	4.15575300	1.10396400	-1.16594700
H	4.53135200	0.56226200	0.47237900
H	5.05770300	-0.40658700	-0.92289000
H	3.26758200	2.19755600	1.20784300
H	1.86733800	3.26022600	0.98235400
H	2.72904300	2.59542800	-0.42793700

TS2

E_0 = -708.139841, E = -708.119688,
Imaginary frequency = 1.

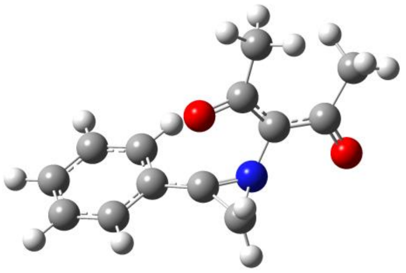

N	-0.70704400	-1.10547500	-0.11447500
C	-1.74672500	-0.13244700	0.02570400
C	0.50527800	-0.80613300	-0.53388000
C	-0.21307500	-1.76222800	-1.38474400
C	-2.93829500	-0.55885200	0.71078500
C	-1.40833000	1.04302200	-0.70000400
C	1.77856600	-0.32824500	-0.06989200
C	2.93053800	-0.60817600	-0.82713000
C	4.17630100	-0.17261100	-0.37859100
C	4.27591800	0.53363500	0.82519300
C	3.13004000	0.80930500	1.58454200
C	1.88138600	0.38631900	1.14025400
O	-3.01891900	-1.70819200	1.19305900
C	-4.12656200	0.37797600	0.87161100
O	-0.26768600	1.10747400	-1.25910500
C	-2.33031200	2.23228700	-0.88109700
H	-0.06041600	-2.83272300	-1.28893400
H	-0.58296500	-1.39448600	-2.34005100
H	2.84023100	-1.16466300	-1.75473200
H	5.06678500	-0.38584800	-0.96107800
H	5.24754000	0.86841200	1.17574300
H	3.21559400	1.35474700	2.51891600
H	0.98853600	0.59685900	1.71997900
H	-3.84017700	1.34653700	1.28997700
H	-4.61917400	0.56271500	-0.08907300
H	-4.84266700	-0.10189400	1.54059900
H	-3.29844700	1.93965600	-1.29340200
H	-1.85162100	2.93875500	-1.55991000
H	-2.50943100	2.73290800	0.07514100

TS3

E_0 = -1052.659509, E = -1052.628396
Imaginary frequency = 1.

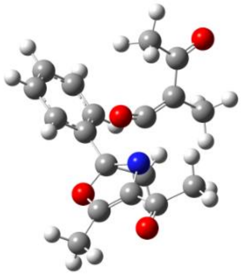

O	-1.27167600	-2.27385800	0.22462100
C	-2.38158100	-1.56832800	-0.13106700
C	-2.21157600	-0.22898200	0.04116100
N	-0.88262000	0.02007600	0.58709700
C	-0.29214000	-1.37877500	0.72975400
C	-0.64949300	-0.60308100	1.93401700
C	-3.52357700	-2.38408600	-0.60738200
C	-3.19341200	0.83878700	-0.20826100
O	-4.25390000	0.59030500	-0.78663600
C	-2.89156200	2.23036700	0.29648600
C	1.09444900	-1.74865900	0.32699100
C	1.36104900	-2.09761400	-1.00636400
C	2.64952800	-2.47278000	-1.38548100
C	3.67875800	-2.50777200	-0.43794800
C	3.41427800	-2.17275200	0.89240100
C	2.12443900	-1.79701900	1.27564000
H	4.68131900	-2.79991200	-0.73566200
C	0.16643300	1.21436900	-0.44879400
O	-0.03007200	0.86353200	-1.57933700
C	0.85282400	2.11287000	0.32763200
C	0.63112500	2.36819500	1.79937000
C	1.86801500	2.93136200	-0.33948500
O	2.52280600	3.75959200	0.31988200
C	2.14571800	2.78685600	-1.82612800
H	0.15215100	-0.14716800	2.49984200
H	-1.54630100	-0.85394300	2.49174100
H	-4.39277000	-1.75257800	-0.77772200
H	-3.25090600	-2.88238800	-1.54495400
H	-3.75338400	-3.16305300	0.12671300
H	-2.67271400	2.21763000	1.36961900
H	-3.75481000	2.86902500	0.10650900
H	-2.01505400	2.65000100	-0.20752400
H	0.56450300	-2.07022400	-1.74170700
H	2.85082100	-2.73622600	-2.41921500
H	4.20653500	-2.20835400	1.63356300
H	1.92552400	-1.55385200	2.31425600
H	1.38521100	1.88170200	2.43173800
H	0.72066400	3.44152500	1.98517800
H	-0.35763000	2.05390800	2.13204100
H	2.97594300	3.44491600	-2.08760700
H	2.39664300	1.75532000	-2.09167500
H	1.26567600	3.05881900	-2.41833100

TS4

E_0 = -1052.667913, E = -1052.637665
Imaginary frequency = 1.

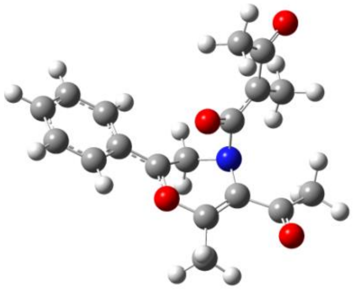

O	-0.98212100	-1.88044900	0.20218800
C	0.32736900	-2.39604600	0.24789700
C	1.34938600	-1.60293700	-0.16164200
N	1.07701200	-0.37118600	-0.84029000
C	-1.26168900	-0.79741800	-0.48521300
C	-0.22998100	-0.43183600	-1.50606500
C	0.30135100	-3.77977500	0.79746800
C	2.77766900	-2.00707800	0.03735000
O	3.07959100	-2.87240800	0.85644800
C	3.83078700	-1.32721100	-0.80153200
C	-2.57845900	-0.24643300	-0.31681100
C	-3.47552400	-0.81621000	0.61982100
C	-4.75416500	-0.29580700	0.76261500
C	-5.15957800	0.79666800	-0.01753800
C	-4.28118500	1.36937700	-0.94468900
C	-2.99791400	0.85664000	-1.09664000
H	-6.15954600	1.20240100	0.10042500
C	0.99913500	0.79665400	0.10271800
O	0.11703300	0.64639000	0.99849600
C	1.81059000	1.90042400	-0.17317100
C	2.72572900	1.96720500	-1.37422900
C	1.79999100	3.08657200	0.67512600
O	2.52566400	4.06346600	0.38597400
C	0.93274400	3.18111900	1.91762200
H	-0.43450200	0.51924100	-1.99362800
H	-0.21760000	-1.22126600	-2.26959600
H	0.13546600	-3.75631200	1.88100700
H	-0.52338700	-4.33879900	0.34370500
H	1.24498000	-4.28840400	0.61441500
H	4.77541900	-1.85834900	-0.67757400
H	3.95641900	-0.29069300	-0.47372100
H	3.54102400	-1.29448400	-1.85520900
H	-3.15827900	-1.65912400	1.22206400
H	-5.43838300	-0.73449000	1.48125000
H	-4.59631100	2.21735800	-1.54322600
H	-2.32523900	1.31896600	-1.80867900
H	2.57497600	1.11460500	-2.03590700
H	2.54204000	2.89010200	-1.93583700
H	3.78552800	1.99781000	-1.08733600
H	1.12342300	4.14319500	2.39861100
H	-0.12772200	3.09263600	1.66360200
H	1.14374000	2.36445800	2.61429900

TS5

E_0 = -1052.656066, E = -1052.624459
Imaginary frequency = 1.

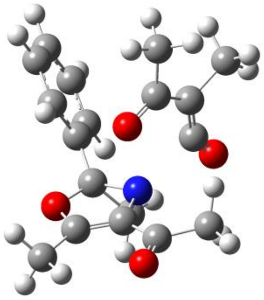

O	-0.50688000	-2.38585100	-0.26748900
C	-1.77872300	-1.92997100	-0.47000600
C	-1.98142100	-0.72503600	0.12530800
N	-0.76946900	-0.32509900	0.82109100
C	0.17251000	-1.45870500	0.58001800
C	-0.40429200	-1.26015200	1.92986000
C	-2.64685500	-2.81361700	-1.28524200
C	-3.19693800	0.09277800	0.12305000
O	-4.20894100	-0.26265300	-0.49180600
C	-3.18703100	1.37629500	0.91983700
C	1.61048800	-1.29480600	0.21489300
C	1.97054500	-1.11864400	-1.13023000
C	3.31356300	-0.98888400	-1.48447300
C	4.30835100	-1.03516800	-0.50073100
C	3.95430300	-1.21706300	0.83846400
C	2.60926900	-1.35123400	1.19505200
H	5.35343100	-0.93465900	-0.77850100
C	0.12505700	1.62033500	1.27275100
O	0.18626400	1.51671200	2.45105000
C	0.39652500	2.35128800	0.15803900
C	1.15314400	3.64400100	0.49013200
C	0.05738000	2.03536800	-1.22547200
O	-0.61173900	1.05701900	-1.58443200
C	0.56801900	3.00577500	-2.28150500
H	0.20172000	-0.79636900	2.70014400
H	-1.18465000	-1.93256600	2.27269400
H	-2.19553000	-2.96504500	-2.27259500
H	-2.73425900	-3.79632000	-0.80860500
H	-3.63287800	-2.36784100	-1.39811700
H	-2.88942300	1.19434800	1.95819400
H	-4.18263000	1.82096300	0.89313000
H	-2.46174800	2.07719700	0.49410600
H	1.19849000	-1.07647500	-1.89057700
H	3.58431800	-0.85023700	-2.52691900
H	4.72140500	-1.26305700	1.60546700
H	2.34392600	-1.50783000	2.23562900
H	0.58946900	4.52747100	0.17162700
H	1.32638200	3.73396700	1.56397300
H	2.13020300	3.66796200	-0.00416700
H	0.25301500	2.65001900	-3.26335500
H	0.17140200	4.01431500	-2.12018000
H	1.66064800	3.08268500	-2.25663500

TS6

E_0 = -1052.675007, E = -1052.644646
Imaginary frequency = 1.

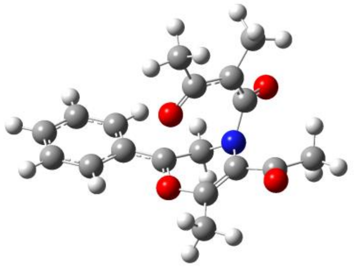

O	-0.70361800	-1.73533400	0.09388000
C	0.66626400	-2.01146200	0.19841700
C	1.55450000	-1.10026200	-0.28825000
N	1.13012800	-0.07513000	-1.14051900
C	-1.11771300	-0.66632900	-0.54340700
C	-0.21492700	-0.23184200	-1.66724300
C	0.87633100	-3.32040300	0.87996300
C	3.02915000	-1.25949000	-0.05584600
O	3.44650500	-1.82139600	0.95316900
C	3.97660700	-0.75175700	-1.12000700
C	-2.50559100	-0.30404600	-0.36261000
C	-3.28979700	-0.97021700	0.60656800
C	-4.62943500	-0.63837300	0.76756500
C	-5.20600800	0.36300500	-0.02529900
C	-4.43684600	1.03310500	-0.98272600
C	-3.09473900	0.70539600	-1.15386700
H	-6.25216500	0.62191700	0.10590300
C	1.58091700	1.30279100	-0.95823900
O	1.93561600	1.90404000	-1.98791300
C	1.58262200	1.84518200	0.36210500
C	2.40567100	3.09937800	0.57880500
C	0.62325300	1.44470100	1.33158300
O	-0.26253800	0.53978300	1.18384500
C	0.57813900	2.19054600	2.65910800
H	-0.53043500	0.69595700	-2.13685700
H	-0.24881200	-1.03168400	-2.42425700
H	0.62581000	-3.24704100	1.94467400
H	0.22315500	-4.07832500	0.43396700
H	1.91287200	-3.64068500	0.79926800
H	3.64794200	-1.06462000	-2.11556500
H	4.97559200	-1.13546700	-0.90815900
H	4.01033600	0.34204600	-1.12896500
H	-2.83972700	-1.73955900	1.22258200
H	-5.22668500	-1.15385500	1.51263600
H	-4.88232100	1.81123900	-1.59365000
H	-2.51180200	1.23975400	-1.89446600
H	3.20666200	3.15308800	-0.16250500
H	1.81507400	4.02151200	0.47720500
H	2.86410900	3.11800600	1.57259200
H	-0.19933700	1.75528100	3.28891100
H	1.54005800	2.12808100	3.17949300
H	0.36066900	3.25399400	2.50726500

TS7

E_0 = -708.161741, E = -708.142112
Imaginary frequency = 1.

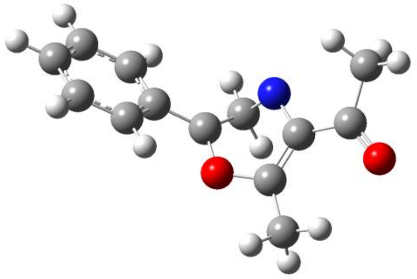

O	0.20707200	1.22940000	0.56321300
C	1.56219300	1.09957300	0.38916900
C	1.92541400	-0.20853100	0.14229900
N	0.97460400	-1.18613000	0.37895800
C	-0.47043100	0.06822300	0.78067300
C	0.23073800	-0.91165400	1.60535200
C	2.29944700	2.38842300	0.33252500
C	3.17521000	-0.59544800	-0.55823900
O	4.16646500	0.13785800	-0.57912800
C	3.19632800	-1.95132800	-1.22976400
C	-1.82173300	0.01260800	0.23518700
C	-2.23725800	0.95134500	-0.73205400
C	-3.53665800	0.91834800	-1.23499900
C	-4.43930300	-0.05632500	-0.79534100
C	-4.03443400	-0.99612600	0.15910400
C	-2.73996700	-0.96255900	0.67462700
H	-5.44890900	-0.08458500	-1.19383500
H	-0.35496900	-1.77155100	1.92171900
H	0.81456500	-0.49238500	2.43572000
H	1.68546600	3.15308100	-0.15474300
H	2.54456700	2.74655700	1.34021100
H	3.23493900	2.25348500	-0.21039400
H	3.31611200	-2.73285600	-0.47082800
H	4.04072500	-1.99226600	-1.91997700
H	2.25718400	-2.15624500	-1.75010900
H	-1.53747400	1.70134700	-1.08320300
H	-3.84318600	1.64913600	-1.97730400
H	-4.72993700	-1.75397300	0.50661300
H	-2.44688900	-1.69418800	1.41952000

TS8

E_0 = -1052.656254, E = -1052.624173
Imaginary frequency = 1.

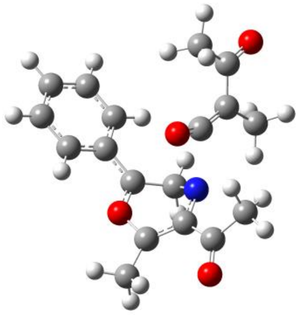

O	-1.00518300	-2.23259100	0.15958800
C	-2.27837900	-1.71160600	0.09937800
C	-2.32605200	-0.32547800	0.25376800
N	-1.27890900	0.33999300	0.78718600
C	-0.02771700	-1.56102400	0.74995200
C	-0.50283800	-0.51533400	1.68433600
C	-3.29161800	-2.71182800	-0.34059100
C	-3.49370600	0.46896300	-0.26145100
O	-4.59661400	-0.05573400	-0.39114900
C	-3.27940300	1.92828800	-0.58283600
C	1.31972600	-1.89236400	0.35188700
C	1.55740200	-2.76559400	-0.73645000
C	2.85718400	-3.11726900	-1.08347800
C	3.94421100	-2.59972200	-0.36686000
C	3.72254700	-1.72919000	0.70633700
C	2.42501800	-1.37717400	1.06752100
H	4.95777600	-2.87056300	-0.64604900
C	0.07860700	1.61134800	-0.58010000
O	-0.08372400	1.06948900	-1.61618100
C	0.63497000	2.51762300	0.25744800
C	0.16420000	2.87474800	1.64522000
C	1.79792700	3.24376600	-0.28060300
O	2.35876400	4.09425700	0.42519000
C	2.31385000	2.96254700	-1.68100800
H	0.30577200	0.04874200	2.14387700
H	-1.13851100	-0.95514000	2.47355300
H	-4.02994800	-2.25008700	-0.99715200
H	-2.79161000	-3.53274000	-0.86278400
H	-3.83669200	-3.13444700	0.51259100
H	-2.82221300	2.45258200	0.25993200
H	-4.24000200	2.38042600	-0.83285700
H	-2.59851400	2.02528000	-1.43516700
H	0.71907400	-3.15604400	-1.30199900
H	3.02712100	-3.78781600	-1.92001300
H	4.56220700	-1.32587100	1.26336100
H	2.27164600	-0.70658500	1.90530000
H	0.92524800	2.63482900	2.39648200
H	-0.01155000	3.95341800	1.70769100
H	-0.75824300	2.35040800	1.88811500
H	3.20058700	3.57368700	-1.85414100
H	2.57130100	1.90640300	-1.81064600
H	1.55739100	3.20598000	-2.43480200

TS9

E_0 = -1052.645276, E = -1052.613380
Imaginary frequency = 1.

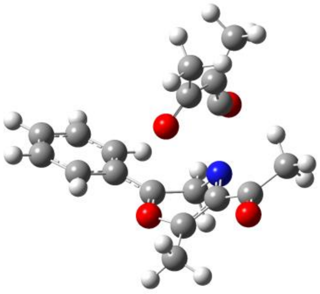

O	-0.70308900	-2.02180600	0.13778300
C	0.62582000	-2.36138300	0.05303100
C	1.42687500	-1.37374100	-0.51976600
N	0.87722100	-0.39716800	-1.26995300
C	-1.21749900	-1.09216300	-0.64691400
C	-0.39527800	-0.80753600	-1.84768900
C	0.95800900	-3.59870100	0.81777200
C	2.90343900	-1.33171200	-0.24958500
O	3.43898000	-2.15792200	0.48586400
C	3.71353200	-0.27490200	-0.96003000
C	-2.45721700	-0.49017700	-0.21783400
C	-2.98834500	-0.76549700	1.06455900
C	-4.20879200	-0.22289100	1.45089800
C	-4.91773800	0.61390600	0.57872300
C	-4.39859500	0.90105500	-0.68869300
C	-3.18158100	0.35540000	-1.08884700
H	-5.86619800	1.04266900	0.88744800
C	0.76282600	1.88054800	-1.07398800
O	0.33683000	2.06673800	-2.15899100
C	1.29012900	2.31831700	0.09735600
C	1.84164400	3.74666900	0.01200200
C	1.26597500	1.59877200	1.36666700
O	0.63656900	0.55096800	1.56640700
C	2.04382300	2.23005100	2.51262800
H	-0.79894400	-0.00971400	-2.46842300
H	-0.28234200	-1.72216000	-2.45929300
H	1.21817300	-3.37182800	1.85972300
H	0.09725700	-4.27327200	0.81692900
H	1.81350700	-4.10965700	0.37573100
H	3.69012100	-0.45504900	-2.04050700
H	4.74348900	-0.31182700	-0.60229200
H	3.28575200	0.71777300	-0.79677100
H	-2.43304100	-1.39803700	1.74734400
H	-4.60573900	-0.44163200	2.43732200
H	-4.94385300	1.55003000	-1.36669400
H	-2.79655500	0.58452500	-2.07602500
H	1.74186700	4.14710000	-0.99917400
H	1.30831900	4.42296800	0.68924800
H	2.90560200	3.77511600	0.27189000
H	1.98378600	1.57467200	3.38264600
H	3.09438800	2.38918800	2.24575300
H	1.62737900	3.20939300	2.77458000

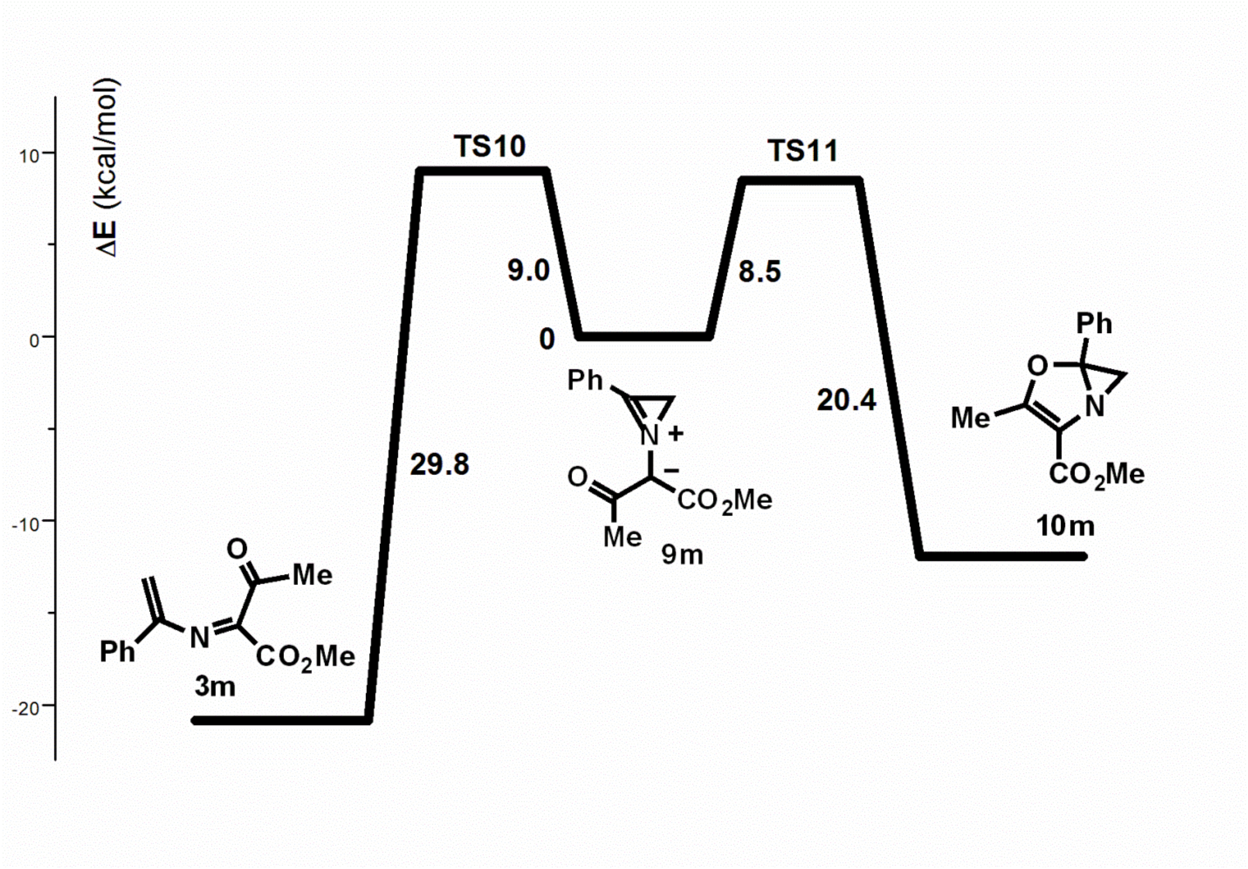

Figure S1. Energy profile [DFT B3LYP/6-31+G(d,p), 357 K, 1,2-dichloroethane (PCM)] for the transformation of ylide **9m** into azadiene **3m** and dihydroazireno[2,1-*b*]oxazole **10m**.

Table S2: Energies and Cartesian coordinates of compounds **3m, 9m, 10m** and transition states TS10,TS11

Azadiene **3m**

E_0 = -783.381832, E = -783.359210
Imaginary frequency = 0.

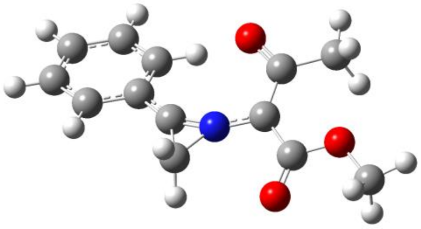

N	0.32340600	-0.68050200	0.11813700
C	1.44592500	0.11690200	0.06185200
C	-0.94141100	-0.89939800	0.14481100
C	-0.08742900	-2.08632800	0.36565000
C	2.67952200	-0.62468400	-0.15793800
C	1.24349500	1.53330300	0.28379100
C	-2.27410300	-0.39396700	0.01349100
C	-3.33147800	-1.29134100	0.28407800
C	-4.65314200	-0.87623400	0.14634800
C	-4.93621400	0.42987100	-0.27083800
C	-3.89150400	1.32021800	-0.55054900
C	-2.56474400	0.91960800	-0.41542200
O	2.73533600	-1.82326700	-0.43896500
O	3.79714500	0.12993300	-0.01871900
O	0.11057400	1.95390200	0.59753400
C	2.38221100	2.52592000	0.14515500
H	0.11231600	-2.78160300	-0.44526800
H	0.06811500	-2.46422600	1.37641100
H	-3.10448800	-2.30405800	0.60273300
H	-5.46140900	-1.56842600	0.36043600
H	-5.96725500	0.75147700	-0.38292000
H	-4.11415100	2.33091300	-0.87869000
H	-1.75001000	1.60366800	-0.61448600
H	3.10293800	2.40495800	0.95956100
H	1.95547600	3.52965400	0.18813800
H	2.93299800	2.39097100	-0.78872400
C	5.04974500	-0.53499100	-0.27222900
H	5.07659200	-0.93062300	-1.29010300
H	5.20309200	-1.34922600	0.43950700
H	5.81225800	0.23222100	-0.14157000

Ylide **9m**

E_0 = -783.415691, E = -783.392405
Imaginary frequency = 0.

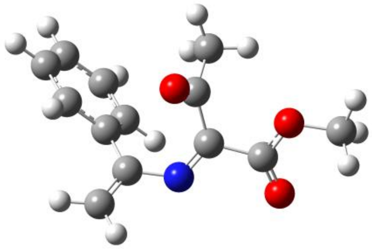

N	-0.51403500	1.40393800	-0.35881800
C	-1.18214800	0.38549000	0.02023800
C	0.82546200	1.65556100	0.03884100
C	1.11813900	2.88476700	0.50133300
C	-2.60420700	0.28090000	-0.50546600
C	-0.72649800	-0.66535100	1.04510900
C	1.85853200	0.59839700	-0.16663500
C	2.83902900	0.34868900	0.80838400
C	3.83782500	-0.60159800	0.58341300
C	3.87359800	-1.31545600	-0.61837000
C	2.90136300	-1.07490200	-1.59476700
C	1.89918000	-0.12878300	-1.37028300
O	-3.05504000	0.92654600	-1.42841500
O	-3.29287800	-0.62269700	0.20622200
O	-0.55930700	-0.29911700	2.19736300
C	-0.51315100	-2.08005900	0.58109600
H	0.34570100	3.64029300	0.59721100
H	2.12490600	3.13773800	0.81225600
H	2.80723500	0.88777300	1.74997400
H	4.58253600	-0.78929100	1.35131200
H	4.65067700	-2.05384700	-0.79198300
H	2.92495200	-1.61974300	-2.53394800
H	1.15656400	0.05964600	-2.14014700
H	-1.43077600	-2.47124500	0.13125400
H	-0.21516500	-2.70413000	1.42536900
H	0.26508200	-2.10101200	-0.18951400
C	-4.68105400	-0.82308900	-0.15686300
H	-4.74791300	-1.17418100	-1.18816700
H	-5.23218400	0.11208400	-0.04404300
H	-5.05199600	-1.57690200	0.53460500

Azirenooxazole **10m**

E_0 = -783.399602, E = -783.378241
Imaginary frequency = 0.

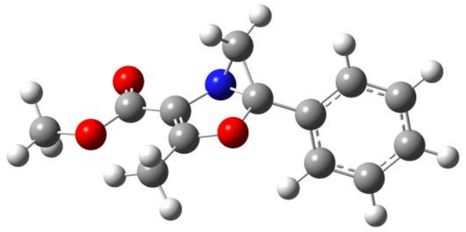

N	0.30973900	-0.99538900	0.24613000
C	1.46129700	-0.13105500	0.15725300
C	-0.75756300	-0.09758300	0.72853300
C	-0.21676200	-1.12207000	1.64657700
C	2.71633100	-0.69386000	-0.33880800
C	1.13521200	1.13702800	0.52748100
C	-2.12929800	-0.06954500	0.14009300
C	-3.21475600	-0.61977500	0.83153100
C	-4.49668500	-0.58593100	0.27362100
C	-4.69928900	0.00178600	-0.97768900
C	-3.61745400	0.55843500	-1.67063600
C	-2.33847100	0.52439600	-1.11408700
O	2.86236700	-1.86843700	-0.65167400
O	3.71802500	0.21403100	-0.41118500
O	-0.18870400	1.22083800	0.86034300
C	1.88265400	2.41712800	0.62539800
H	-0.77000200	-2.03793400	1.82481100
H	0.42974000	-0.78794800	2.45437200
H	-3.06055300	-1.07143100	1.80685100
H	-5.33324800	-1.01425300	0.81752700
H	-5.69435700	0.02825600	-1.41199700
H	-3.77113900	1.01585000	-2.64349800
H	-1.50011800	0.95628100	-1.65259700
H	2.91379600	2.28961700	0.30430400
H	1.86574500	2.77688200	1.66028400
H	1.39601800	3.17848600	0.00559800
C	4.99184200	-0.27769600	-0.87880700
H	4.90091200	-0.66337800	-1.89644700
H	5.36171000	-1.06575300	-0.21939500
H	5.65747800	0.58404600	-0.85535900

TS10

E_0 = -783.367269, E = -783.344918
Imaginary frequency = 1.

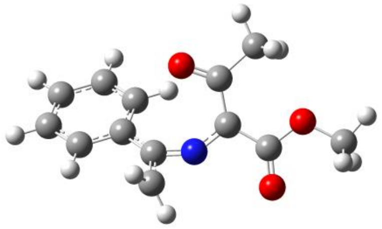

N	0.37368600	-0.95557700	0.05839800
C	1.34818600	-0.03174800	0.09761300
C	-0.89376300	-1.13843300	0.21405600
C	-0.49854900	-2.29872000	0.96452600
C	2.65408600	-0.56178100	-0.32508700
C	1.05089800	1.27296100	0.70610500
C	-2.13530700	-0.45257500	-0.06223700
C	-3.33478900	-1.07102500	0.34135100
C	-4.55948000	-0.46515600	0.07066200
C	-4.59826100	0.74444300	-0.63320100
C	-3.40990000	1.35064700	-1.05987200
C	-2.18015400	0.76346300	-0.77254800
O	2.83846000	-1.71437200	-0.70812000
O	3.65901800	0.34207300	-0.26286800
O	-0.05764700	1.45640500	1.23408000
C	2.06753100	2.39796400	0.71514800
H	-0.02818100	-3.14019000	0.47756600
H	-0.69733500	-2.35445800	2.03475300
H	-3.29775100	-2.02549000	0.85844500
H	-5.48129200	-0.93878800	0.39331100
H	-5.55340100	1.21064800	-0.85565800
H	-3.44460600	2.28511800	-1.61114500
H	-1.26013600	1.23894200	-1.08901600
H	2.91702000	2.14762900	1.35740100
H	1.57362300	3.29298000	1.09761900
H	2.47122100	2.58666000	-0.28266800
C	4.95937400	-0.11741800	-0.68188100
H	4.93081700	-0.45321700	-1.72080100
H	5.30060200	-0.93524600	-0.04334600
H	5.61574700	0.74586900	-0.57779000

TS11

E_0 = -783.367420, E = -783.345700
Imaginary frequency = 1.

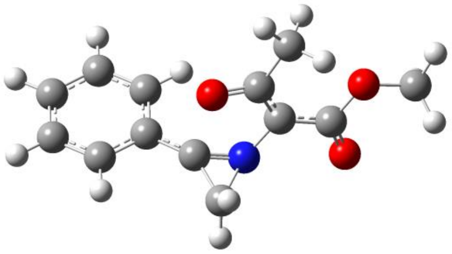

N	-0.36051100	1.12138800	0.15309900
C	-1.39019600	0.13754100	0.13898600
C	0.87743300	0.84418000	0.51645800
C	0.21245800	1.85222300	1.35062900
C	-2.63917000	0.51603500	-0.47026300
C	-1.01532300	-0.99606400	0.90101500
C	2.12128300	0.33485500	0.00980700
C	3.31592000	0.66693700	0.67461700
C	4.53407000	0.20028200	0.18365800
C	4.56398300	-0.58896500	-0.97131300
C	3.37547400	-0.91720400	-1.63871000
C	2.15408300	-0.46393100	-1.15067700
O	-2.85212600	1.59787400	-1.02358400
O	-3.59727700	-0.45021400	-0.39141100
O	0.15873400	-1.03347400	1.39220400
C	-1.94253200	-2.15753400	1.18823900
H	0.35518100	2.91424000	1.17813600
H	-0.09542300	1.54502800	2.34842800
H	3.27981500	1.28688900	1.56499700
H	5.45703300	0.45324500	0.69544000
H	5.51403500	-0.94839700	-1.35501600
H	3.40671200	-1.52782800	-2.53542100
H	1.22845400	-0.71686500	-1.65738100
H	-2.85673500	-1.81905800	1.68164100
H	-1.42108800	-2.87475500	1.82333800
H	-2.24344100	-2.64418500	0.25646700
C	-4.86929700	-0.13204000	-0.98452800
H	-4.76019900	0.05654300	-2.05526500
H	-5.31209000	0.74438500	-0.50530200
H	-5.49243900	-1.01033700	-0.81692500

References

1. Sheldrick, G. M. *Acta Crystallogr., Sect. A* **2008**, *64,* 112–122.

2. Dolomanov, O. V.; Bourhis, L. J.; Gildea, R. J.; Howard, J. A. K.; Puschmann, H. *J. Appl. Crystallogr.* **2009**, *42*, 339–341.

3. Fowler, F. W.; Hassner, A.; Levy, L. *J. Am. Chem. Soc*. **1967**, *89*, 2077–2082.

4. Rostovskii, N. V.; Novikov, M. S.; Khlebnikov, A. F.; Khlebnikov, V. A.; Korneev, S. M. *Tetrahedron* **2013**, *69*, 4292–4301.

5. Leonard, N. J.; Zwanenburg, B. *J. Am. Chem. Soc*. **1967**, *89*, 4456–4465.

6. Hortmann, A.; Robertson, D.; Gillard, B. *J. Org. Chem.* **1972**, *37*, 322–324.

7. Broun, D.; Brown, G. A.; Andrews, M.; Large, J. M.; Urban, D.; Butts, C. P.; Hales, N. J.; Gallagher, T. *J. Chem. Soc., Perkin Trans. 1* **2002**, 2014–2021.

8. Schulthess, A. H.; Hansen, H.-J. *Helv. Chim. Acta* **1981**, *64*, 1322–1336.

9. Jiang, Y.; Khong, V. Z. Y.; Lourdusamy, E.; Park, C.-M. *Chem. Commun*. **2012**, *48*, 3133–3135.

10. Wilds, A. L.; Mearder, A. L. *J. Org. Chem*. **1948**, *13*, 763–769

11. Presset, M.; Mailhol, D.; Coquerel, Y.; Rodriguez, J. *Synthesis* **2011**, *16*, 2549–2552.

12. Davies, J. R.; Kane, P. D.; Moody, C. J. *Tetrahedron* **2004**, *60*, 3967–3977.

13. Frisch, M. J.; Trucks, G.W.; Schlegel, H. B.; Scuseria, G. E.; Robb, M. A.; Cheeseman, J. R.; Scalmani, G.; Barone, V.; Mennucci, B.; Petersson, G. A.; Nakatsuji, H.; Caricato, M.; Li, X.; Hratchian, H. P.; Izmaylov, A. F.; Bloino, J.; Zheng, G.; Sonnenberg, J. L.; Hada, M.; Ehara, M.; Toyota, K.; Fukuda, R.; Hasegawa, J.; Ishida, M.; Nakajima, T.; Honda, Y.; Kitao, O.; Nakai, H.; Vreven, T.; Montgomery, J. A.; Peralta, J. E.; Ogliaro, F.; Bearpark, M.; Heyd, J. J.; Brothers, E.; Kudin, K. N.; Staroverov, V. N.; Kobayashi, R.; Normand, J.; Raghavachari, K.; Rendell, A.; Burant, J.

C.; Iyengar, S. S.; Tomasi, J.; Cossi, M.; Rega, N.; Millam, N. J.; Klene, M.; Knox, J. E.; Cross, J. B.; Bakken, V.; Adamo, C.; Jaramillo, J.; Gomperts, R.; Stratmann, R. E.; Yazyev, O.; Austin, A. J.; Cammi, R.; Pomelli, C.; Ochterski, J. W.; Martin, R. L.; Morokuma, K.; Zakrzewski, V. G.; Voth, G. A.; Salvador, P.; Dannenberg, J. J.; Dapprich, S.; Daniels, A. D.; Farkas, Ő.; Foresman, J. B.; Ortiz, J. V.; Cioslowski, J.; Fox, D. J. Gaussian 09, Revision C.01; Gaussian: Wallingford CT, 2010.
